# Cooperative Catalytic Coupling of Benzyl Chlorides and Bromides with Electron-Deficient Alkenes

**DOI:** 10.1021/acs.orglett.4c01413

**Published:** 2024-06-19

**Authors:** Roshini Hanumanthu, Jimmie D. Weaver

**Affiliations:** 107 Physical Science, Department of Chemistry, Oklahoma State University, Stillwater, Oklahoma 74078, United States

## Abstract

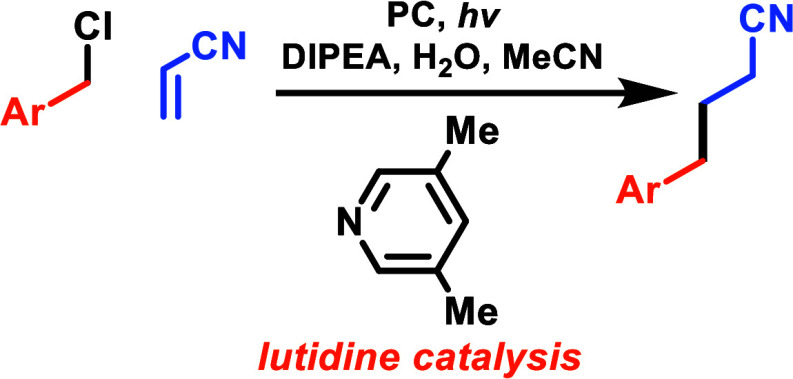

Benzyl radicals are an important class of intermediate. The use of visible light to generate them directly from their respective halides is an ideal synthetic strategy. The central impediment associated with their direct single-electron reduction (photo- or electro-) lies in their highly variable and structurally dependent reduction potential, which combine to make the identification of a general set of conditions difficult. Herein, we have employed a strategy of nucleophilic cooperative catalysis in which catalytic lutidine undergoes halide substitution, which decreases and levels the reduction potential. This allows a general set of photocatalytic conditions to transform a broad range of benzyl halides into radicals that can be used in the synthesis of more complex molecules, exemplified here by Giese coupling with electron-deficient alkenes.

Among the various open-shell species, carbon-centered radicals are fascinating intermediates capable of facile bond formation. As methods to access them have increased, so too has their use in organic synthesis.^1−4^ One way to generate such radicals is through the homolytic cleavage of the corresponding, relatively weak, C–X bonds. Conveniently, organic halides are key commodity chemicals with increasing availability moving upward in the periodic table toward chlorine.^5^ Despite their relatively weak bonds, direct photolysis requires use of UV light, which significantly limits the synthetic utility of any method (Scheme 1A).^6^ Alternatively, single-electron reduction, mediated by a photocatalyst or an electrode, to populate the antibonding orbital can generate the desired carbon-centered radical with extrusion of halide anion (Scheme 1B). For benzyl chlorides, which are the most commercially abundant of the halides, however, this requires fairly negative reduction potentials^7^ (*E*_red_ < −2 V vs SCE^8^). Although this approach has worked well in the case of related aryl halides,^9,10^ this deep reduction potential is often functional group limiting.^11^ Another route to activate halides is through halogen-atom transfer (XAT) reactions (Scheme 1C).^12^ Leonori recently demonstrated the use of α-amino radicals to facilitate XAT and generate C-centered radicals—though this method appears limited to iodides and bromides.^13^ Melchiorre^14,15^ and others^16^ have sought to activate recalcitrant C–X bonds by exploiting the inherent electrophilicity of alkyl halides to be replaced by a nucleophile that can also serve as a chromophore (Scheme 1D).^17,18^ Building on this idea,^19^ work in our lab has shown that when the halide is displaced with collidine (2,4,6-trimethylpyridine) to generate a bench-stable collidinium salt, it can be photocatalytically converted to the radical via electron capture (Scheme 1E, center).^17^ The substituents located at the 2,4,6-position served to both protect the pyridine core from undergoing functionalization (Minisci-type reaction) and likely aided in fragmentation of the C–N bond.^21^ Of note, there has been significantly more work done with Katritzky salts^20,21^ (2,4,6-triphenylpyridinium) which, unlike collidinium salts, are prone to form electron donor–acceptor (EDA) complexes in the visible region (Scheme 1E, left). Another critical difference is the enhanced nucleophilicity of collidine compared to that of 2,4,6-triphenylpyridine. The latter is unable to displace halides to form salts. Rather, these salts are formed by condensation with a primary amine and a pyrylium salt. More recently, Wengryniuk has developed a mild oxidative approach to the formation of collidinium salts which takes place by the electro-oxidative C–H functionalization of electron-rich arenes and further expands the scope of collidinium salts (Scheme 1E, right).^22^

**Scheme 1:**
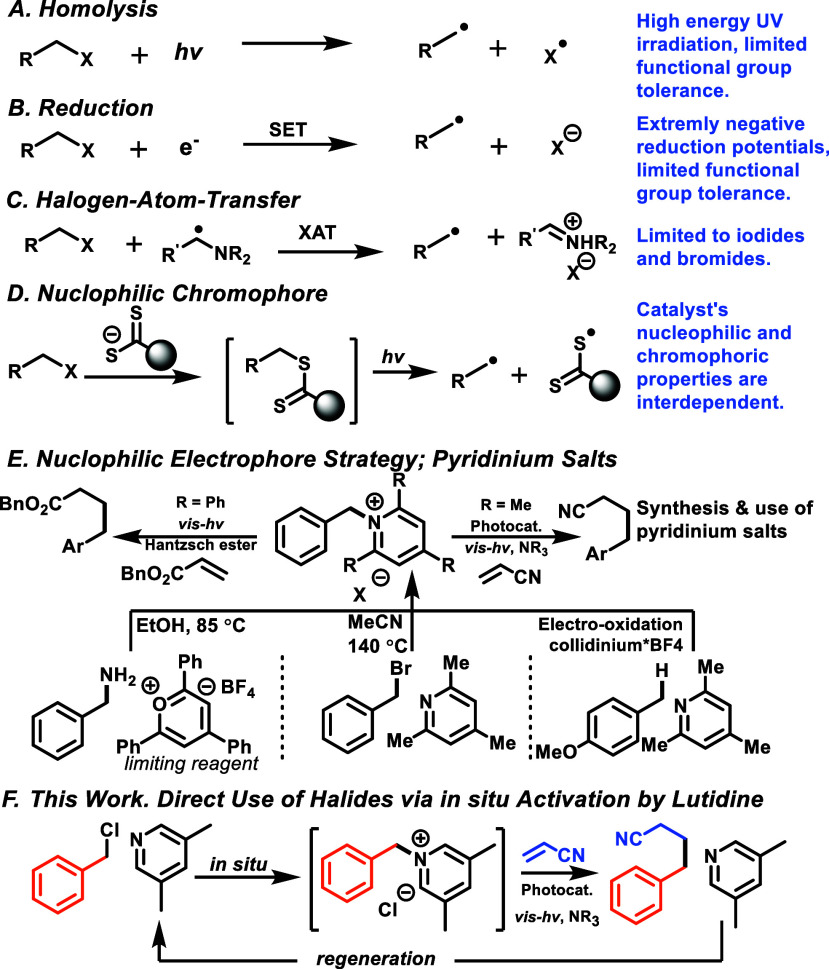
Halide Activation Strategies

Ideally, collidine could be used catalytically to convert the benzyl halide. Unfortunately, we found the rate of substitution with many benzylic halides to be insufficient at near-ambient temperatures to allow cocatalysis such that it was necessary to first form the collidinium salt and then use it stoichiometrically. Aside from the prefunctionalization step and stoichiometric collidine, there were also limitations of scope, due to competing elimination reaction during the salt formation, creating a salty conundrum. We suspected that the methyl groups at the ortho position of collidine were hindering nucleophilicity of the N-atom, but these groups were shown to be needed to prevent undesired alkylations of the pyridine core.^23^ Based on precedent in the literature,^24^ which suggested that *meta*-substitution would largely prevent radical addition to the adjacent positions, we proposed that we could use the enhanced nucleophilicity of lutidine (3,5-dimethylpyridine) to activate benzyl halides *in situ* via salt formation and subsequent radical formation. Furthermore, because it should be regenerated after fragmentation, it should be possible to use lutidine in catalytic amounts (Scheme 1F).

To assess lutidine’s viability, we conducted a competition experiment wherein excess of benzyl chloride was reacted with collidine and lutidine at several temperatures (Table 1). While no salt formation from either pyridine derivative was observed at room temperature, when we used conditions that had previously been shown to be optimal for formation of the collidinium salts but at lowered temperature (4 h at 70 °C),^17^ we observed exclusive formation of lutidinium salts—reflecting the enhanced nucleophilicity of lutidine and suggesting that cocatalysis might be feasible.

**Table 1:**
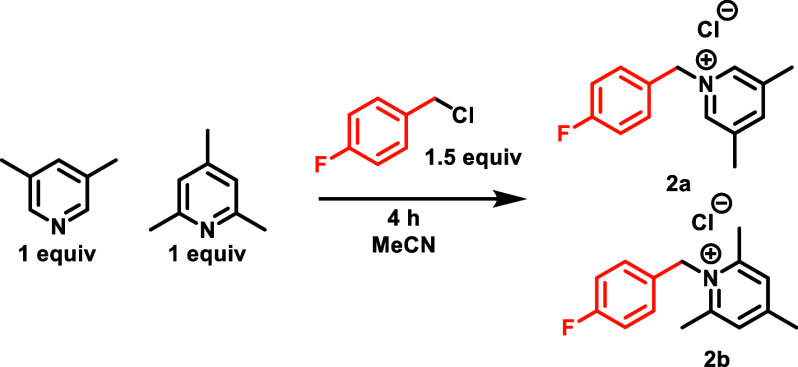
Search for Nucleophile to Achieve Co-Catalysis

**entry**	**temp°C**	**yield (2a)**	**yield (2b)**
**1**	120 °C	95%	0%
**2**	70 °C	83%	0%
**3**	Rt	0%	0%

Using lutidine as a nucleophilic cocatalyst, we aimed to optimize the reaction conditions for a Giese-coupling of benzyl chloride and acrylonitrile. From a process perspective, lutidine is nearly ideal. It costs $30/mol,^25^ is easily handled, can be washed away in workup, and has the potential to be used catalytically. Thus, we started optimizing the reaction using the strongly oxidizing photocatalyst Ir[dF(CF_3_)ppy]_2_(dtbbpy)PF_6_,^26,27^ diisopropylethylamine (DIPEA) as reductant, irradiation from blue LEDs, and catalytic lutidine. With 20 mol % of lutidine (**entry 1**, Table 2), we observed full consumption of the halide within 4 h, and after an additional 10 h of irradiation, we saw the complete conversion of the *in situ* formed lutidinium salt to yield the desired product with NMR yield of 49%. Increasing the lutidine from 0.2 to 0.7 equiv, we saw an increase in yield from 49% to 70% (**entry 2**), which we used for our subsequent optimization. The primary competing reaction appeared to be the formation of radical propagation products (**3a′** and **3a″**). In our previous study with collidinium salts, water played a significant role in the reaction outcome; thus, we investigated the role of water. Decreasing the water loading to 10 equiv (**entry 3**) resulted in the formation of unidentified products and no product formation, highlighting the significance of H_2_O in steering the reaction toward the intended product. Further exploration of the H_2_O loading showed that augmenting the water loading to 300 equiv^28^ provided the most convenient handle for diminishing the propagation products while simultaneously increasing the yield (**entry 5**). Next, we observed the effect of DIPEA loading on the reaction. Low loadings of DIPEA gave us diminished results, establishing the fact that a tertiary amine is crucial (**entry 6**). Increasing the equivalents of acrylonitrile from 2 to 3 gave a further increase in yield to 84% (**entry 7** vs **2**) while additional alkene resulted in reduced yields (**entry 8**), due to the formation of propagation products. Next, we returned to optimal lutidine loadings. Both 20 and 40 mol % (**entries 9** & **10**) gave nearly optimal yields (73% and 78%, respectively)—indicating the feasibility of catalyst turnover. Further increases in lutidine loading (**entries 11** and **12**) give little additional benefit. When the reaction was conducted at room temperature (**entry 13**), it required 72 h for complete conversion of the chloride and resulted in a 39% yield, illustrating that the reaction could, in principle, proceed at lower temperatures—albeit at the expense of reaction rate and yield.^29^ Individual control experiments in which amine or lutidine was left out of the reaction mixture indicated that both are critical reaction components (**entry 14**). With an understanding of the reaction parameters, we then explored the scope of the benzyl halides (Scheme 2). The optimal conditions worked well for a range of benzylic halides with electron-withdrawing groups (**3e**, **3f**, **3h**, **3j**, **3p**, and **3u**), electron-neutral groups (**3g**, **3s**), and even the electron-donating halides with more negative reduction potentials (**3c**, **3i**, **3m**, **3o**, and **3r**) gave excellent yields. There was very little decrease in reactivity when the reaction was conducted on a scale of 1 mmol (**3a**). On average, the use of the halides provided a significant enhancement in yield compared to the premade collidinium salt conditions,^17^ resulting in an average increase of 12% yield across 11 substrates.^30^ Sterically hindered substrates, with ortho substituents that often make substitution challenging (**3b**, **3l**, and **3m**), worked exceptionally well with no changes to optimized conditions, highlighting the enhanced nucleophilicity of lutidine. Nitroarenes, which are frequently not tolerated with Ir-photocatalysts, gave the expected product in excellent amounts (**3f**). The mild reaction conditions exhibited a high degree of tolerance toward a range of functional groups such as nitrile (**3h**), nitro (**3f**), ester (**3j**), ether (**3i**), bromide (**3e**), and fused ring systems (**3k**, **3p**, and **3t**). The excellent performance we observed across a range of benzylic halides—both chlorides and bromides, whose reduction potentials vary by more than 1 V, illustrates the advantage of this approach, that otherwise would likely not work well under a single set of conditions. Sensitive heterocycles like thiophene (**3d**) and naphthalene^31^ (**3k**) which might otherwise undergo radical addition gave desired products in good yields. Dichlorinated xylenes (**3n**) afforded the diaddition product in excellent yields. Next, we wanted to expand the scope to include more challenging secondary and tertiary benzylic halides. In this set of conditions, it was observed that the majority of the secondary benzyl halides exhibited a preference for substitution rather than elimination. Due to the increased nucleophilicity of lutidine, the scope could be extended to include secondary bromides and chlorides (**3p**, **3q**, **3r**, **3s**, **3t**, **3u**), with generally good to excellent yields. Even a tertiary (**3v**) benzylic chloride proved feasible—albeit with significantly diminished yield; however, with collidinium salts not even this was possible.^32,33^ In the case of **3v**, the radical propagation product seems to be much more prevalent, suggesting that the increased steric demand of the radical group retards the rate of the termination step. Previously, our attempts to use collidine on such substrates led almost exclusively to elimination.^17^ Importantly, except for **3h**, **3f**, **3j**, and **3p**, which could theoretically be reduced by the photocatalyst, for all of these substrates direct reduction by photocatalyst is not feasible, but, beyond reaction time, they were made to react using a standard set of conditions, highlighting the leveling effect induced by the formation of the lutidinium salt. Next, we explored the use of benzylic tosylates as the radical source and found that they too gave the desired product (**3g**) in excellent yield, providing a convenient approach to convert alcohols into radicals. When more process-friendly benzyl mesylate was utilized, it gave the desired **3g** in more moderate yield, with the radical propagation as the major product, suggesting that while feasible, further optimization is needed before mesylates can be utilized as radical precursors.

**Table 2:**
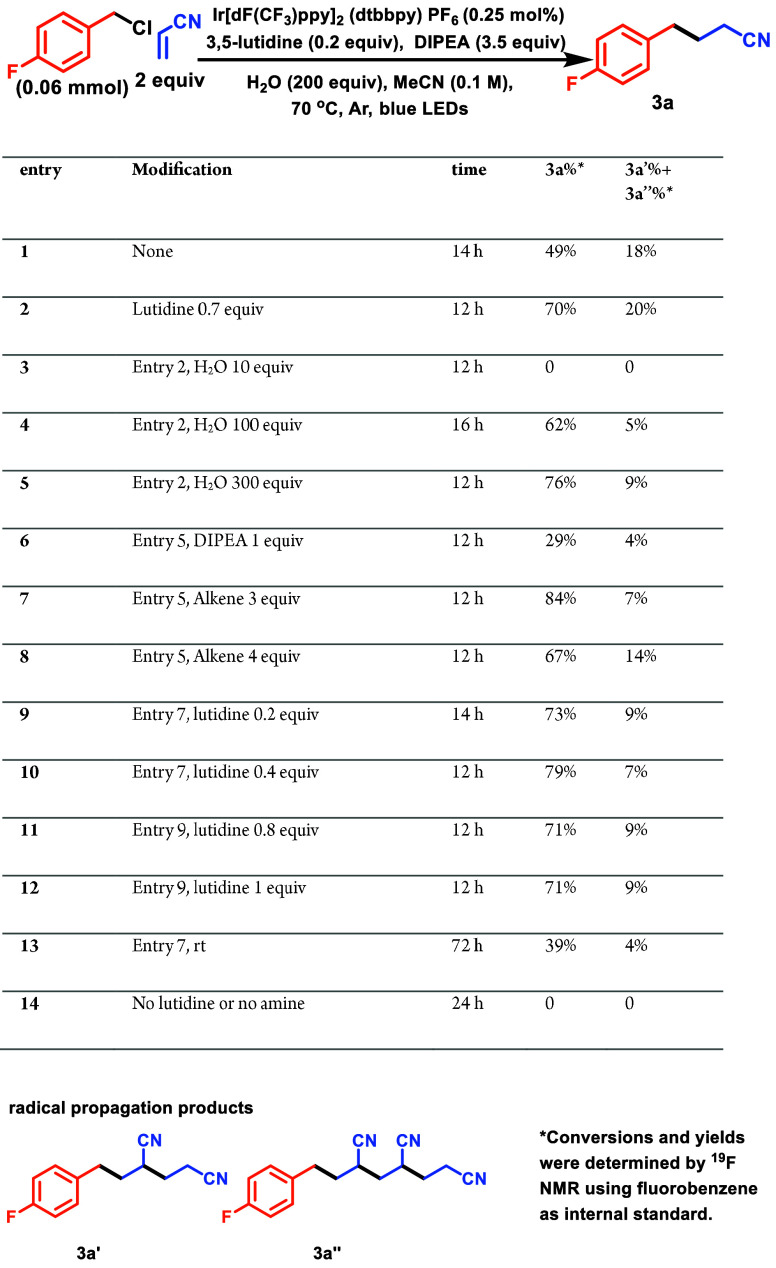

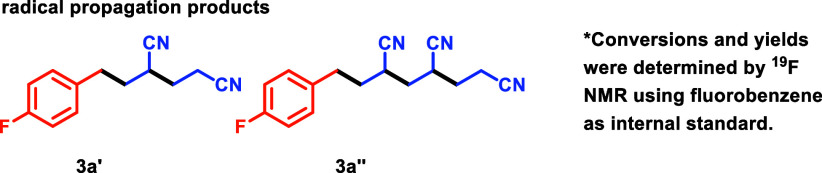
Optimization Table

**Scheme 2:**
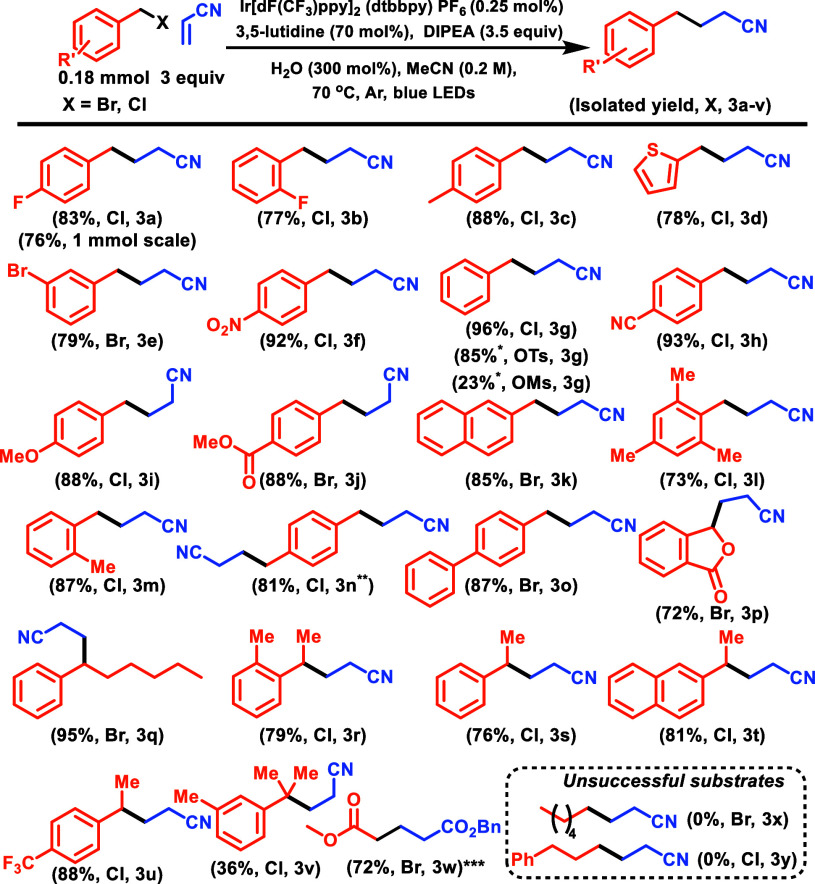
Substrate Scope Yields are isolated products unless otherwise noted. *^1^H NMR yield using fluorobenzene as internal standard. **Double the amounts of alkene, DIPEA, water, and lutidine were added. ***Reaction set up using benzyl acrylate as alkene.

Next, we wanted to see if we can generate less stabilized alkyl radicals from their halides via a similar mechanistic pathway. We found out that alpha-carbonyl halides such as methyl 2-bromoacetate gave excellent yields with optimized conditions (**3w**). Unfortunately, when hexyl bromide (**3x**) and (3-chloropropyl)benzene (**3y**) were subjected to these reaction conditions, no desired product formation was observed after 12 h of irradiation. Reactions setup with the corresponding lutidinium salts also failed to produce the desired alkylation product, suggesting that the ability to form radicals is correlated to the stability of the radical fragment, given that the analogous reaction worked using the alpha-bromoacetate (**3w**) which forms a more stabilized radical.

To better understand the reaction, several mechanistic experiments were performed. Monitoring the reaction over time confirmed the intermediacy of an *N*-*para*-fluoro-benzyl lutidinium salt in significant quantities (see Supporting Information). Further, starting with premade *N*-*para*-*F*-benzyl lutidinium salt, rather than the corresponding halide, showed conversion to the desired product (62% NMR yield within 12 h, with fluorobenzene serving as the internal standard, see Supporting Information). Taken together, along with the leveling of the reduction potential, it seems reasonable that the lutidinium salt is the precursor to the radical.

Our working mechanism is shown in Scheme 3. The first step is the absorption of blue photon by the photocatalyst to produce strongly oxidizing Ir(III)* ([Ir*(III)/Ir (II) = +1.42 V vs SCE in MeCN),^34^ which undergoes reductive quenching by the amine^17^ (measured *E*_1/2_ = 0.61 V vs SCE) to give an Ir(II) species. Meanwhile, in the nucleophilic catalytic cycle, lutidine displaces the benzylic halide to form a lutidinium salt (***int*****-A**). Next, the reduced photocatalyst (Ir(II/III) = −1.37 V vs SCE,^35^ in 0.1 M TBAH/MeCN) undergoes a rate-determining and slightly endothermic SET to the lutidinium salt (measured *E*_1/2_ = −1.46 V vs SCE in MeCN), generating the lutidinium radical (***int*****-B**) and returning the photocatalyst to its initial state. ***Int*****-B** undergoes a unimolecular fragmentation of the C–N bond to generate the benzylic radical^36^ (***int*****-C**) and lutidine—completing the nucleophilic catalytic cycle.

**Scheme 3:**
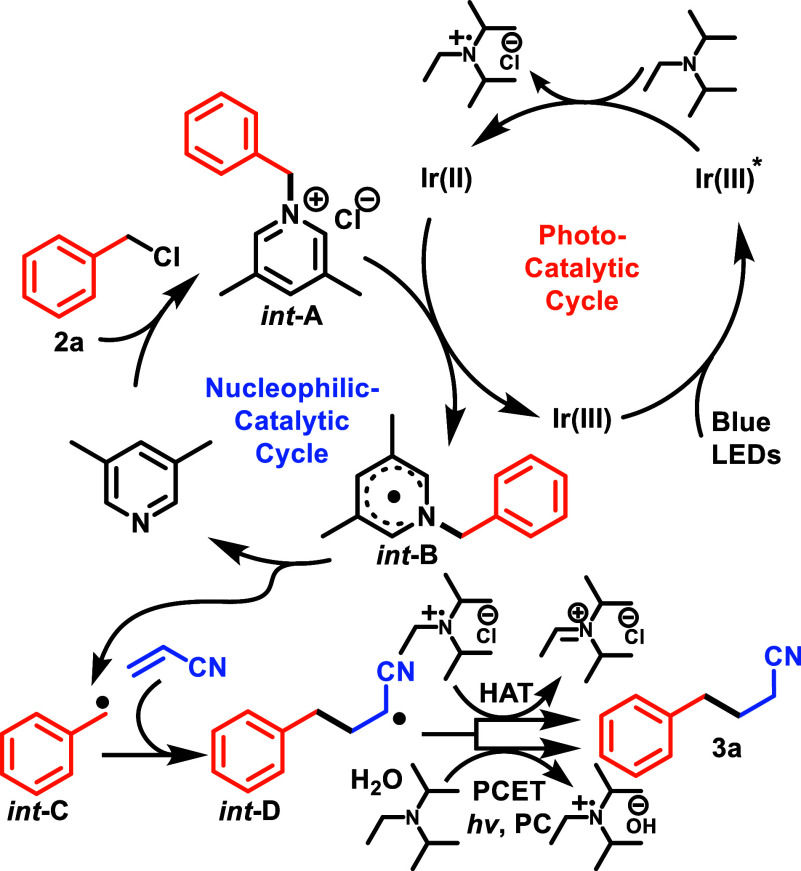
Mechanism

***Int*****-C** reacts with the electron-poor acrylonitrile to yield radical intermediate, ***int*****-D**, which then yields the product after hydrogen atom transfer from amine radical cation—though a radical polar crossover step or proton-coupled electron transfer may be involved in this final step.^37,38^

In conclusion, we have developed an efficient catalytic method to produce functionalized benzylic radicals directly from the chlorides, bromides, and tosylates, many of which could not be conveniently activated by the more standard singular mechanistic manifold. The cooperative catalytic approach was essential to the success of the reaction, circumventing both deep reductions and dubious isolation of the intermediate electrophores. As a result, previously sluggish and unreactive benzylic halides can be engaged using sub-stoichiometric and inexpensive lutidine with a photocatalyst to generate the benzylic radicals. We have shown that the expansion of scope to benzyl tosylates and mesylates is feasible, although further investigation in this direction is warranted. We anticipate that lutidine will prove generally useful for exploiting the electrophilic tendency of molecules to enhance their ability to capture an electron and ultimately convert them into odd-electron species capable of forming bonds.

## Data Availability

The data underlying this study are available in the published article and its Supporting Information.

## Supporting Information Available

Detailed experimental procedures, optimization study, complete characterization data, and copies of NMR spectra (PDF)
FAIR data, including the primary NMR FID files, for compounds **3a**, **3b, 3c, 3d, 3e, 3f, 3g, 3h, 3i, 3j, 3k, 3l, 3m, 3n, 3o, 3p, 3q, 3r, 3s, 3t, 3u**, **3v**, **3w** (ZIP)
