# Supplementary material for: Cooperative Catalytic Coupling of Benzyl Chlorides and Bromides with Electron-Deficient Alkenes
Source: Org Lett. 2024 Jun 19;26(25):5248–52. doi: 10.1021/acs.orglett.4c01413 (PMC11217938; doi:10.1021/acs.orglett.4c01413)

# Cooperative Catalytic Coupling of Benzyl Chlorides and Bromides with Electron Deficient Alkenes

Roshini Hanumanthu and Jimmie D. Weaver III*

Department of Chemistry, Oklahoma State University, OK 74078

jimmie.weaver@okstate.edu

**Supporting information**

**Table of Contents:**

**General experimental** S2

**Synthesis of substrates** S3

**Optimization of reaction conditions** S6

**Mechanistic experiments** S8

**Electrochemical measurements** S12

**Photocatalytic reactions** S15

**1 mmol Scale Photocatalytic reactions** S25

**References** S26

**NMR and MS spectra** S27

**General Experimental:**

All reagents were obtained from commercial suppliers (Aldrich, VWR, TCI Chemicals, and Oakwood Chemicals) and used without further purification unless otherwise noted. Acetonitrile ($CH_3CN$) was dried for 48 h over activated 3 Å molecular sieves. Distilled diisopropylethylamine was stored over KOH pellets under an argon atmosphere in an amber bottle.

Reactions were monitored by a combination of thin layer chromatography (TLC), (obtained from sorbent technologies Silica XHL TLC Plates, w/UV254, glass backed, 250 µm, 20 x 20 cm) and were visualized with ultraviolet light, potassium permanganate stain, GC-MS (Chemical Ionization, QP 2020NX Shimadzu, equipped with an autosampler), 19F NMR and 1H NMR (vide infra). Purifications were conducted using a Teledyne Isco Combiflash Rf 200i flash chromatograph with Sorbtech Rf normal-phase silica columns (4 g, 12 g, or 24 g) and product detection at 254 and 288 nm. NMR spectra were obtained on a 400 MHz Bruker Avance III spectrometer or Bruker Neo 800 MHz spectrometer equipped with a TCI cryo-probe.1H, 19F and 13C NMR chemical shifts are reported in ppm relative to the residual protio solvent peak (1H, 13C). Mass spectra (HRMS) analysis was performed on LTQ-OrbitrapXL by Thermo Scientific ltd using heated electrospray ionization (H-ESI) source.

**Photocatalytic Reaction Setup**

Photocatalytic reactions were carried out in a light bath which consists of high-intensity blue LEDs (λ max emission ~ 450 nm) as described below. The blue LED strips (200 LEDs) were wrapped around the walls of a Pyrex crystallizing dish (capacity 1,200 mL, O.D. × H ~150 mm × 75 mm). A lid that rested on the top was fashioned from cardboard and holes were made such that NMR tubes were held firmly in the cardboard lid which was placed on the top of the bath. Water was added to the bath such that the tubes were submerged in the water, maintained at 70 °C with a thermostat-controlled heating mantle. Lights are bought from Solid Apollo Blue Waterproof 5050 7 Blue LED Strip Light. SKU: SA-LS-BL-5050-300-IP67-24V.

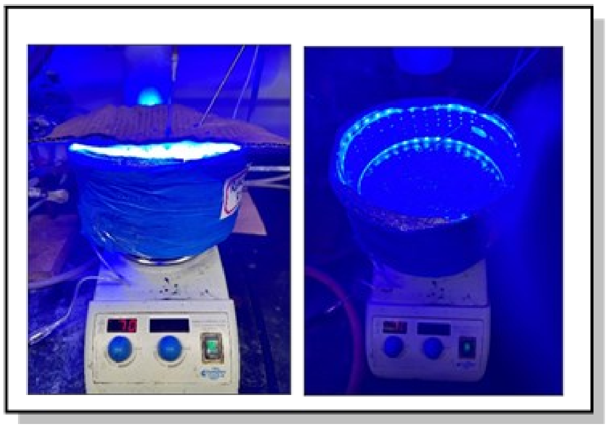

Figure S1. Reaction set up in the light bath.

## Synthesis of substrates:

### General procedure for synthesis of benzylic chlorides 1a-1e

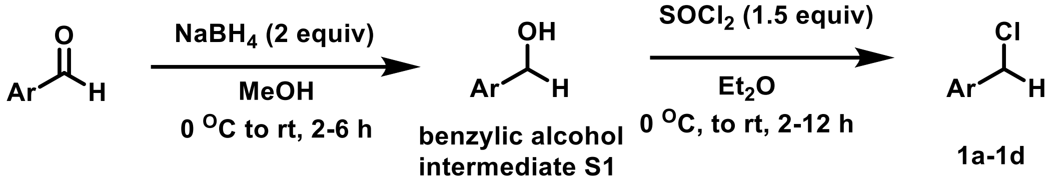


**Step 1**: Under nitrogen atmosphere, benzaldehyde (10 mmol, 1.0 equiv) and anhydrous MeOH (30 mL) were added to a 50 mL three-necked round-bottomed flask at 0 °C. $NaBH_4$ (20 mmol, 0.75 g, 2.0 equiv) was slowly added to the resultant mixture. The reaction was then stirred for an additional 30 min at this temperature, the ice bath was removed, and the temperature was allowed to warm to room temperature and stirred for 2-6 h. After the rection was complete as indicated by TLC, the reaction was quenched with water (30 mL) and extracted by ethyl acetate (30 mL x 3) and washed with brine (30 mL x 3), dried over $MgSO_4$ and filtered. The solvent was then evaporated to afford intermediate **S1** which was pure enough and used to the next step without further purification.

**Step 2**: Intermediate **S1** (10 mmol, 1.0 equiv) was diluted with $Et_2O$ (10 mL) and transferred to a 30 mL reaction tube. After cooling to 0 °C, thionyl chloride (15 mmol, 1.5 equiv) was added dropwise over 5 min. The reaction mixture was stirred at room temperature for 2-14 h until the complete consumption of **S1** as indicated by TLC. Upon completion, the resulting solution was poured onto ice and washed with saturated aq $NaHCO_3$ (2 x 20 mL), extracted with $Et_2O$ (2 x 30 mL), and dried over $MgSO_4$ and filtered. The solvent was removed, and the crude mixture was purified by silica gel flash column with n-hexane as eluent to give benzylic chlorides **1a-1e**.

### 1-(1-chloroethyl)-4-(trifluoromethyl)benzene (1a)

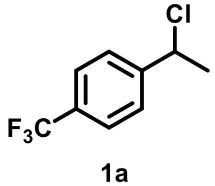

1a

Compound **1a** was prepared from 1-(4-(trifluoromethyl)phenyl)ethan-1-one (1.88 g, 1.5 mL, 10 mmol) according to the general procedure. The crude was purified via automated flash chromatography using EtOAc in hexanes (0% to 100%) with product eluting at 3% on a 24 g silica column. Colorless oil, 1.77 g, 82% yield, 2 steps. 1H NMR (400 MHz, $CDCl_3$) δ 7.63 (d, J = 8.2 Hz, 2H), 7.55 (d, J = 8.2 Hz, 2H), 5.12 (q, J = 6.8 Hz, 1H), 1.86 (d, J = 6.9 Hz, 3H). NMR chemical shifts match with the literature value.[1]

**2-(1-chloroethyl)naphthalene (1b)**

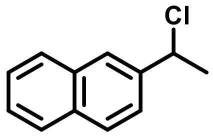


**1b**

Compound **1b** was prepared from 1-(naphthalen-2-yl)ethan-1-one (1.7 g, 10 mmol) according to the general procedure. The crude was purified via automated flash chromatography using EtOAc in hexanes (0% to 100%) with product eluting at 3% on a 24 g silica column. Colorless oil, 1.4 g, 77% yield, 2 steps. 1H NMR (400 MHz, $CDCl_3$) δ 7.94 – 7.79 (m, 4H), 7.58 (dd, J = 8.5, 1.9 Hz, 1H), 7.51 – 7.46 (m, 2H), 5.28 (q, J = 6.8 Hz, 1H), 1.95 (d, J = 6.8 Hz, 3H). NMR chemical shifts match with the literature value.[2]

**1-(1-chloroethyl)-2-methylbenzene (1c)**

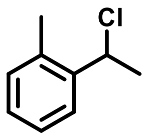


**1c**

Compound **1c** was prepared from 1-(o-tolyl)ethan-1-one (1.3 g, 1.3 mL, 10 mmol) according to the general procedure. The crude was purified via automated flash chromatography using EtOAc in hexanes (0% to 100%) with product eluting at 3% on a 24 g silica column. Colorless oil, 1.3 g, 89% yield, 2 steps. $^{1}$H NMR (400 MHz, $CDCl_3$) δ 7.32 (d, J = 8.0 Hz, 2H), 7.17 (d, J = 7.8 Hz, 2H), 5.09 (q, J = 6.9 Hz, 1H), 2.36 (s, 3H), 1.85 (dd, J = 6.8, 0.7 Hz, 3H). NMR chemical shifts match with the literature value.[3]

**1-(2-chloropropan-2-yl)-3-methylbenzene (1d)**

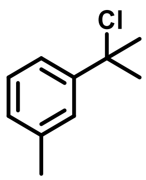


**1d**

Compound **1d** was prepared from 2-(m-tolyl)propan-2-ol (1.5 g, 1.5 mL, 10 mmol) according to the general procedure. Colorless oil, 1.7 g, quantitative yield (no further purification was necessary) , 2 steps. 1H NMR (400 MHz, $CDCl_3$) δ 7.47 – 7.38 (m, 2H), 7.27 (t, J = 7.7 Hz, 1H), 7.12 (d, J = 7.5 Hz, 1H), 2.41 (s, 4H), 2.02 (d, J = 1.0 Hz, 6H). NMR chemical shifts match with the literature value.[4]

**(1-chloroethyl)benzene (1e)**

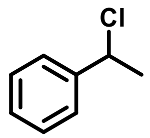


**1e**

Compound **1e** was prepared from 1-phenylethan-1-ol (1.5 g, 1.5 mL, 10 mmol) according to the general procedure. The crude was purified via automated flash chromatography using EtOAc in hexanes (0% to 100%) with product eluting at 2.5% on a 24 g silica column. Colorless oil, 1.1 g, 82%, 2 steps. 1H NMR (400 MHz, $CDCl_3$) δ 7.54 – 7.43 (m, 2H), 7.42 – 7.31 (m, 3H), 5.13 (q, J = 6.8 Hz, 1H), 1.89 (d, J = 6.9 Hz, 3H). NMR chemical shifts match with the literature value.[3]

**Synthesis of benzyl methanesulfonate (1f)**

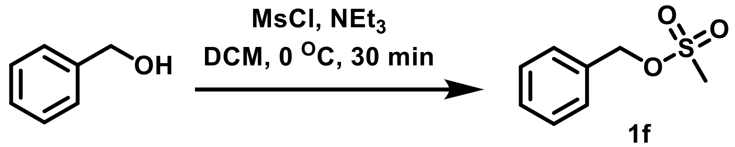


Benzyl alcohol (1g, 1mL, 10 mmol) was treated following a previously published procedure[5] to yield **1f** as colorless liquid (967mg, 52% yield). The crude was purified via automated flash chromatography using EtOAc in hexanes (0% to 100%) with product eluting at 20% on a 24 g silica column. $^1$H NMR (400 MHz, $CDCl_3$) δ 7.47 – 7.33 (m, 5H), 5.26 – 5.20 (m, 2H), 2.92 – 2.87 (m, 3H). NMR chemical shifts match with the literature value.[6]

## Reaction Optimization

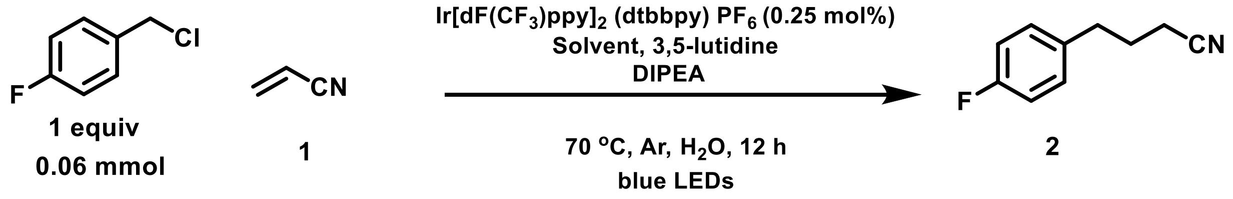


| Entry | 1/ (equiv) | Lutidine/ (equiv) | Solvent | DIPEA/ (equiv) | $H_2O$ (equiv) | Yield of 2%[a] |
|---|---|---|---|---|---|---|
| 1 | 2.5 | 0.7 | MeCN | 1 | 300 | 46 |
| 2 | 2.5 | 0.7 | MeCN | 2 | 300 | 77 |
| 3 | 2 | 0.7 | MeCN | 2.5 | 300 | |
| 4 | 2 | 0.7 | MeCN | 3 | 300 | 74 |
| 5 | 2 | 0.7 | MeCN | 3.5 | 300 | 71 |
| 6 | 3 | 0.7 | MeCN | 3.5 | 300 | 84 |
| 7 | 2 | 0.7 | MeCN | 4 | 300 | 77 |
| 8 | 2.5 | 0.7 | MeCN | 3.5 | 300 | 80 |
| 9 | 4 | 0.7 | MeCN | 3.5 | 50 | 31 |
| 10 | 4 | 0.7 | MeCN | 3.5 | 100 | 34 |
| 11 | 4 | 0.7 | MeCN | 3.5 | 150 | 46 |
| 12 | 4 | 0.7 | MeCN | 3.5 | 200 | 67 |
| 13 | 4 | 0.7 | MeCN | 4 | 250 | 71 |
| 14 | 4 | 0.7 | MeCN | 4 | 300 | 75 |
| 15 | 4 | 0.7 | MeCN | 4 | 350 | 67 |
| 16 | 1 | 0.7 | MeCN | 3.5 | 300 | 65 |
| 17 | 4 | 0.7 | MeCN | 3.5 | 300 | 66 |
| 18 | 3 | 0.2 | MeCN | 3.5 | 300 | 73 |
| 19 | 3 | 0.4 | MeCN | 3.5 | 300 | 78 |
| 20 | 3 | 0.8 | MeCN | 3.5 | 300 | 70 |
| 21 | 3 | 1 | MeCN | 3.5 | 300 | 68 |
| 22 | 2 | 0.7 | DMF | 3.5 | 300 | 53 |
| 23 | 2 | 0.7 | EtOH | 3.5 | 300 | 55 |
| 25 | 2 | 0.7 | Acetone | 3.5 | 300 | 68 |
| 24 | 2 | 0.7 | DMSO | 3.5 | 300 | 54 |
| 26 | 2 | 0.7 | DMA | 3.5 | 300 | 61 |
| 27 | 2 | 0.7 | NMP | 3.5 | 300 | 0 |

**Table S1.** Optimization of reaction conditions

**Table S2. Probing the impact of lutidine methyl group location on the reaction outcome**

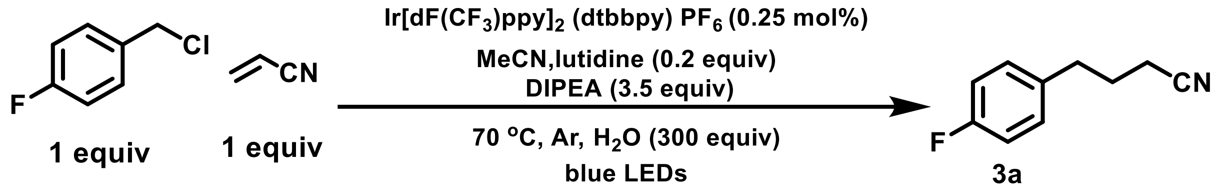


| entry | lutidine | time | conversion of halide | Yield (3a)* |
|---|---|---|---|---|
| 1 | 3,5-lutidine | 12 h | 100% | 54% |
| 2 | 2,6-lutidine | 16 h | 99% | 8% |

*Conversions and yields were determined by 19F NMR using 4-fluoroaniline as an internal standard.

It was observed that 3,5-lutidine significantly increased the reaction yield in comparison to 2,6-lutidine.

## Mechanistic Experiments

Is the proposed intermediate a competent reaction partner?

**Reaction with *N*-para-F-benzyl lutidinium salt**

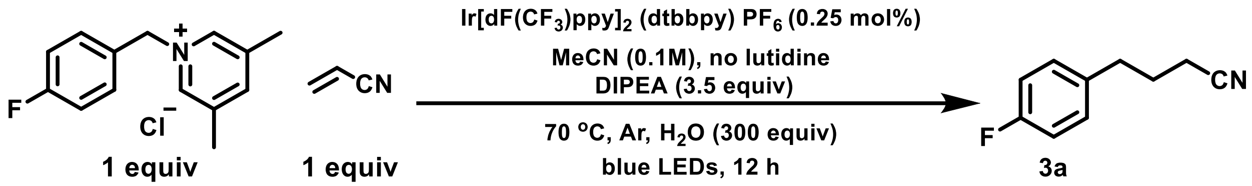


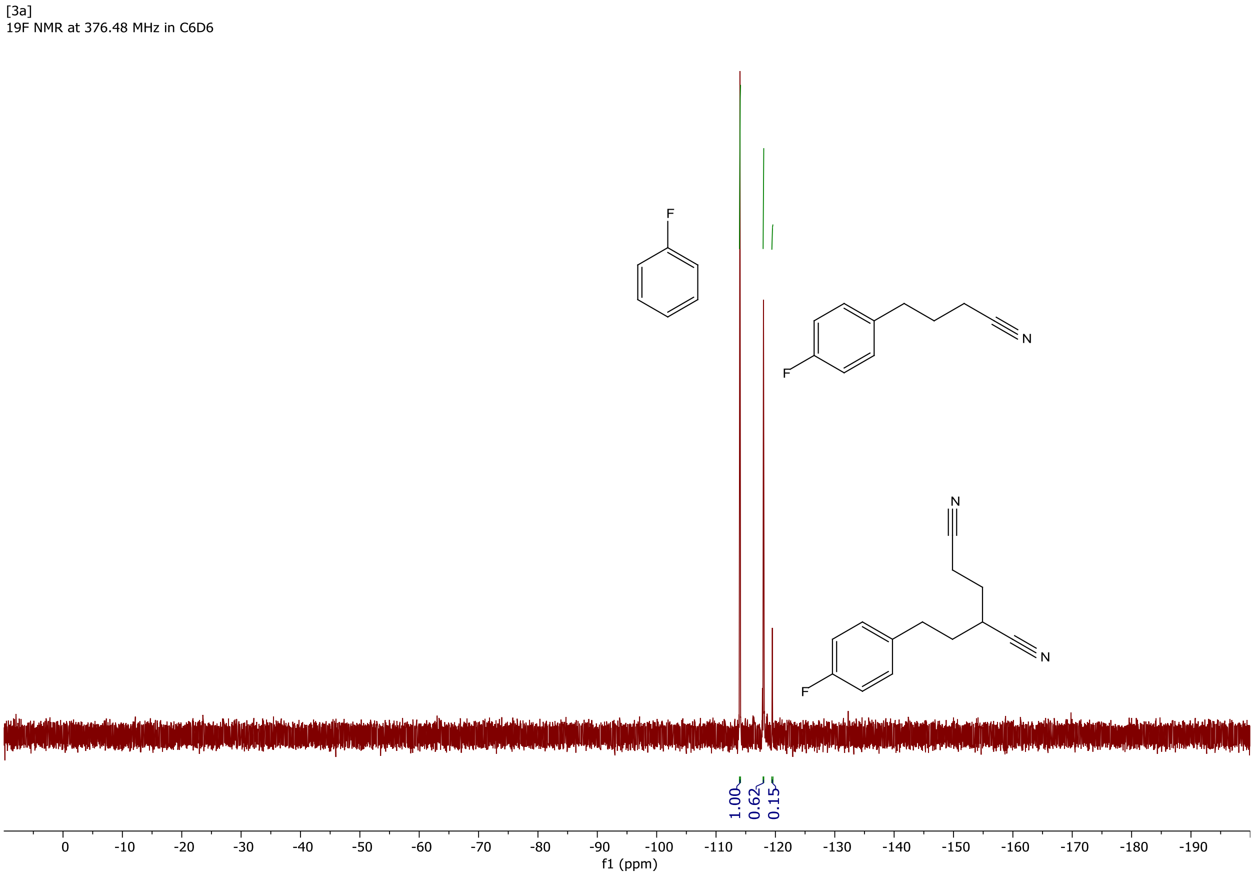


*Conversions were determined by 19F NMR using fluorobenzene as an internal standard.

Using partially optimized conditions, the halide was substituted with lutidine to form the corresponding lutidinium salt. The use of this salt, rather than the benzyl chloride and free lutidine, resulted in a 62% NMR yield, suggesting that the lutidinium salt is capable of forming the product.

## Tracking the reaction as a function of time

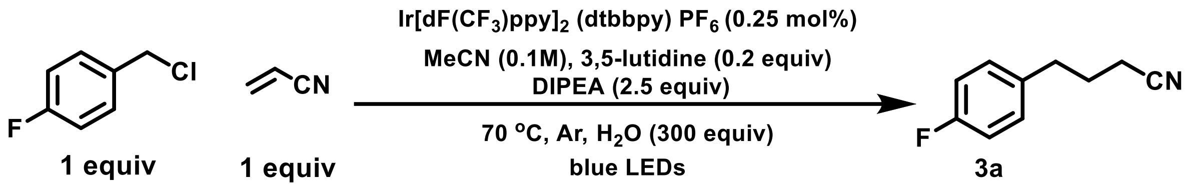


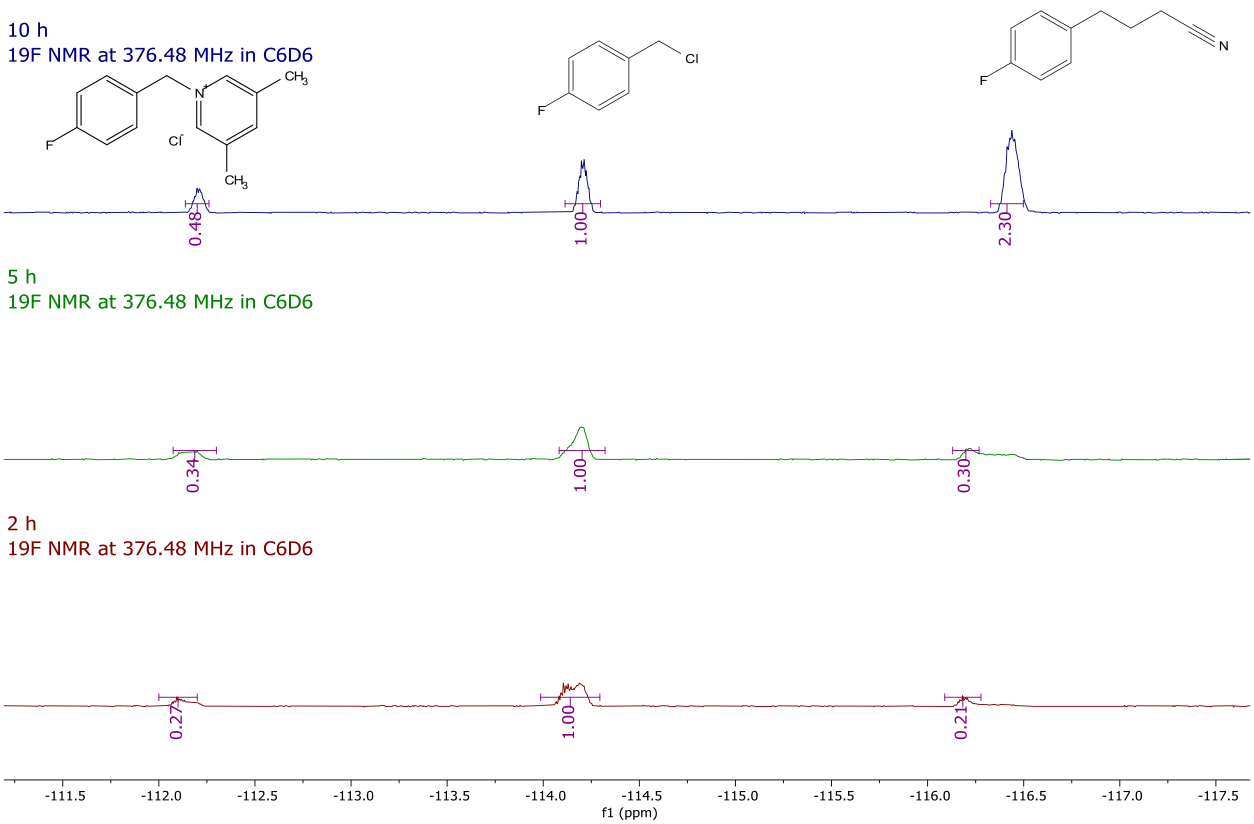


Monitoring the reaction by 19F NMR revealed a significant buildup of lutidinium salt occurred within a span of 2 hours. This is consistent with an RDS after salt formation.

## Reaction without lutidine

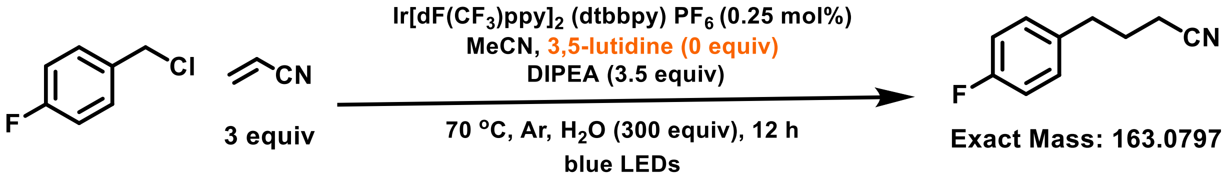


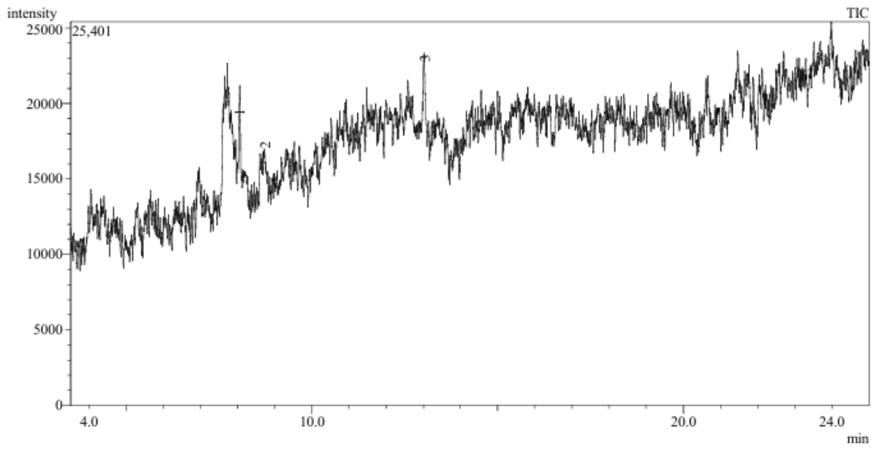


Peak Report TIC

| Peak# | R.Time | I.Time | F.Time | Area | Area% | Height | Height% | A/H | Mark | Name |
|---|---|---|---|---|---|---|---|---|---|---|
| | | | | 0 | 0.00 | 0 | 0.00 | | | |

<< Target >>
Line#:2 R.Time:8.725(Scan#:1046) MassPeaks:341
RawMode:Single 8.725(1046) BasePeak:180.20(543)
BG Mode:None Group 1 - Event 1 Scan

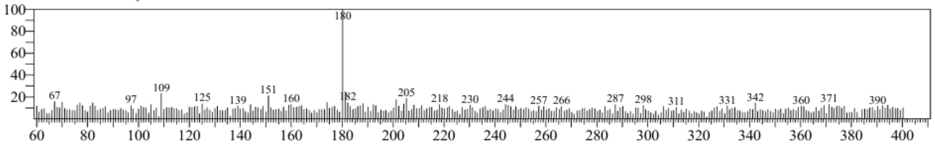


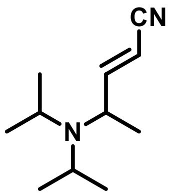


**Exact Mass: 180.1626**

We performed the reaction in the absence of lutidine using the benzyl chloride (3a). We observed no hydrodehalogenation. We did, however, observe the addition of DIPEA to the acrylonitrile as described above.

**Deuterium incorporation experiments**

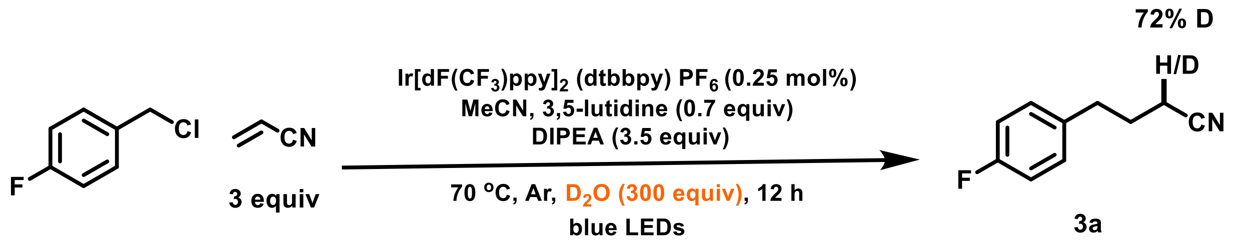


The reaction was set up according to the general procedure B except that 300 equiv of D2o were included instead of H2O. The reaction was monitored by 19F NMR. After the complete consumption of starting material, the volatiles (MeCN, acrylonitrile and some DIPEA) were removed via rotovap and the residue was dissolved in ethyl acetate (6 mL) and washed with 1 M aqueous HCl solution (3 x 2 mL) and brine solution (2 mL). The organic layer was separated and dried over anhydrous $MgSO_4$ and concentrated in vacuo.

The deuterium incorporation experiments revealed that $D_2O$ resulted in partial incorporation of the deuterium (72%) at the alpha position to the nitrile product. 1H NMR of the crude mixture of 3a-H/D is shown below. The difference in the integration of the highlighted signal (2.32 ppm 2H) is due to the incorporation of deuterium in the alpha position of 3a-D.

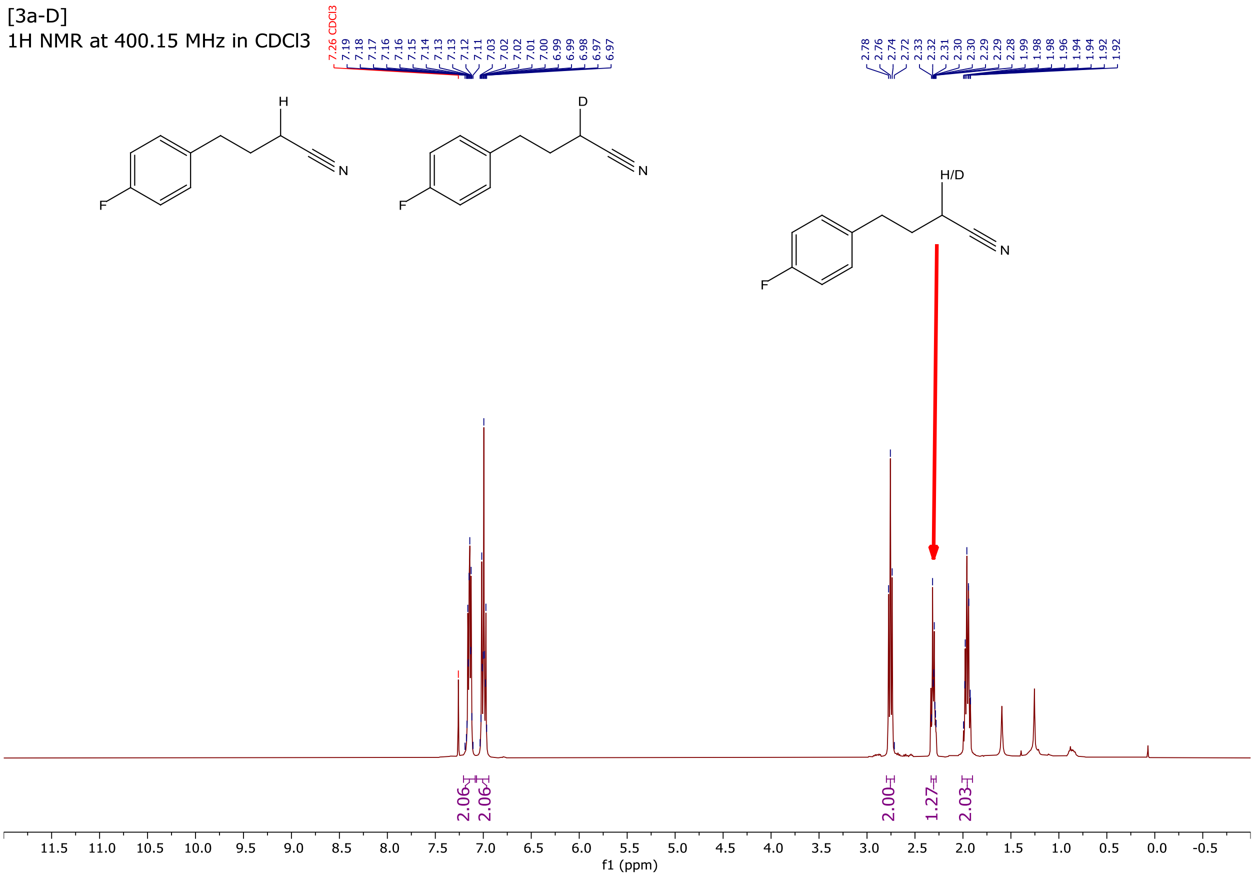


## Electrochemical measurements

Cyclic voltammograms and electrochemical potentials were obtained with a Pine WaveNow Potentiostat. All the samples were prepared with 0.3 mmol of substrate in 30 mL of 0.1 M tetra-n butylammonium hexafluorophosphate in dry, degassed acetonitrile. Measurements were taken using a glassy carbon working electrode (3 mm diameter, 0.07 $cm^2$), a platinum wire counter electrode, and a 4.0 M KCl Ag/AgCl reference electrode. The working electrode was polished using a 0.05 µm alumina slurry on micro cloth before use. All measurements were performed at room temperature. Data was analyzed using AFTERMATH software by identifying the maximum current (Cp) and determining the potential (Ep/2) at half of the maximum current (Cp/2). The obtained value was referenced to Ag/AgCl and converted to SCE by subtracting 0.03

V. The initial potential was set to 1000 mV, direction of initial scan (oxidative), and scan rate was 100 mV/s.

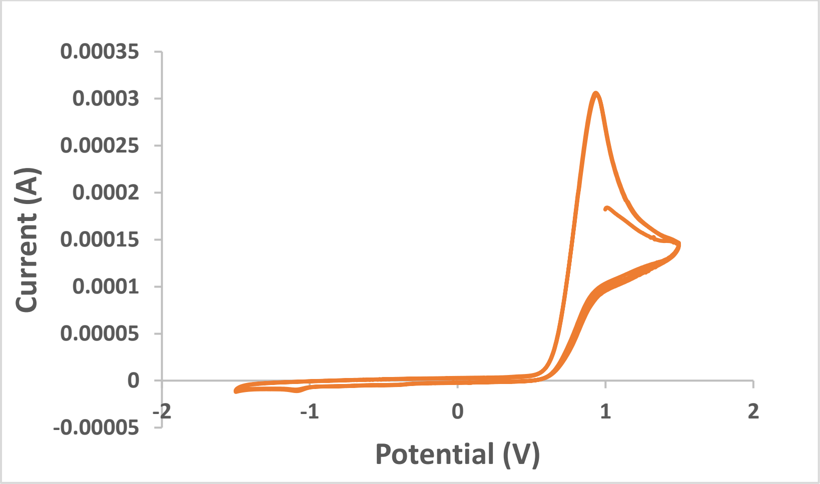


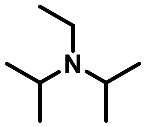

**E$_{1/2}$ = 0.61 V (0.58 V vs. SCE)**

**Figure S2.** Cyclic voltammogram of DIPEA using the IUPAC plotting convention. Starting point: +1.0 V, oxidative scan. Working electrode - glassy carbon, a platinum wire counter electrode, and a 4.0 M KCl Ag/AgCl reference electrode.The sample was prepared with 0.3 mmol of substrate in 30 mL of 0.1 M tetra-n butylammonium hexafluorophosphate in dry, degassed acetonitrile.

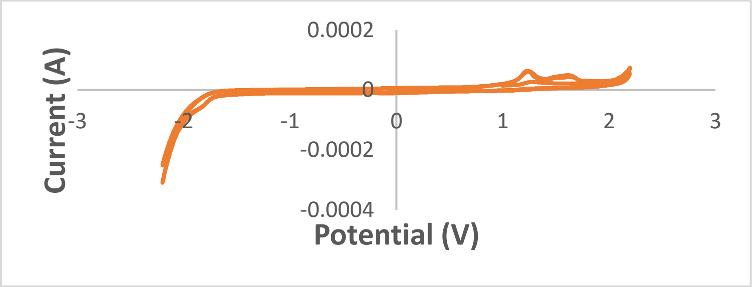


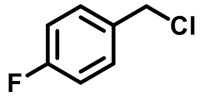

**E$_{1/2}$ = -2 V (-1.97 V vs SCE)**

**Figure S3.** Cyclic voltammogram of para-F-benzylchloride using the IUPAC plotting convention. Starting point: +1.0 V, oxidative scan. Working electrode - glassy carbon, a platinum wire counter electrode, and a 4.0 M KCl Ag/AgCl reference electrode. The sample was prepared with 0.3 mmol of substrate in 30 mL of 0.1 M tetra-n butylammonium hexafluorophosphate in dry, degassed acetonitrile.

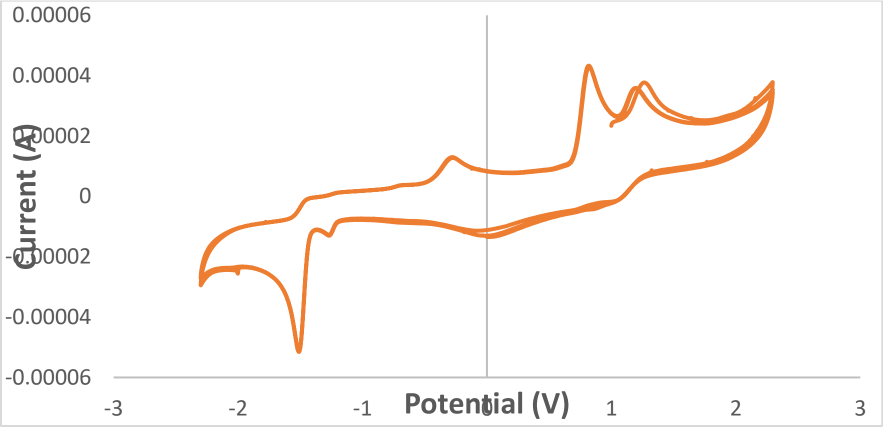


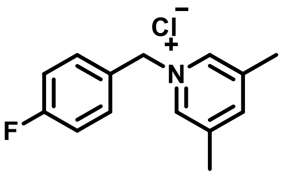


**$E_{1/2}$ = -1.46 V (-1.43 V vs SCE)**

**Figure S4.** Cyclic voltammogram of *N*-para-F-benzyl lutidinium salt. Starting point: +1.0 V, oxidative scan. Working electrode - glassy carbon, a platinum wire counter electrode, and a 4.0 M KCl Ag/AgCl reference electrode. The sample was prepared with 0.3 mmol of substrate in 30 mL of 0.1 M tetra-n butylammonium hexafluorophosphate in dry, degassed acetonitrile.

**Photocatalytic reactions:**

**General procedure B**

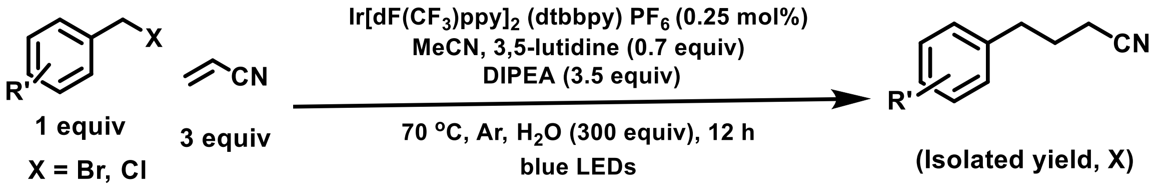


Three NMR tubes fitted with a rubber septum were charged with a solution of [Ir(2′,4′-dF-5-$CF_3$-ppy)$_2$(4,4′- dtbbpy)]$PF_6$ (0.25 mM, 0.5 mL in MeCN), benzyl bromide/ chloride (0.18 mmol, 1 equiv), 3,5-lutidine (0.126 mmol, 13.38 mg, 14.3 µL, 0.7 equiv). DIPEA (0.63 mmol, 81.2 mg 109.5 µL, 3.5 equiv), DI water (54 mmol, 972 mg, 972 µL, 300 equiv) and acrylonitrile (0.54 mmol, 28.6 mg, 36 µL, 3 equiv) were also added. Then the reaction mixture was degassed via Ar bubbling for 10 min and then left under positive Ar pressure by removing the exit needle. The tube was placed in a light bath (description above) which was maintained at 70 °C. The reaction was monitored by 1H or 19F NMR. After the complete consumption of benzyl bromide/ chloride, the volatiles (MeCN, acrylonitrile and some DIPEA) were removed via rotovap and the residue was dissolved in ethyl acetate (12 mL) and washed with 1 M aqueous HCl solution (3 x 4 mL) and brine solution (6 mL). The organic layer was dried over anhydrous $MgSO_4$ and filtered. The crude product was concentrated in vacuo and purified via normal phase chromatography.

4-(4-fluorophenyl)butanenitrile **(3a)**

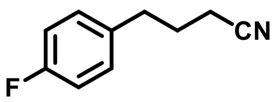


The general procedure A was followed using 1-(chloromethyl)-4-fluorobenzene (0.18 mmol, 26 mg, 21.6 µL, 1 equiv), 3,5-lutidine (0.126 mmol, 13.38 mg, 14.3 µL, 0.7 equiv), DIPEA (0.63 mmol, 81.2 mg 109.5 µL, 3.5 equiv), DI water (54 mmol, 972 mg, 972 µL, 300 equiv), acrylonitrile (0.54 mmol, 28.6 mg, 36 µL, 3 equiv) and 0.5 mL of stock solution of [Ir(2′,4′-dF-5-$CF_3$-ppy)$_2$(4,4′-dtbbpy)]$PF_6$ (0.25 mM), in MeCN. After the completion of the reaction in 12 h, the crude was purified via automated flash chromatography using EtOAc in hexanes (0% to 100%) with product eluting at 3.5% on a 4 g silica column to afford **3a** in 83% yield (32 mg, 0.19 mmol) as colorless oil. NMR chemical shifts match with the literature values. $^1$H NMR (400 MHz, $CDCl_3$) δ 7.14 (ddd, $J$ = 8.2, 5.2, 2.4 Hz, 2H), 6.99 (td, $J$ = 8.7, 2.4 Hz, 2H), 2.76 (td, $J$ = 7.5, 2.4 Hz, 2H), 2.32 (td, $J$ = 7.1, 2.5 Hz, 2H), 1.96 (pd, $J$ = 7.3, 2.4 Hz, 2H). $^{13}$C NMR (101 MHz, $CDCl_3$) δ 135.3, 129.9 (d, $J$ = 8.0

Hz), 119.4, 115.6, 115.4, 33.6, 27.0, 16.4. $^{19}F$ NMR (376 MHz, $CDCl_3$) δ -116.5 – -116.6 (m). NMR chemical shifts match with the literature value.[7]

4-(2-fluorophenyl)butanenitrile **(3b)**

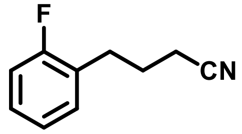

The general procedure A was followed using 1-(chloromethyl)-2-fluorobenzene (0.18 mmol, 26 mg, 21.4 µL, 1 equiv), 3,5-lutidine (0.126 mmol, 13.38 mg, 14.3 µL, 0.7 equiv), DIPEA (0.63 mmol, 81.2 mg 109.5 µL, 3.5 equiv), DI water (54 mmol, 972 mg, 972 µL, 300 equiv), acrylonitrile (0.54 mmol, 28.6 mg, 36 µL, 3 equiv) and 0.5 mL of stock solution of [Ir(2′,4′-dF-5-$CF_3$-ppy)$_2$(4,4′-dtbbpy)]$PF_6$ (0.25 mM), in MeCN. After the completion of the reaction in 12 h, the crude was purified via automated flash chromatography using EtOAc in hexanes (0% to 100%) with product eluting at 3.2% on a 4 g silica column to afford **3b** in 77% yield (51% yield from premade collidinium salts[7]) (22 mg, 0.14 mmol) as yellow oil. $^1H$ NMR (400 MHz, $CDCl_3$) δ 7.21 (qd, $J$ = 6.5, 2.0 Hz, 2H), 7.14 – 6.99 (m, 2H), 2.82 (t, $J$ = 7.4 Hz, 2H), 2.35 (t, $J$ = 7.2 Hz, 2H), 1.99 (p, $J$ = 7.2 Hz, 2H). 13C NMR (201 MHz, CDCl3) δ 160.7, 130.9 (d, J = 4.5 Hz), 128.6 (d, J = 7.6 Hz), 126.8 (d, J = 15.0 Hz), 124.4 (d, J = 4.4 Hz), 119.5, 115.6 (d, J = 22.3 Hz), 28.2, 25.9, 16.7. $^{19}F$ NMR (376 MHz, $CDCl_3$) δ -118.5 – -118.6 (m). NMR chemical shifts match with the literature value.[7]

4-(p-tolyl)butanenitrile **(3c)**

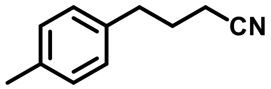

The general procedure A was followed using 1-(chloromethyl)-4-methylbenzene (0.18 mmol, 25.3 mg, 23.8 µL, 1 equiv), 3,5-lutidine (0.126 mmol, 13.38 mg, 14.3 µL, 0.7 equiv), DIPEA (0.63 mmol, 81.2 mg 109.5 µL, 3.5 equiv), DI water (54 mmol, 972 mg, 972 µL, 300 equiv), acrylonitrile (0.54 mmol, 28.6 mg, 36 µL, 3 equiv) and 0.5 mL of stock solution of [Ir(2′,4′-dF-5-$CF_3$-ppy)$_2$(4,4′-dtbbpy)]$PF_6$ (0.25 mM), in MeCN. After the completion of the reaction in 12 h, the crude was purified via automated flash chromatography using EtOAc in hexanes (0% to 100%) with product eluting at 3% on a 4 g silica column to afford **3c** in 88% yield (80% yield from premade collidinium salts[7]) (24 mg, 0.15 mmol) as colorless oil. $^1H$ NMR (400 MHz, $CDCl_3$) δ 7.12 (d, $J$ = 8.0 Hz, 2H), 7.08 (d, $J$ = 8.1 Hz, 2H), 2.74 (t, $J$ = 7.4 Hz, 2H), 2.34 – 2.28 (m, 5H), 1.96 (p, $J$ = 7.2 Hz, 2H). $^{13}C$ NMR (201 MHz, $CDCl_3$) δ 136.6, 136.1, 129.4, 128.4, 119.6, 34.0, 27.0, 21.0, 16.4. NMR chemical shifts match with the literature value.[7]

4-(thiophen-2-yl)butanenitrile **(3d)**

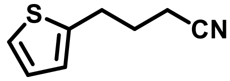

The general procedure A was followed using 1-(chloromethyl)-4-methylbenzene (0.18 mmol, 25.3 mg, 23.8 µL, 1 equiv), 3,5-lutidine (0.126 mmol, 13.38 mg, 14.3

μL, 0.7 equiv), DIPEA (0.63 mmol, 81.2 mg 109.5 μL, 3.5 equiv), DI water (54 mmol, 972 mg, 972 μL, 300 equiv), acrylonitrile (0.54 mmol, 28.6 mg, 36 μL, 3 equiv) and 0.5 mL of stock solution of [Ir(2′,4′-dF-5-$CF_3$-ppy)$_2$(4,4′-dtbbpy)]$PF_6$ (0.25 mM), in MeCN. After the completion of the reaction in 12 h, the crude was purified via automated flash chromatography using EtOAc in hexanes (0% to 100%) with product eluting at 3% on a 4 g silica column to afford **3d** in 78% yield (69% yield from premade collidinium salts[7]) (21 mg, 0.14 mmol) as pale yellow oil. Crude 1H NMR yield of the reaction is 81% with respect to an internal standard (1,3 benzodioxole). $^1$H NMR (400 MHz, $CDCl_3$) δ 7.10 (dd, *J* = 5.1, 1.2 Hz, 1H), 6.87 (dd, *J* = 5.2, 3.4 Hz, 1H), 6.77 (d, *J* = 3.4 Hz, 1H), 2.94 (t, *J* = 7.2 Hz, 2H), 2.31 (t, *J* = 7.1 Hz, 2H), 1.96 (p, *J* = 7.2 Hz, 2H). $^{13}$C NMR (201 MHz, $CDCl_3$) δ 142.1, 127.0, 125.3, 123.9, 119.2, 28.5, 27.3, 16.2. NMR chemical shifts match with the literature value.[7]

4-(3-bromophenyl)butanenitrile **(3e)**

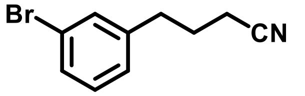


The general procedure A was followed using 1-bromo-3-(bromomethyl)benzene (0.18 mmol, 45 mg, 1 equiv), 3,5-lutidine (0.126 mmol, 13.38 mg, 14.3 μL, 0.7 equiv), DIPEA (0.63 mmol, 81.2 mg 109.5 μL, 3.5 equiv), DI water (54 mmol, 972 mg, 972 μL, 300 equiv), acrylonitrile (0.54 mmol, 28.6 mg, 36 μL, 3 equiv) and 0.5 mL of stock solution of [Ir(2′,4′-dF-5-$CF_3$-ppy)$_2$(4,4′-dtbbpy)]$PF_6$ (0.25 mM), in MeCN. After the completion of the reaction in 12 h, the crude was purified via automated flash chromatography using EtOAc in hexanes (0% to 100%) with product eluting at 3.2% on a 4 g silica column to afford **3e** in 79% yield (71% yield from premade collidinium salts[7]) (32 mg, 0.14 mmol) as colorless oil. $^1$H NMR (400 MHz, $CDCl_3$) δ 7.36 (ddt, *J* = 9.0, 7.1, 3.4 Hz, 2H), 7.18 (t, *J* = 7.7 Hz, 1H), 7.12 (dt, *J* = 7.7, 1.4 Hz, 1H), 2.76 (t, *J* = 7.5 Hz, 2H), 2.33 (t, *J* = 7.0 Hz, 2H), 1.98 (p, *J* = 7.2 Hz, 2H). $^{13}$C NMR (201 MHz, $CDCl_3$) δ 142.0, 131.5, 130.3, 129.7, 127.2, 122.8, 119.2, 34.0, 26.7, 16.4. NMR chemical shifts match with the literature value.[7]

4-(4-nitrophenyl)butanenitrile **(3f)**

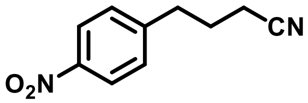


The general procedure A was followed using 1-(chloromethyl)-4-nitrobenzene (0.18 mmol, 30.9 mg, 1 equiv), 3,5-lutidine (0.126 mmol, 13.38 mg, 14.3 μL, 0.7 equiv), DIPEA (0.63 mmol, 81.2 mg 109.5 μL, 3.5 equiv), DI water (54 mmol, 972 mg, 972 μL, 300 equiv), acrylonitrile (0.54 mmol, 28.6 mg, 36 μL, 3 equiv) and 0.5 mL of stock solution of [Ir(2′,4′-dF-5-$CF_3$-ppy)$_2$(4,4′-dtbbpy)]$PF_6$ (0.25 mM), in MeCN. After the completion of the reaction in 12 h, the crude was purified via automated flash chromatography using EtOAc in hexanes (0% to 100%) with product eluting at 4% on a 4 g silica column to afford **3f** in 92% yield (31 mg, 0.16 mmol) as yellow oil. $^1$H NMR (400 MHz, $CDCl_3$) δ 8.20 – 8.16 (m, 2H), 7.42 – 7.33 (m, 2H), 2.91 (t, *J* = 7.7 Hz, 2H), 2.38 (t, *J* = 7.0 Hz, 2H), 2.03 (p, *J* = 7.1 Hz, 2H). $^{13}$C NMR (201 MHz, $CDCl_3$) δ 147.5, 146.9, 129.3, 124.0, 118.9,

34.2, 26.4, 16.6. NMR chemical shifts match with the literature value.[8] HRMS (ESI) m/z: [M+H] + calcd for $C_{10}H_{10}O_2N_3H$ 191.0815; found 191.0818.

4-phenylbutanenitrile **(3g)**

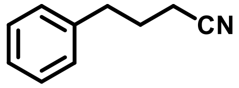


The general procedure A was followed using (chloromethyl)benzene (0.18 mmol, 32.7 mg, 20.7 µL, 1 equiv), 3,5-lutidine (0.126 mmol, 13.38 mg, 14.3 µL, 0.7 equiv), DIPEA (0.63 mmol, 81.2 mg 109.5 µL, 3.5 equiv), DI water (54 mmol, 972 mg, 972 µL, 300 equiv), acrylonitrile (0.54 mmol, 28.6 mg, 36 µL, 3 equiv) and 0.5 mL of stock solution of [Ir(2′,4′-dF-5-$CF_3$-ppy)$_2$(4,4′-dtbbpy)]$PF_6$ (0.25 mM), in MeCN. After the completion of the reaction in 12 h, the crude was purified via automated flash chromatography using EtOAc in hexanes (0% to 100%) with product eluting at 2.3% on a 4 g silica column to afford **3g** in 96% yield (81% yield from premade collidinium salts[7]) (25 mg, 0.17 mmol) as pale yellow oil. $^1$H NMR (400 MHz, $CDCl_3$) δ 7.28 (tt, $J$ = 7.7, 1.4 Hz, 2H), 7.25 – 7.17 (m, 1H), 7.16 (tt, $J$ = 5.7, 1.3 Hz, 2H), 2.75 (t, $J$ = 7.4 Hz, 2H), 2.28 (t, $J$ = 7.1 Hz, 2H), 1.95 (p, $J$ = 7.2 Hz, 2H). $^{13}$C NMR (201 MHz, $CDCl_3$) δ 139.7, 128.7, 128.5, 126.5, 119.5, 34.4, 26.9, 16.4. NMR chemical shifts match with the literature value.[7]

4-(3-cyanopropyl)benzonitrile **(3h)**

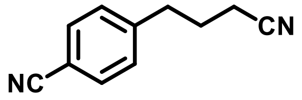


The general procedure A was followed using 4-(chloromethyl)benzonitrile (0.18 mmol, 27.3 mg, 1 equiv), 3,5-lutidine (0.126 mmol, 13.38 mg, 14.3 µL, 0.7 equiv), DIPEA (0.63 mmol, 81.2 mg 109.5 µL, 3.5 equiv), DI water (54 mmol, 972 mg, 972 µL, 300 equiv), acrylonitrile (0.54 mmol, 28.6 mg, 36 µL, 3 equiv) and 0.5 mL of stock solution of [Ir(2′,4′-dF-5-$CF_3$-ppy)$_2$(4,4′-dtbbpy)]$PF_6$ (0.25 mM), in MeCN. After the completion of the reaction in 12 h, the crude was purified via automated flash chromatography using EtOAc in hexanes (0% to 100%) with product eluting at 3.2% on a 4 g silica column to afford **3h** in 93% yield (70% yield from premade collidinium salts[7]) (28 mg, 0.16 mmol) as colorless oil. $^1$H NMR (400 MHz, $CDCl_3$) δ 7.65 – 7.57 (m, 2H), 7.37 – 7.27 (m, 2H), 2.90 – 2.81 (m, 2H), 2.36 (t, $J$ = 7.0 Hz, 2H), 2.00 (dq, $J$ = 8.9, 7.0 Hz, 2H). $^{13}$C NMR (201 MHz, $CDCl_3$) δ 145.3, 132.5, 129.3, 119.0, 118.8, 110.7, 34.5, 26.4, 16.5. NMR chemical shifts match with the literature value.[7]

4-(4-methoxyphenyl)butanenitrile **(3i)**

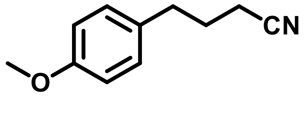


The general procedure A was followed using 1-(chloromethyl)-4-methoxybenzene (0.18 mmol, 28.2 mg, 24.4 µL, 1 equiv), 3,5-lutidine (0.126 mmol, 13.38 mg, 14.3 µL, 0.7 equiv), DIPEA (0.63 mmol, 81.2 mg 109.5 µL, 3.5 equiv), DI water (54 mmol, 972 mg, 972 µL, 300 equiv), acrylonitrile (0.54 mmol, 28.6 mg, 36 µL, 3 equiv) and 0.5 mL of stock solution of [Ir(2′,4′-dF-5-$CF_3$-ppy)$_2$(4,4′-

dtbbpy)]$PF_6$ (0.25 mM), in MeCN. After the completion of the reaction in 12 h, the crude was purified via automated flash chromatography using EtOAc in hexanes (0% to 100%) with product eluting at 3% on a 4 g silica column to afford **3i** in 88% yield (79% yield from premade collidinium salts[7]) (27 mg, 0.17 mmol) as colorless oil. $^1$H NMR (400 MHz, $CDCl_3$) δ 7.06 – 6.99 (m, 2H), 6.82 – 6.74 (m, 2H), 3.72 (s, 3H), 2.65 (t, *J* = 7.4 Hz, 2H), 2.23 (d, *J* = 14.2 Hz, 1H), 1.87 (p, *J* = 7.2 Hz, 2H). $^{13}$C NMR (201 MHz, $CDCl_3$) δ 158.3, 131.7, 129.4, 119.6, 114.1, 55.3, 33.5, 27.1, 16.3. NMR chemical shifts match with the literature value.[7]

methyl 4-(3-cyanopropyl)benzoate **(3j)**

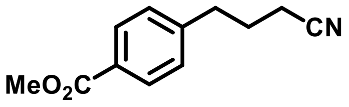


The general procedure A was followed using methyl 4-(bromomethyl)benzoate (0.18 mmol, 41.2 mg, 1 equiv), 3,5-lutidine (0.126 mmol, 13.38 mg, 14.3 µL, 0.7 equiv), DIPEA (0.63 mmol, 81.2 mg 109.5 µL, 3.5 equiv), DI water (54 mmol, 972 mg, 972 µL, 300 equiv), acrylonitrile (0.54 mmol, 28.6 mg, 36 µL, 3 equiv) and 0.5 mL of stock solution of [Ir(2′,4′-dF-5-$CF_3$-ppy)$_2$(4,4′-dtbbpy)]$PF_6$ (0.25 mM), in MeCN. After the completion of the reaction in 12 h, the crude was purified via automated flash chromatography using EtOAc in hexanes (0% to 100%) with product eluting at 12% on a 4 g silica column to afford **3j** in 88% yield (71% yield from premade collidinium salts[7]) (31 mg, 0.16 mmol) as pale yellow oil. $^1$H NMR (400 MHz, $CDCl_3$) δ 7.96 – 7.89 (m, 2H), 7.19 (d, *J* = 2.1 Hz, 2H), 3.84 (s, 3H), 2.78 (t, *J* = 7.5 Hz, 2H), 2.38 – 2.22 (m, 3H), 1.94 (p, *J* = 7.2 Hz, 2H). $^{13}$C NMR (201 MHz, $CDCl_3$) δ 166.9, 145.1, 130.0, 128.6, 128.5, 119.2, 52.1, 34.4, 26.6, 16.5. NMR chemical shifts match with the literature value.[7]

4-(naphthalen-2-yl)butanenitrile **(3k)**

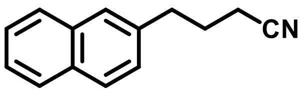


The general procedure A was followed using methyl 2-(bromomethyl)naphthalene (0.18 mmol, 40 mg, 1 equiv), 3,5-lutidine (0.126 mmol, 13.38 mg, 14.3 µL, 0.7 equiv), DIPEA (0.63 mmol, 81.2 mg 109.5 µL, 3.5 equiv), DI water (54 mmol, 972 mg, 972 µL, 300 equiv), acrylonitrile (0.54 mmol, 28.6 mg, 36 µL, 3 equiv) and 0.5 mL of stock solution of [Ir(2′,4′-dF-5-$CF_3$-ppy)$_2$(4,4′-dtbbpy)]$PF_6$ (0.25 mM), in MeCN. After the completion of the reaction in 12 h, the crude was purified via automated flash chromatography using EtOAc in hexanes (0% to 100%) with product eluting at 3% on a 4 g silica column to afford **3k** in 85% yield (78% yield from premade collidinium salts[7]) (30 mg, 0.15 mmol) as a white solid. $^1$H NMR (400 MHz, $CDCl_3$) δ 7.81 (dt, *J* = 7.1, 5.4 Hz, 3H), 7.65 (d, *J* = 1.8 Hz, 1H), 7.53 – 7.40 (m, 2H), 7.32 (dd, *J* = 8.4, 1.8 Hz, 1H), 2.95 (t, *J* = 7.4 Hz, 2H), 2.34 (t, *J* = 7.0 Hz, 2H), 2.08 (p, *J* = 7.2 Hz, 2H). $^{13}$C NMR (201 MHz, $CDCl_3$) δ 137.1, 133.6, 132.3, 128.4, 127.7, 127.5, 126.9, 126.8, 126.3, 125.6, 119.5, 34.5, 26.8, 16.4. NMR chemical shifts match with the literature value.[7]

4-mesitylbutanenitrile **(3l)**

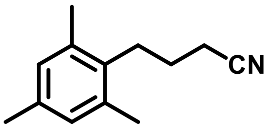


The general procedure A was followed using methyl 2-(chloromethyl)-1,3,5-trimethylbenzene (0.18 mmol, 30 mg, 1 equiv), 3,5-lutidine (0.126 mmol, 13.38 mg, 14.3 µL, 0.7 equiv), DIPEA (0.63 mmol, 81.2 mg 109.5 µL, 3.5 equiv), DI water (54 mmol, 972 mg, 972 µL, 300 equiv), acrylonitrile (0.54 mmol, 28.6 mg, 36 µL, 3 equiv) and 0.5 mL of stock solution of [Ir(2′,4′-dF-5-$CF_3$-ppy)$_2$(4,4′-dtbbpy)]$PF_6$ (0.25 mM), in MeCN. After the completion of the reaction in 12 h, the crude was purified via automated flash chromatography using EtOAc in hexanes (0% to 100%) with product eluting at 2.8% on a 4 g silica column to afford **3l** in 73% yield (65% yield from premade collidinium salts[7]) (25 mg, 0.13 mmol) as colorless oil. $^1$H NMR (400 MHz, $CDCl_3$) δ 6.85 (s, 2H), 2.77 – 2.72 (m, 2H), 2.43 (t, *J* = 7.0 Hz, 2H), 2.29 (s, 6H), 2.25 (s, 3H), 1.86 – 1.75 (m, 2H). $^{13}$C NMR (201 MHz, $CDCl_3$) δ 136.0, 135.8, 133.8, 129.1, 119.6, 28.4, 24.9, 20.8, 19.7, 17.5. NMR chemical shifts match with the literature value.[7]

4-(o-tolyl)butanenitrile **(3m)**

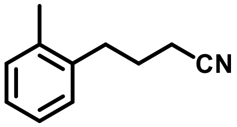


The general procedure A was followed using methyl 1-(chloromethyl)-2-methylbenzene (0.18 mmol, 25.3 mg, 23 µL, 1 equiv), 3,5-lutidine (0.126 mmol, 13.38 mg, 14.3 µL, 0.7 equiv), DIPEA (0.63 mmol, 81.2 mg 109.5 µL, 3.5 equiv), DI water (54 mmol, 972 mg, 972 µL, 300 equiv), acrylonitrile (0.54 mmol, 28.6 mg, 36 µL, 3 equiv) and 0.5 mL of stock solution of [Ir(2′,4′-dF-5-$CF_3$-ppy)$_2$(4,4′-dtbbpy)]$PF_6$ (0.25 mM), in MeCN. After the completion of the reaction in 12 h, the crude was purified via automated flash chromatography using EtOAc in hexanes (0% to 100%) with product eluting at 23% on a 4 g silica column to afford **3m** in 87% yield (25 mg, 0.16 mmol) as colorless oil. $^1$H NMR (400 MHz, $CDCl_3$) δ 7.07 (ddt, *J* = 6.9, 4.3, 2.6 Hz, 4H), 2.70 (dd, *J* = 8.4, 6.9 Hz, 2H), 2.29 (t, *J* = 7.1 Hz, 2H), 2.25 (s, 3H), 1.87 (dq, *J* = 9.1, 7.1 Hz, 2H). $^{13}$C NMR (101 MHz, $CDCl_3$) δ 138.0, 136.0, 130.6, 129.0, 126.7, 126.2, 119.6, 31.9, 25.7, 19.2, 16.8. NMR chemical shifts match with the literature value.[9] HRMS (ESI) m/z: [M+H] + calcd for $C_{11}H_{13}NH$ 160.1121; found 160.1126.

4,4’-(1,4-phenylene)dibutanenitrile **(3n)**

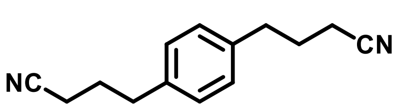


The general procedure A was followed using methyl 1,4-bis(chloromethyl)benzene (0.18 mmol, 31.5 mg, 26.3 µL, 1 equiv), 3,5-lutidine (0.504 mmol, 53.52 mg, 57.2 µL, 2.8 equiv),

DIPEA (1.26 mmol, 162.4 mg 219 µL, 7 equiv), DI water (108 mmol, 1.9 g, 1.9 mL, 600 equiv), acrylonitrile (1.08 mmol, 57.2 mg, 72 µL, 6 equiv) and 0.5 mL of stock solution of [Ir(2′,4′-dF-5-$CF_3$-ppy)$_2$(4,4′-dtbbpy)]$PF_6$ (0.25 mM), in MeCN. After the completion of the reaction in 12 h, the crude was purified via automated flash chromatography using EtOAc in hexanes (0% to 100%) with product eluting at 15% on a 4 g silica column to afford **3n** in 81% yield (31 mg, 0.15 mmol) as colorless oil. $^1$H NMR (400 MHz, $CDCl_3$) δ 7.06 (s, 4H), 2.69 (t, *J* = 7.4 Hz, 4H), 2.25 (t, *J* = 7.1 Hz, 4H), 1.90 (p, *J* = 7.2 Hz, 4H). $^{13}$C NMR (201 MHz, $CDCl_3$) δ 138.0, 128.8, 119.5, 34.0, 26.9, 16.4. HRMS (ESI) m/z: [M+H] + calcd for $C_{14}H_{16}N_2H$ 213.1386; found 213.1394.

4-([1,1'-biphenyl]-4-yl)butanenitrile **(3o)**

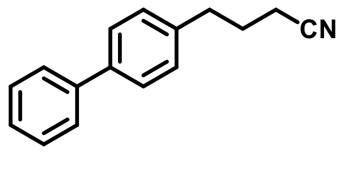


The general procedure A was followed using methyl 4-(bromomethyl)-1,1'-biphenyl (0.18 mmol, 44 mg, 1 equiv), 3,5-lutidine (0.126 mmol, 13.38 mg, 14.3 µL, 0.7 equiv), DIPEA (0.63 mmol, 81.2 mg 109.5 µL, 3.5 equiv), DI water (54 mmol, 972 mg, 972 µL, 300 equiv), acrylonitrile (0.54 mmol, 28.6 mg, 36 µL, 3 equiv) and 0.5 mL of stock solution of [Ir(2′,4′-dF-5-$CF_3$-ppy)$_2$(4,4′-dtbbpy)]$PF_6$ (0.25 mM), in MeCN. After the completion of the reaction in 12 h, the crude was purified via automated flash chromatography using EtOAc in hexanes (0% to 100%) with product eluting at 23% on a 4 g silica column to afford **3o** in 87% yield (25 mg, 0.16 mmol) as colorless oil. $^1$H NMR (400 MHz, $CDCl_3$) δ 7.07 (ddt, *J* = 6.9, 4.3, 2.6 Hz, 4H), 2.70 (dd, *J* = 8.4, 6.9 Hz, 2H), 2.29 (t, *J* = 7.1 Hz, 2H), 2.25 (s, 3H), 1.87 (dq, *J* = 9.1, 7.1 Hz, 2H). $^{13}$C NMR (101 MHz, $CDCl_3$) δ 138.0, 136.0, 130.6, 129.0, 126.7, 126.2, 119.6, 31.9, 25.7, 19.2, 16.8.NMR chemical shifts match with the literature value.[10] HRMS (ESI) m/z: [M+H] + calcd for $C_{16}H_{15}NH$ 222.1277; found 222.1285.

3-(3-oxo-1,3-dihydroisobenzofuran-1-yl)propanenitrile **(3p)**

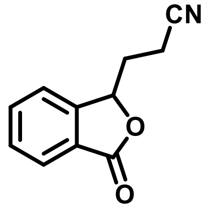


The general procedure A was followed using 3-bromoisobenzofuran-1(3H)-one (0.18 mmol, 38 mg, 1 equiv), 3,5-lutidine (0.126 mmol, 13.38 mg, 14.3 µL, 0.7 equiv), DIPEA (0.63 mmol, 81.2 mg 109.5 µL, 3.5 equiv), DI water (54 mmol, 972 mg, 972 µL, 300 equiv), acrylonitrile (0.54 mmol, 28.6 mg, 36 µL, 3 equiv) and 0.5 mL of stock solution of [Ir(2′,4′-dF-5-$CF_3$-ppy)$_2$(4,4′-dtbbpy)]$PF_6$ (0.25 mM), in MeCN. After the completion of the reaction in 12 h, the crude was purified via automated flash chromatography using EtOAc in hexanes (0% to 100%) with product eluting at 5% on a 4 g silica column to afford **3p** in 72% yield (24 mg, 0.13 mmol) as white solid. $^1$H NMR (400 MHz, $CDCl_3$) δ 7.87 (d, *J* = 7.7 Hz, 1H), 7.67 (td, *J* = 7.5, 1.1 Hz, 1H), 7.53 (t, *J* = 7.5 Hz, 1H), 7.41 (d, *J* = 7.6 Hz, 1H), 5.52 (dd, *J* = 9.1, 2.9 Hz, 1H), 2.65 – 2.56 (m, 1H), 2.56 – 2.49 (m, 1H), 2.48 – 2.36 (m, 2H). $^{13}$C NMR (101 MHz, $CDCl_3$) δ 169.6, 148.0, 134.6, 129.9,

126.2, 125.9, 121.7, 118.4, 78.5, 30.9, 13.6. HRMS (ESI) m/z: [M+H] + calcd for $C_{11}H_9O_2NH$ 188.0706; found 188.0713.

4-phenylnonanenitrile **(3q)**

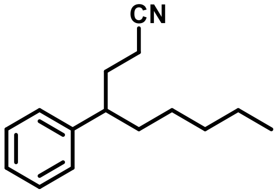


The general procedure A was followed using (1-bromohexyl)benzene (0.18 mmol, 43 mg, 1 equiv), 3,5-lutidine (0.126 mmol, 13.38 mg, 14.3 µL, 0.7 equiv), DIPEA (0.63 mmol, 81.2 mg 109.5 µL, 3.5 equiv), DI water (54 mmol, 972 mg, 972 µL, 300 equiv), acrylonitrile (0.54 mmol, 28.6 mg, 36 µL, 3 equiv) and 0.5 mL of stock solution of [Ir(2′,4′-dF-5-$CF_3$-ppy)$_2$(4,4′-dtbbpy)]$PF_6$ (0.25 mM), in MeCN. After the completion of the reaction in 12 h, the crude was purified via automated flash chromatography using EtOAc in hexanes (0% to 100%) with product eluting at 3.2% on a 4 g silica column to afford **3q** in 95% yield (73 % yield from premade collidinium salts[7]) (51% yield from premade collidinium salts[7]) (37 mg, 0.17 mmol) as pale yellow oil. $^1$H NMR (800 MHz, $CDCl_3$) δ 7.32 (t, $J$ = 7.6 Hz, 2H), 7.25 – 7.21 (m, 1H), 7.16 – 7.13 (m, 2H), 2.65 (tdd, $J$ = 10.7, 7.3, 4.0 Hz, 1H), 2.21 – 2.14 (m, 1H), 2.08 – 1.99 (m, 2H), 1.86 – 1.80 (m, 1H), 1.62 (dddd, $J$ = 13.5, 11.8, 8.8, 5.4 Hz, 2H), 1.29 – 1.17 (m, 5H), 1.16 – 1.08 (m, 1H), 0.83 (t, $J$ = 6.8 Hz, 3H). $^{13}$C NMR (201 MHz, $CDCl_3$) δ 143.1, 128.8, 127.6, 126.8, 119.7, 44.9, 36.5, 32.3, 31.8, 27.1, 22.5, 15.5, 14.0. NMR chemical shifts match with the literature value.[7]

4-(o-tolyl)pentanenitrile **(3r)**

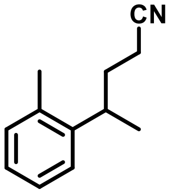


The general procedure A was followed using 1-(1-chloroethyl)-2-methylbenzene (0.18 mmol, 28 mg, 27 µL, 1 equiv), 3,5-lutidine (0.126 mmol, 13.38 mg, 14.3 µL, 0.7 equiv), DIPEA (0.63 mmol, 81.2 mg 109.5 µL, 3.5 equiv), DI water (54 mmol, 972 mg, 972 µL, 300 equiv), acrylonitrile (0.54 mmol, 28.6 mg, 36 µL, 3 equiv) and 0.5 mL of stock solution of [Ir(2′,4′-dF-5-$CF_3$-ppy)$_2$(4,4′-dtbbpy)]$PF_6$ (0.25 mM), in MeCN. After the completion of the reaction in 12 h, the crude was purified via automated flash chromatography using EtOAc in hexanes (0% to 100%) with product eluting at 2.2% on a 4 g silica column to afford **3r** in 79% yield (30mg, 0.14 mmol) as colorless oil. $^1$H NMR (400 MHz, $CDCl_3$) δ 7.06 (d, $J$ = 7.9 Hz, 2H), 7.00 (d, $J$ = 8.1 Hz, 2H), 2.90 – 2.64 (m, 1H), 2.17 – 1.98 (m, 2H), 1.96 – 1.74 (m, 2H), 1.22 (d, $J$ = 6.9 Hz, 3H). $^{13}$C NMR (201 MHz, $CDCl_3$) δ 141.5, 136.3, 129.5, 126.8, 119.7, 38.5, 33.6, 22.1, 21.0, 15.5. NMR chemical shifts match with the literature value.[11] HRMS (ESI) m/z: [M+H] + calcd for $C_{11}H_{13}NH$ 160.1121; found 160.1126.

4-phenylpentanenitrile **(3s)**

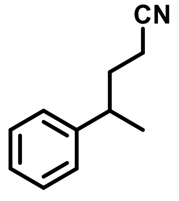

The general procedure A was followed using (1-chloroethyl)benzene (0.18 mmol, 25.3 mg, 24 µL, 1 equiv), 3,5-lutidine (0.126 mmol, 13.38 mg, 14.3 µL, 0.7 equiv), DIPEA (0.63 mmol, 81.2 mg 109.5 µL, 3.5 equiv), DI water (54 mmol, 972 mg, 972 µL, 300 equiv), acrylonitrile (0.54 mmol, 28.6 mg, 36 µL, 3 equiv) and 0.5 mL of stock solution of [Ir(2′,4′-dF-5-$CF_3$-ppy)$_2$(4,4′-dtbbpy)]$PF_6$ (0.25 mM), in MeCN. After the completion of the reaction in 12 h, the crude was purified via automated flash chromatography using EtOAc in hexanes (0% to 100%) with product eluting at 3.3% on a 4 g silica column to afford **3s** in 76% yield (22mg, 0.14 mmol) as an orange oil. $^1$H NMR (400 MHz, $CDCl_3$) δ 7.25 (dd, $J$ = 8.2, 6.8 Hz, 2H), 7.19 – 7.10 (m, 3H), 2.85 – 2.70 (m, 1H), 2.15 (ddd, $J$ = 16.8, 7.7, 5.9 Hz, 1H), 2.04 (dt, $J$ = 16.8, 7.8 Hz, 1H), 1.97 – 1.85 (m, 1H), 1.88 – 1.74 (m, 1H), 1.24 (d, $J$ = 7.0 Hz, 3H). $^{13}$C NMR (201 MHz, $CDCl_3$) δ 144.6, 128.8, 126.9, 126.8, 119.7, 39.0, 33.6, 22.0, 15.5. NMR chemical shifts match with the literature value.[11]

4-(naphthalen-2-yl)pentanenitrile **(3t)**

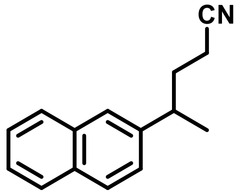

The general procedure A was followed using 2-(1-chloroethyl)naphthalene (0.18 mmol, 34.3 mg, 30 µL, 1 equiv), 3,5-lutidine (0.126 mmol, 13.38 mg, 14.3 µL, 0.7 equiv), DIPEA (0.63 mmol, 81.2 mg 109.5 µL, 3.5 equiv), DI water (54 mmol, 972 mg, 972 µL, 300 equiv), acrylonitrile (0.54 mmol, 28.6 mg, 36 µL, 3 equiv) and 0.5 mL of stock solution of [Ir(2′,4′-dF-5-$CF_3$-ppy)$_2$(4,4′-dtbbpy)]$PF_6$ (0.25 mM), in MeCN. After the completion of the reaction in 12 h, the crude was purified via automated flash chromatography using EtOAc in hexanes (0% to 100%) with product eluting at 5.2% on a 4 g silica column to afford **3t** in 81% yield (30mg, 0.15 mmol) as pale yellow oil. $^1$H NMR (800 MHz, $CDCl_3$) δ 7.89 – 7.81 (m, 3H), 7.67 (d, $J$ = 1.9 Hz, 1H), 7.54 – 7.46 (m, 2H), 7.36 (dd, $J$ = 8.5, 1.8 Hz, 1H), 3.12 – 3.03 (m, 1H), 2.27 (ddd, $J$ = 16.9, 7.5, 5.6 Hz, 1H), 2.15 (dt, $J$ = 16.6, 7.9 Hz, 1H), 2.12 – 1.99 (m, 2H), 1.43 (d, $J$ = 7.0 Hz, 3H). $^{13}$C NMR (201 MHz, $CDCl_3$) δ 141.9, 133.6, 132.5, 128.7, 127.7 (d, $J$ = 9.3 Hz), 126.3, 125.7 (d, $J$ = 8.9 Hz), 124.9, 119.7, 39.1, 33.4, 22.0, 15.5. HRMS (ESI) m/z: [M+H] + calcd for $C_{15}H_{15}NH$ 210.1277; found 210.1284.

4-(4-(trifluoromethyl)phenyl)pentanenitrile **(3u)**

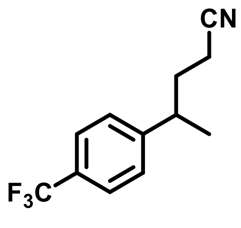

The general procedure A was followed using 1-(1-chloroethyl)-4-(trifluoromethyl)benzene (0.18 mmol, 37.5 mg, 30 µL, 1 equiv), 3,5-lutidine (0.126 mmol, 13.38 mg, 14.3 µL, 0.7 equiv), DIPEA (0.63 mmol, 81.2 mg 109.5 µL, 3.5 equiv), DI water (54 mmol, 972 mg, 972 µL, 300 equiv), acrylonitrile (0.54 mmol, 28.6 mg, 36 µL, 3 equiv) and 0.5 mL of stock solution of [Ir(2′,4′-dF-5-$CF_3$-ppy)$_2$(4,4′-dtbbpy)]$PF_6$ (0.25 mM), in MeCN. After the completion of the reaction in 12 h, the crude was purified via automated flash

chromatography using EtOAc in hexanes (0% to 100%) with product eluting at 2.5% on a 4 g silica column to afford **3u** in 88% yield (36mg, 0.168 mmol) as pale yellow oil. $^{1}$H NMR (400 MHz, $CDCl_3$) δ 7.52 (d, $J$ = 8.1 Hz, 2H), 7.25 (d, $J$ = 8.0 Hz, 2H), 2.89 (dp, $J$ = 9.6, 6.8 Hz, 1H), 2.19 (ddd, $J$ = 16.8, 7.3, 5.9 Hz, 1H), 2.05 (dt, $J$ = 16.8, 7.7 Hz, 1H), 1.93 (dtd, $J$ = 13.6, 7.6, 5.7 Hz, 1H), 1.83 (dddd, $J$ = 13.6, 9.3, 7.4, 5.9 Hz, 1H), 1.26 (d, $J$ = 6.9 Hz, 3H). $^{13}$C NMR (201 MHz, $CDCl_3$) δ 148.7, 129.6 – 128.8 (m), 127.3, 125.8 (q, $J$ = 3.7 Hz), 126.4 – 121.8 (m), 119.2, 38.8, 33.3, 21.7, 15.5. $^{19}$F NMR (376 MHz, $CDCl_3$) δ -62.5. NMR chemical shifts match with the literature value.[12]

4-methyl-4-(m-tolyl)pentanenitrile **(3v)**

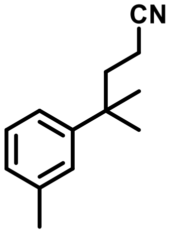


The general procedure A was followed using 1-(2-chloropropan-2-yl)-3-methylbenzene (0.18 mmol, 30.3 mg, 30 μL, 1 equiv), 3,5-lutidine (0.126 mmol, 13.38 mg, 14.3 μL, 0.7 equiv), DIPEA (0.63 mmol, 81.2 mg 109.5 μL, 3.5 equiv), DI water (54 mmol, 972 mg, 972 μL, 300 equiv), acrylonitrile (0.54 mmol, 28.6 mg, 36 μL, 3 equiv) and 0.5 mL of stock solution of [Ir(2′,4′-dF-5-$CF_3$-ppy)$_2$(4,4′-dtbbpy)]$PF_6$ (0.25 mM), in MeCN. After the completion of the reaction in 12 h, the crude was purified via automated flash chromatography using EtOAc in hexanes (0% to 100%) with product eluting at 3.5% on a 4 g silica column to afford **3v** in 36% yield (12mg, 0.06 mmol) as colorless oil. $^{1}$H NMR (400 MHz, $CDCl_3$) δ 7.18 – 7.12 (m, 1H), 7.05 – 6.94 (m, 3H), 2.29 (s, 3H), 1.94 (s, 4H), 1.27 (s, 6H). $^{13}$C NMR (101 MHz, $CDCl_3$) δ 146.4, 138.1, 128.5, 127.1, 126.4, 122.7, 120.3, 39.8, 37.5, 28.5, 21.7, 13.0. HRMS (ESI) m/z: [M+H] + calcd for $C_{13}H_{17}NH$ 188.1434; found 188.1438.

benzyl methyl glutarate **(3w)**

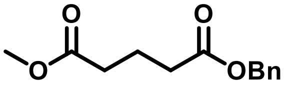


The general procedure A was followed using methyl 2-bromoacetate (0.18 mmol, 28 mg, 17 μL, 1 equiv), 3,5-lutidine (0.126 mmol, 13.38 mg, 14.3 μL, 0.7 equiv), DIPEA (0.63 mmol, 81.2 mg 109.5 μL, 3.5 equiv), DI water (54 mmol, 972 mg, 972 μL, 300 equiv), benzyl acrylate (0.54 mmol, 87 mg, 54 μL, 3 equiv) and 0.5 mL of stock solution of [Ir(2′,4′-dF-5-$CF_3$-ppy)$_2$(4,4′-dtbbpy)]$PF_6$ (0.25 mM), in MeCN. After the completion of the reaction in 12 h, the crude was purified via Prep TLC using EtOAc: hexanes (2:8) to afford **3w** in 72% yield (31mg, 13.1 mmol) as colorless oil. $^{1}$H NMR (400 MHz, $CDCl_3$) δ 7.42 – 7.29 (m, 5H), 5.12 (s, 2H), 3.67 (s, 3H), 2.41 (dt, $J$ = 19.7, 7.4 Hz, 4H), 1.98 (p, $J$ = 7.3 Hz, 2H). $^{13}$C NMR (101 MHz, $CDCl_3$) δ 173.4, 172.8, 135.9, 128.6, 128.3, 128.2, 66.3, 51.6, 33.3, 33.1, 20.1. HRMS (ESI) m/z: [M+H] + calcd for $C_{13}H_{16}O_4H$ 237.1121; found 237.1127.

Photocatalytic reaction in larger scale (1 mmol)

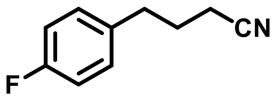


A 18×150 mm borosilicate tube fitted with a rubber septum was charged with a solution of [Ir(2′,4′-dF-5-$CF_3$-ppy)$_2$(4,4′-dtbbpy)]$PF_6$ (0.25 mM, 12 mL in MeCN), 1-(chloromethyl)-4-fluorobenzene (1 mmol, 144.5 mg, 119 µL, 1 equiv), 3,5-lutidine (0.7 mmol, 75 mg, 79 µL, 0.7 equiv), DIPEA (3.5 mmol, 452 mg 600 µL, 3.5 equiv), DI water (300 mmol, 5400 mg, 5400 µL, 300 equiv), acrylonitrile (3 mmol, 159 mg, 196 µL, 3 equiv). Then the reaction mixture was degassed via Ar bubbling for 30 min and then left under positive Ar pressure by removing the exit needle. The tube was placed in a light bath (description above) which was maintained at 70 °C. The reaction was monitored by 19F NMR. After the complete consumption of starting material (1h), the volatiles (MeCN, acrylonitrile and some DIPEA) were removed via rotovap and the residue was dissolved in ethyl acetate (40 mL) and washed with 1 M aqueous HCl solution (3 x 20 mL) and brine solution (20 mL). The organic layer was dried over anhydrous $MgSO_4$ and filtered. The crude product was concentrated in vacuo and purified via normal phase chromatography using EtOAc in hexanes (0% to 100%) with product eluting at 4.2% on a 24 g silica column to afford **3a** in 76% (124 mg, 0.76 mmol) as an oil.

## References


(1) Wang, F.; Nishimoto, Y.; Yasuda, M. Indium-Catalyzed Formal Carbon–Halogen Bond Insertion: Synthesis of α-Halo-α,α-Disubstituted Esters from Benzylic Halides and Diazo Esters. *Org. Lett.* **2022**, *24*, 1706–1710.

(2) Zhang, Q.; Wang, X.; Qian, Q.; Gong, H. Nickel-Catalyzed Reductive Cross-Coupling of Benzyl Halides with Aryl Halides. *Synthesis* **2016**, *48*, 2829–2836.

(3) Liang, S.; Hammond, G. B.; Xu, B. Metal-Free Regioselective Hydrochlorination of Unactivated Alkenes *via* a Combined Acid Catalytic System. *Green Chem.* **2018**, *20*, 680–684.

(4) Reetz, M. T.; Westermann, J.; Steinbach, R. Chemoselective and Position Specific Methylation of *Tert* -Alkyl Halides with Methyltitanium( IV ) Chlorides. *Angew. Chem. Int. Ed. Engl.* **1980**, *19*, 901–902.

(5) Altamura, M.; Perrotta, E. An Efficient Synthesis of 2-(Halogenomethyl)Penems. *J. Org. Chem.* **1993**, *58*, 272–274.

(6) Atack, T. C.; Lecker, R. M.; Cook, S. P. Iron-Catalyzed Borylation of Alkyl Electrophiles. *J. Am. Chem. Soc.* **2014**, *136*, 9521–9523.

(7) Rathnayake, M. D.; Weaver, J. D. Coupling Photocatalysis and Substitution Chemistry to Expand and Normalize Redox-Active Halides. *Org. Lett.* **2021**, *23*, 2036–2041.

(8) Sánchez, I. H.; Aguilar, M. A. Synthesis of 4-Arylbutanenitriles. *Synthesis* **1981**, *1981,* 55–56..

(9) Suga, T.; Shimazu, S.; Ukaji, Y. Low-Valent Titanium-Mediated Radical Conjugate Addition Using Benzyl Alcohols as Benzyl Radical Sources. *Org. Lett.* **2018**, *20*, 5389–5392.

(10) Chen, Q.; You, J.; Tian, T.; Li, Z.; Kashihara, M.; Mori, H.; Nishihara, Y. Nickel-Catalyzed Decarbonylative Reductive Alkylation of Aroyl Fluorides with Alkyl Bromides. *Org. Lett.* **2022**, *24*, 9259–9263.

(11) Mori, S.; Saito, S. C(Sp $^{3}$ )–H Bond Functionalization with Styrenes *via* Hydrogen-Atom Transfer to an Aqueous Hydroxyl Radical under Photocatalysis. *Green Chem.* **2021**, *23*, 3575–3580.

(12) Green, S. A.; Matos, J. L. M.; Yagi, A.; Shenvi, R. A. Branch-Selective Hydroarylation: Iodoarene–Olefin Cross-Coupling. *J. Am. Chem. Soc.* **2016**, *138*, 12779–12782.

[1a]
1H NMR at 400.15 MHz in CDCl3

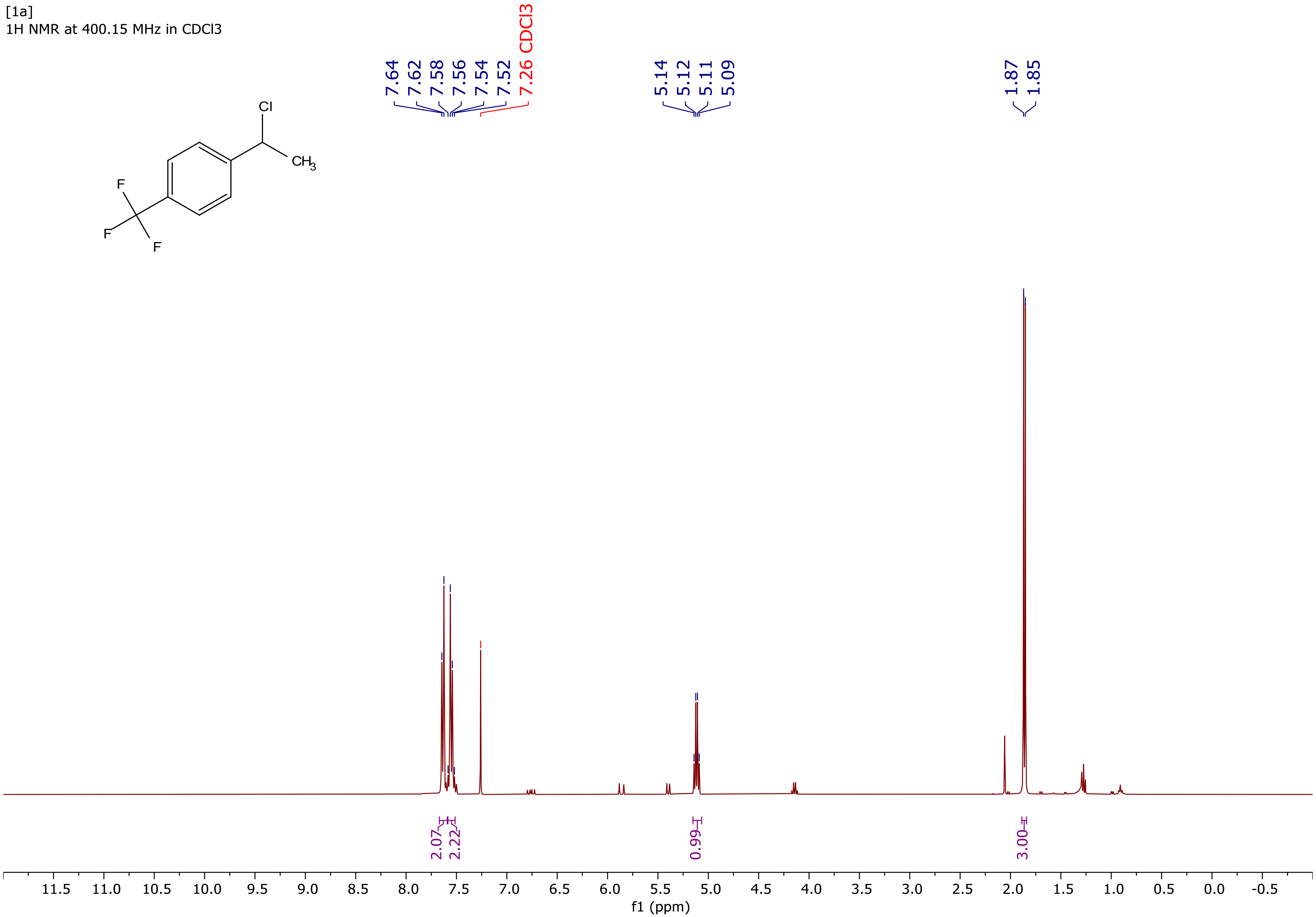

[1b]
1H NMR at 400.15 MHz in CDCl3

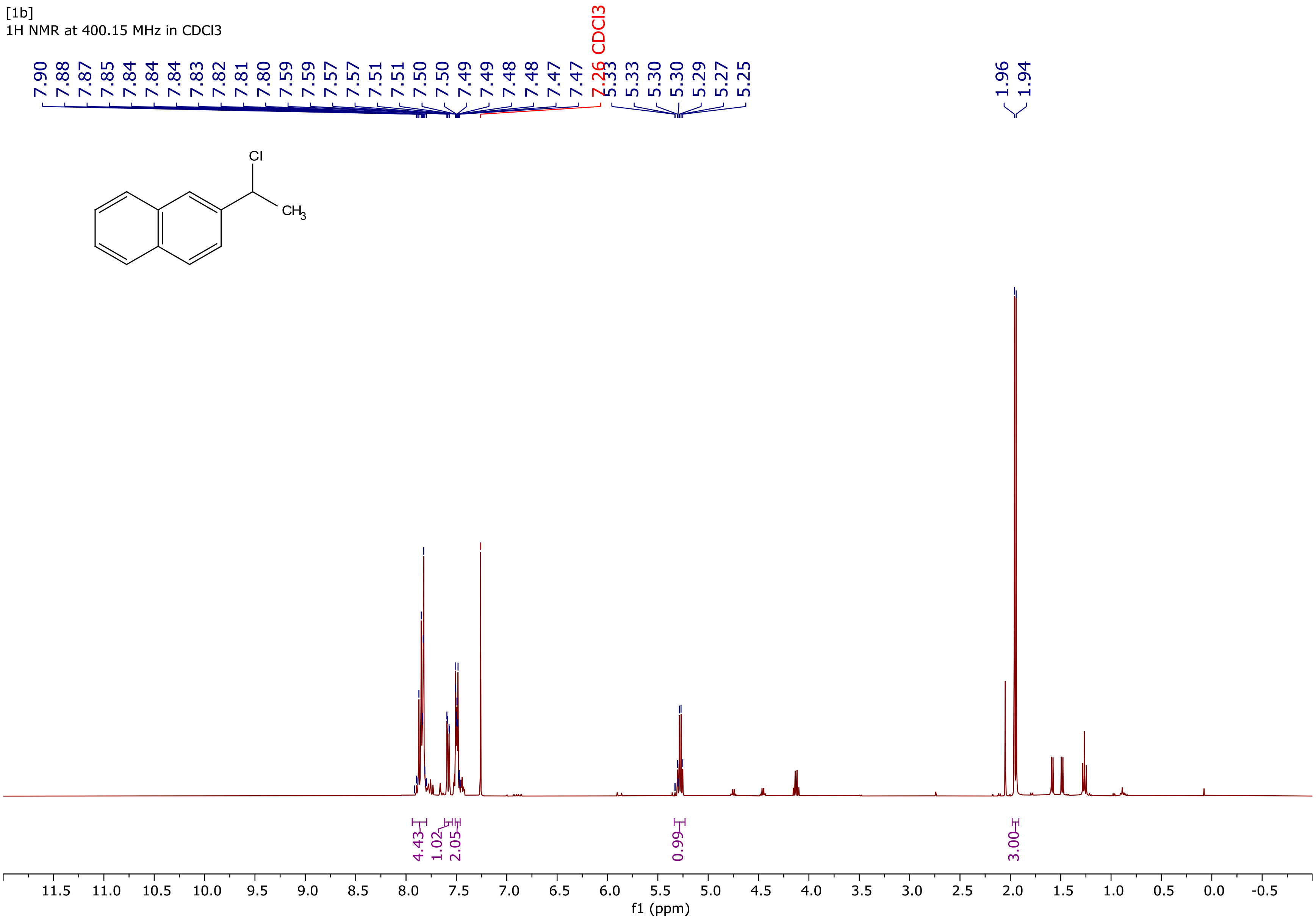

[1c]
1H NMR at 400.15 MHz in CDCl3

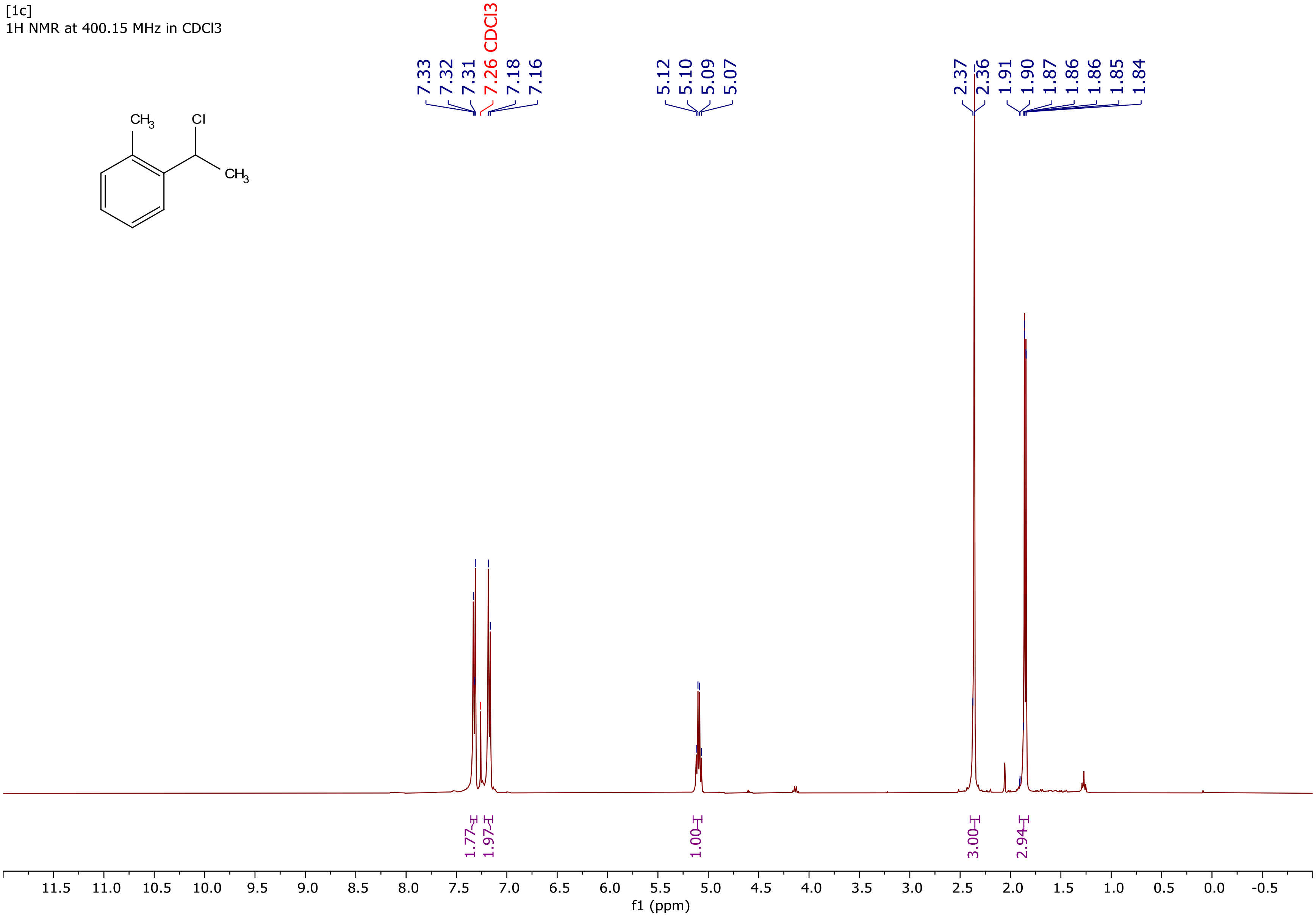

[1d]
1H NMR at 400.15 MHz in CDCl3

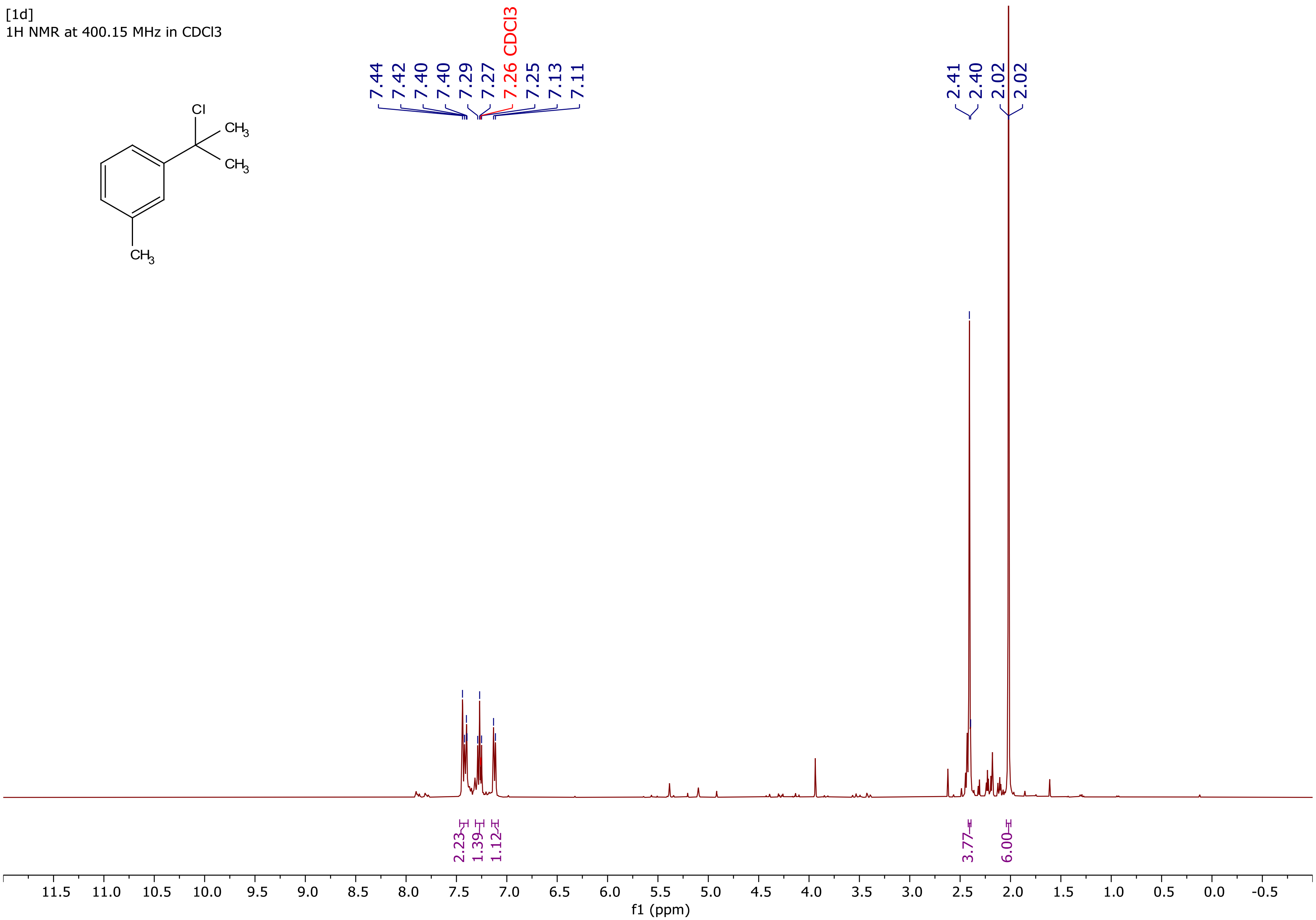

[1e]
1H NMR at 400.15 MHz in CDCl3

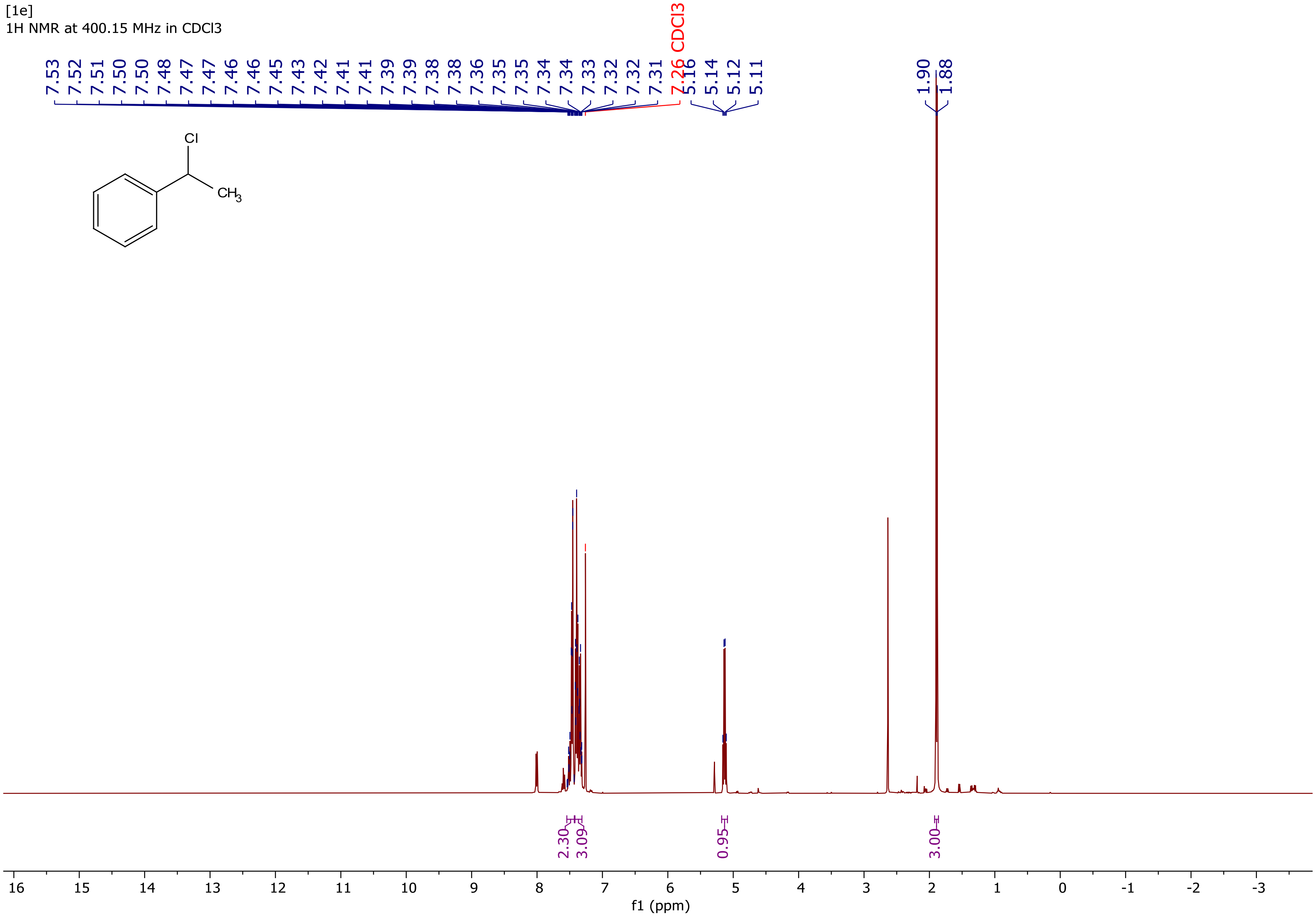

[1f]
1H NMR at 400.15 MHz in CDCl3

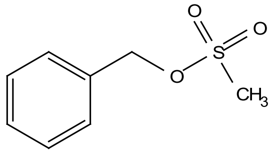

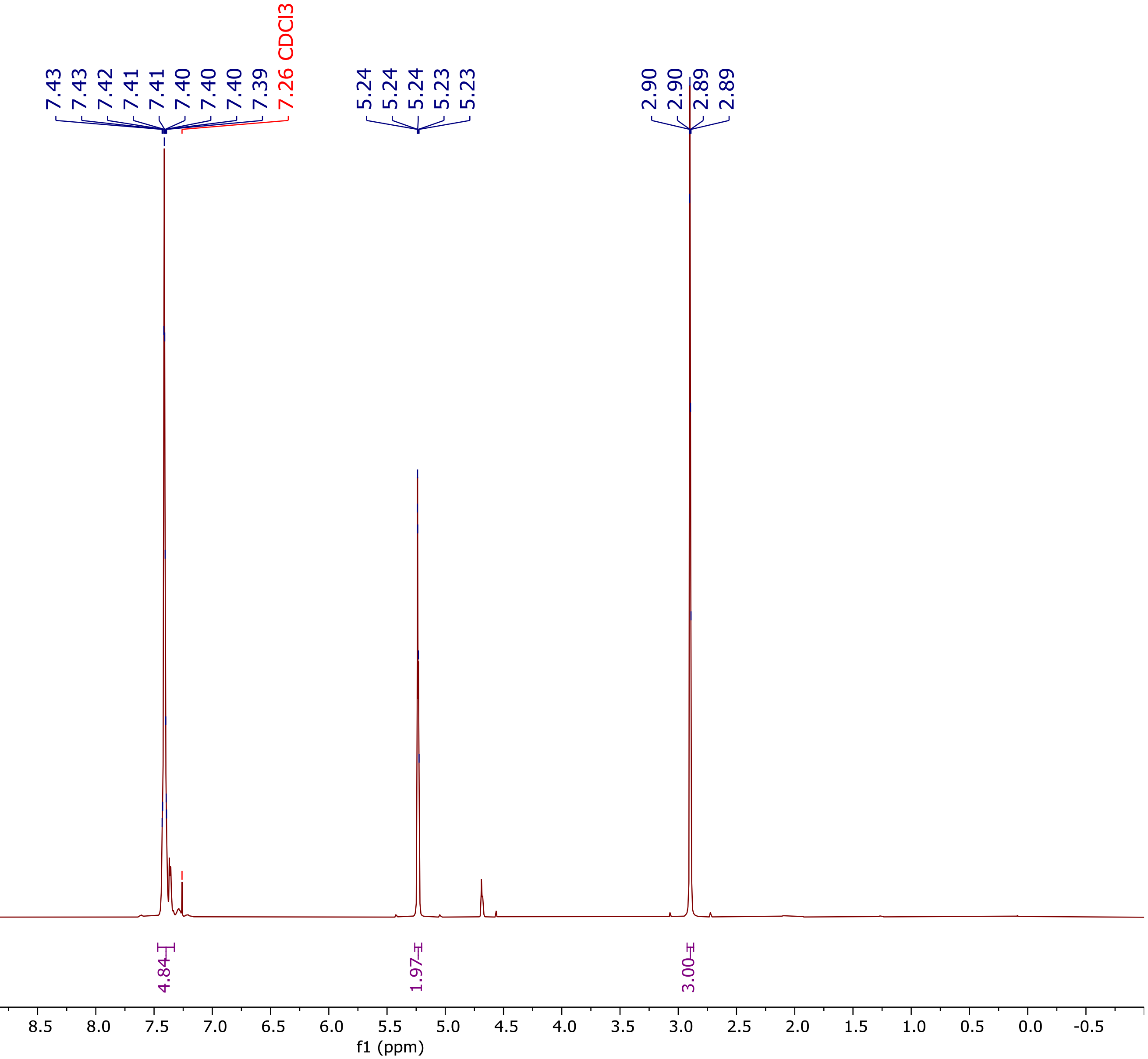

[3a]
1H NMR at 400.15 MHz in CDCl3

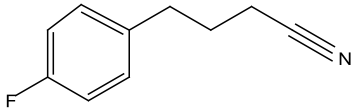

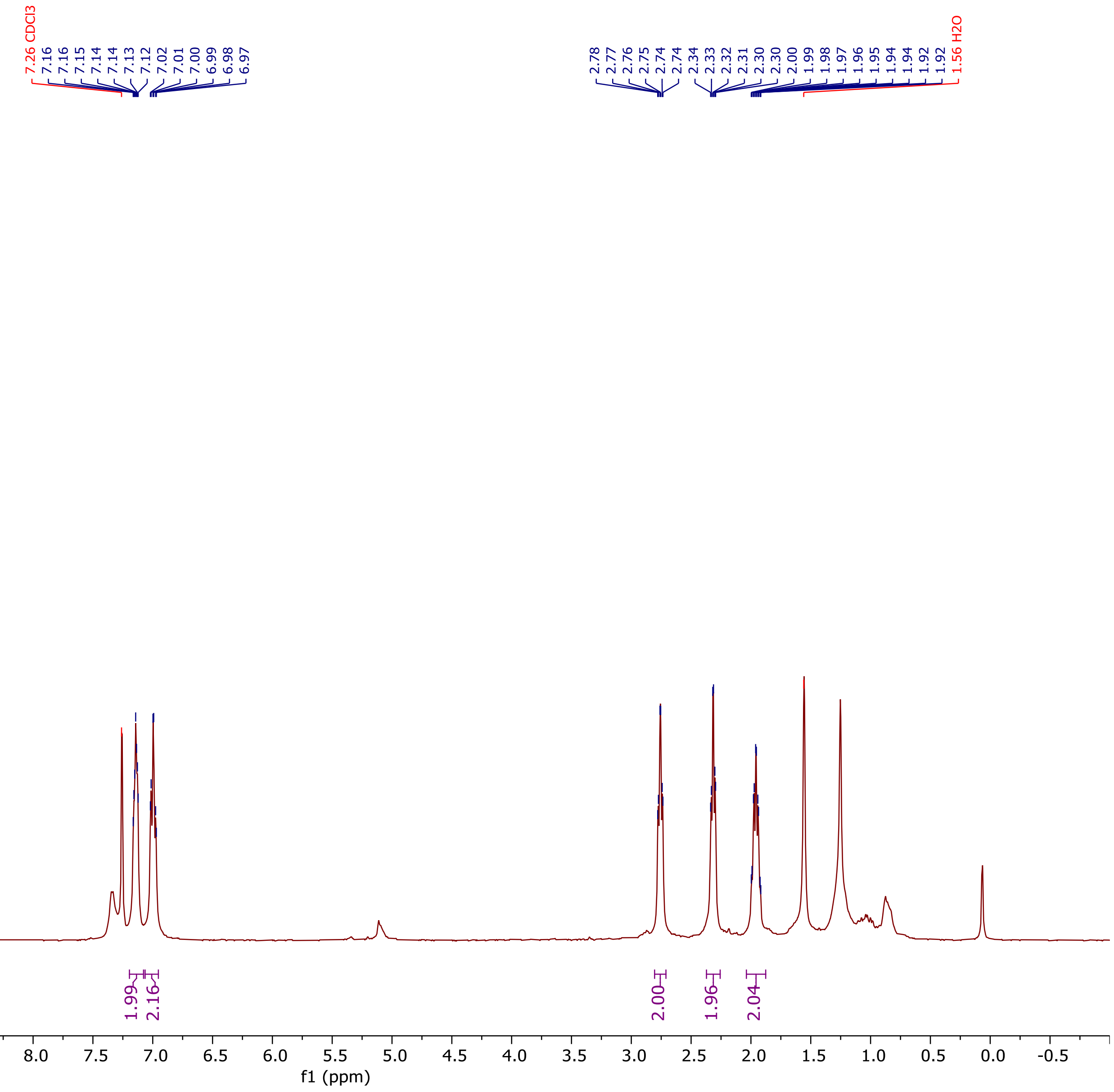

[3a]
13C NMR at 201.27 MHz in CDCl3

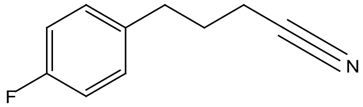

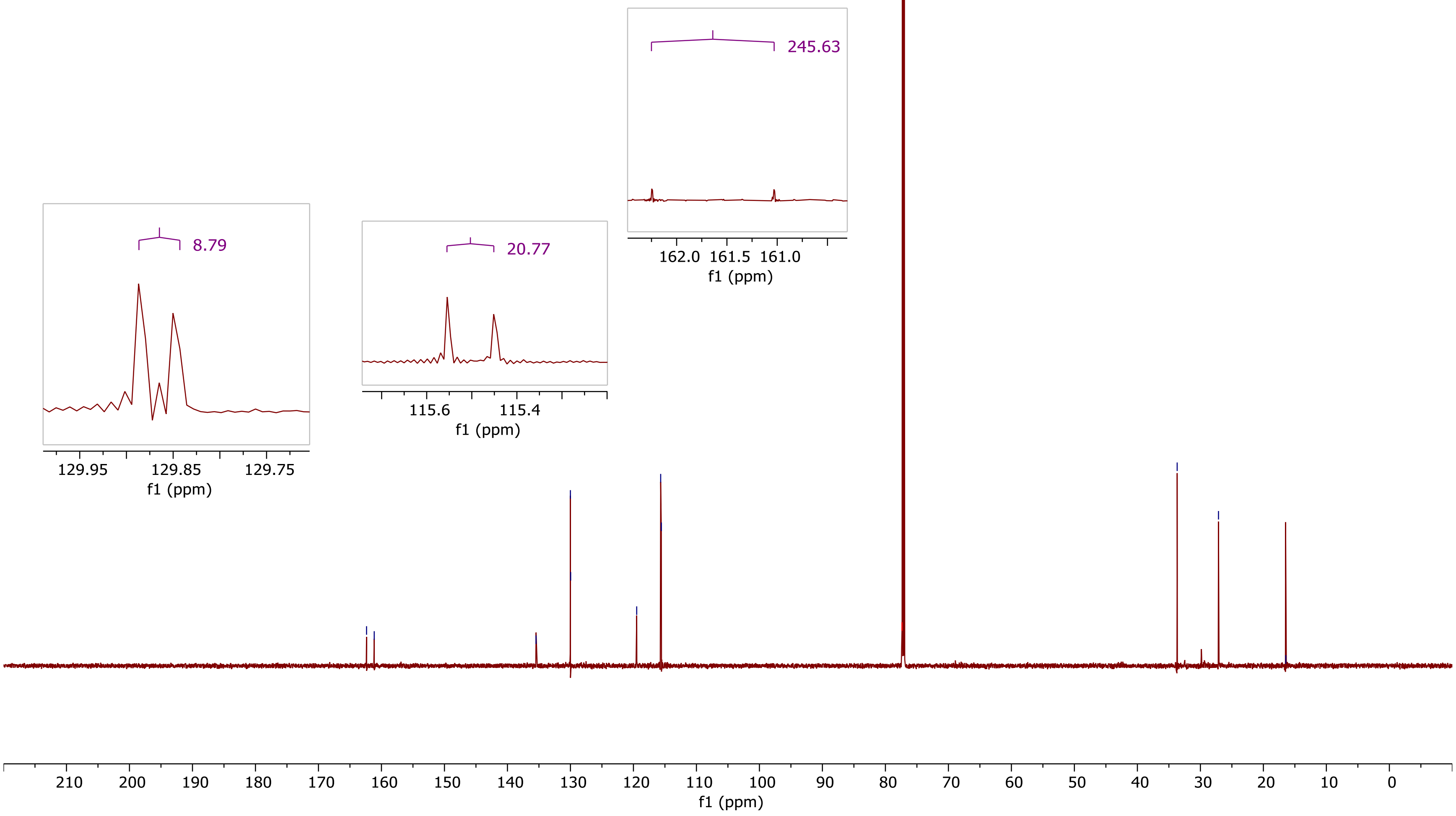

[3a]
19F NMR at 376.48 MHz in CDCl3

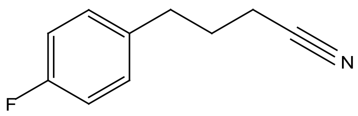

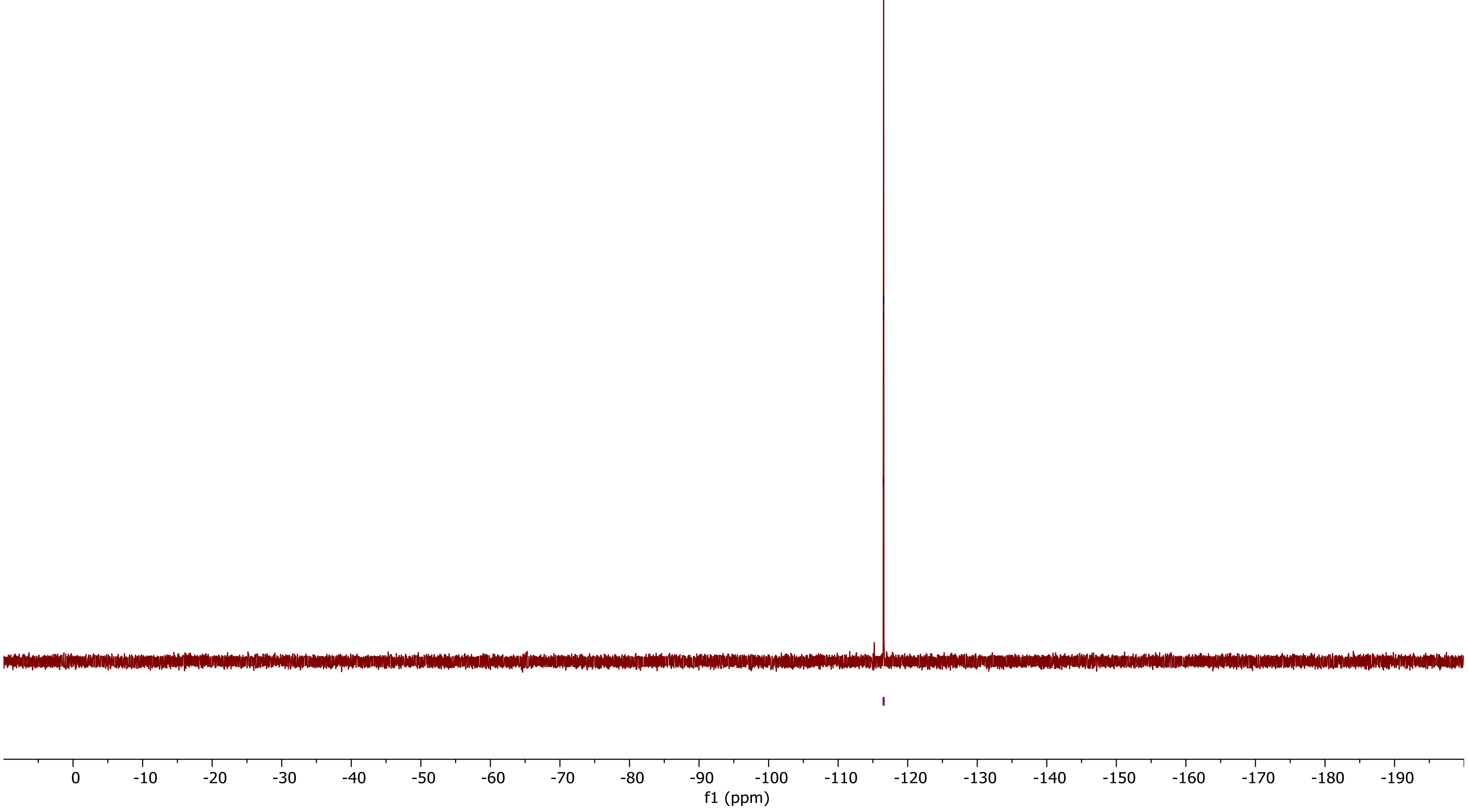

[3b]
1H NMR at 400.15 MHz in CDCl3

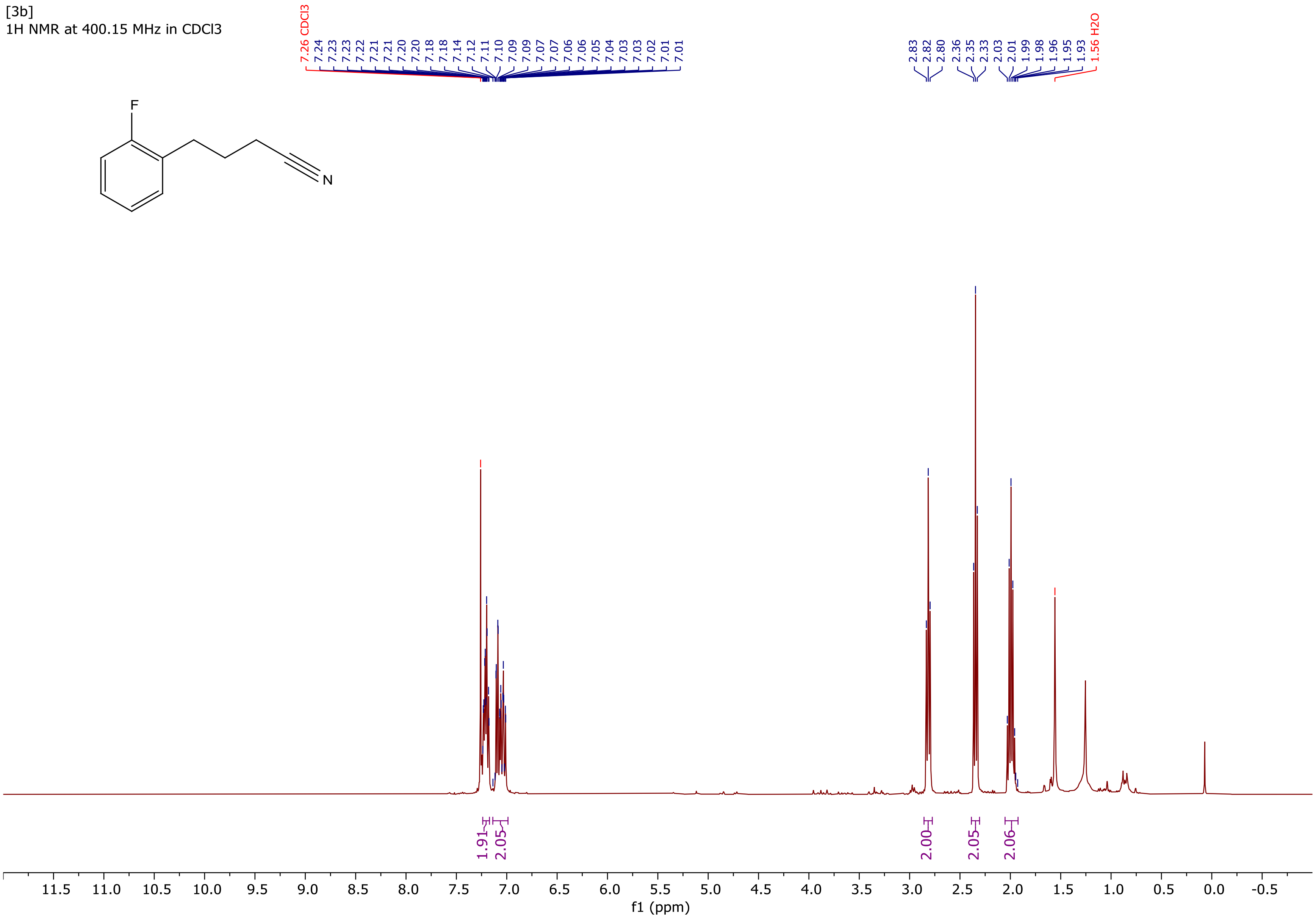

[3b]
13C NMR at 201.27 MHz in CDCl3

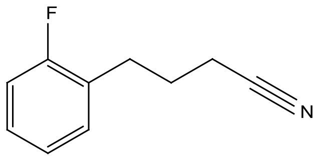

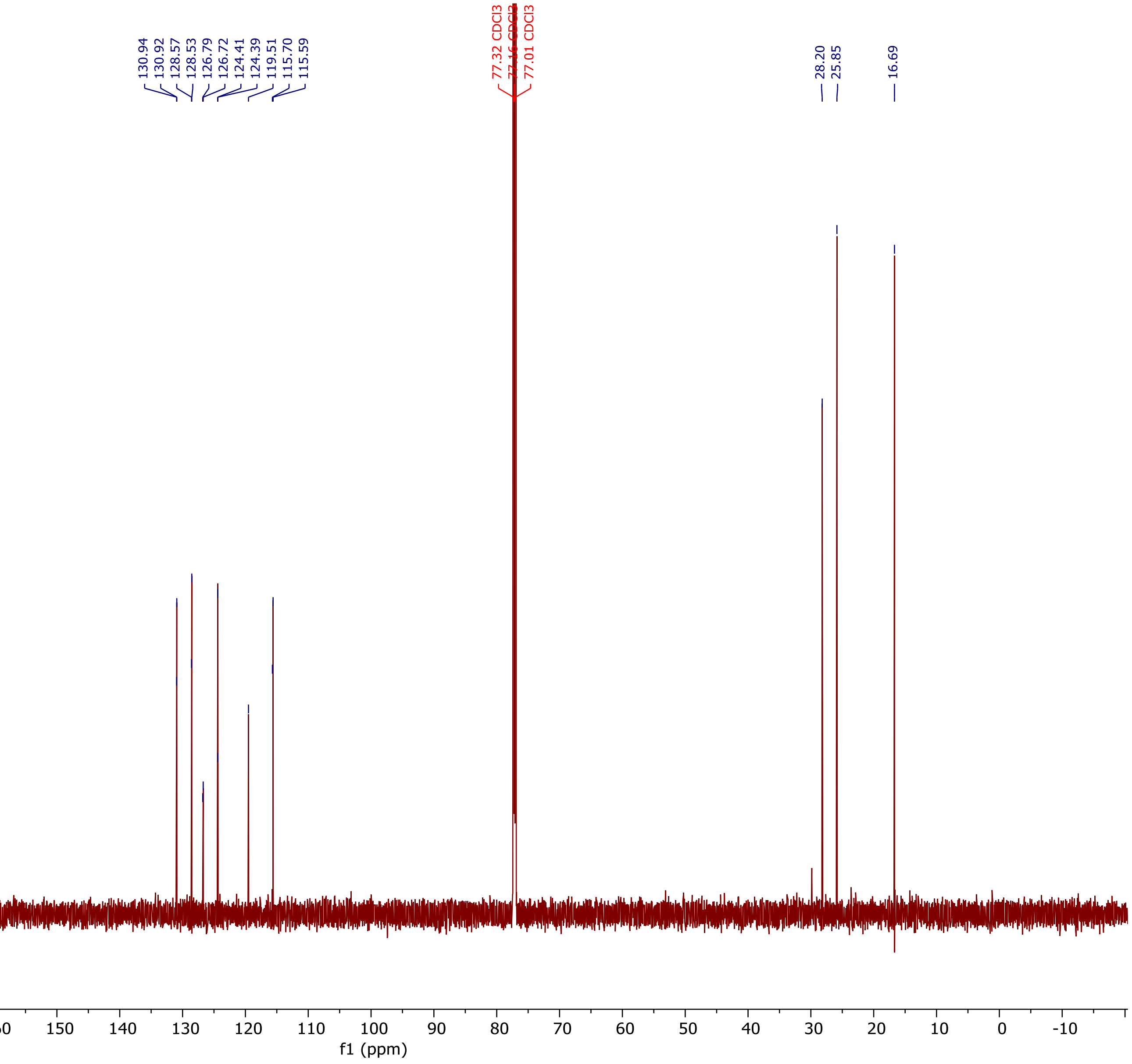

[3b]
19F NMR at 376.48 MHz in CDCl3

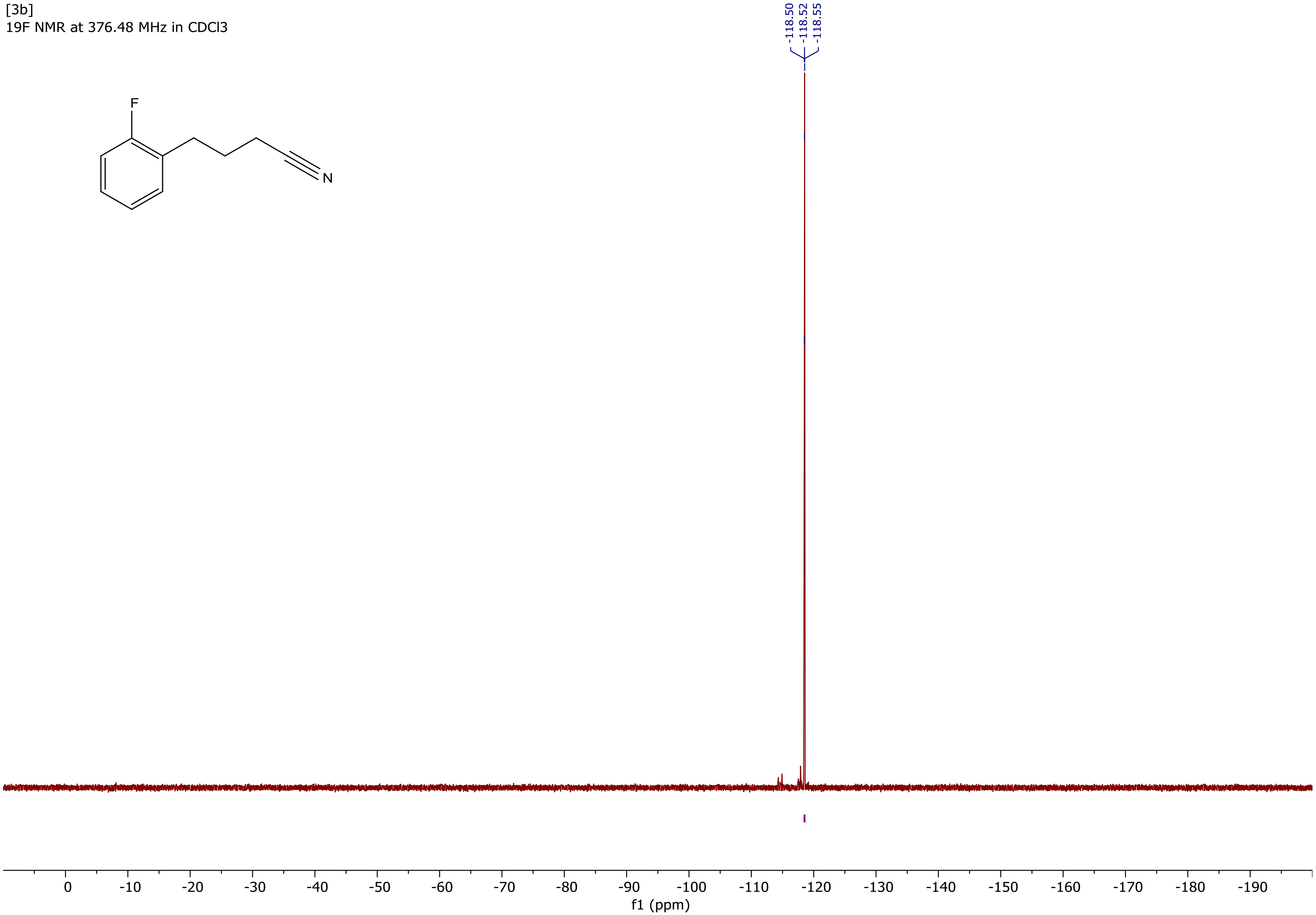

[3c]
1H NMR at 400.15 MHz in CDCl3

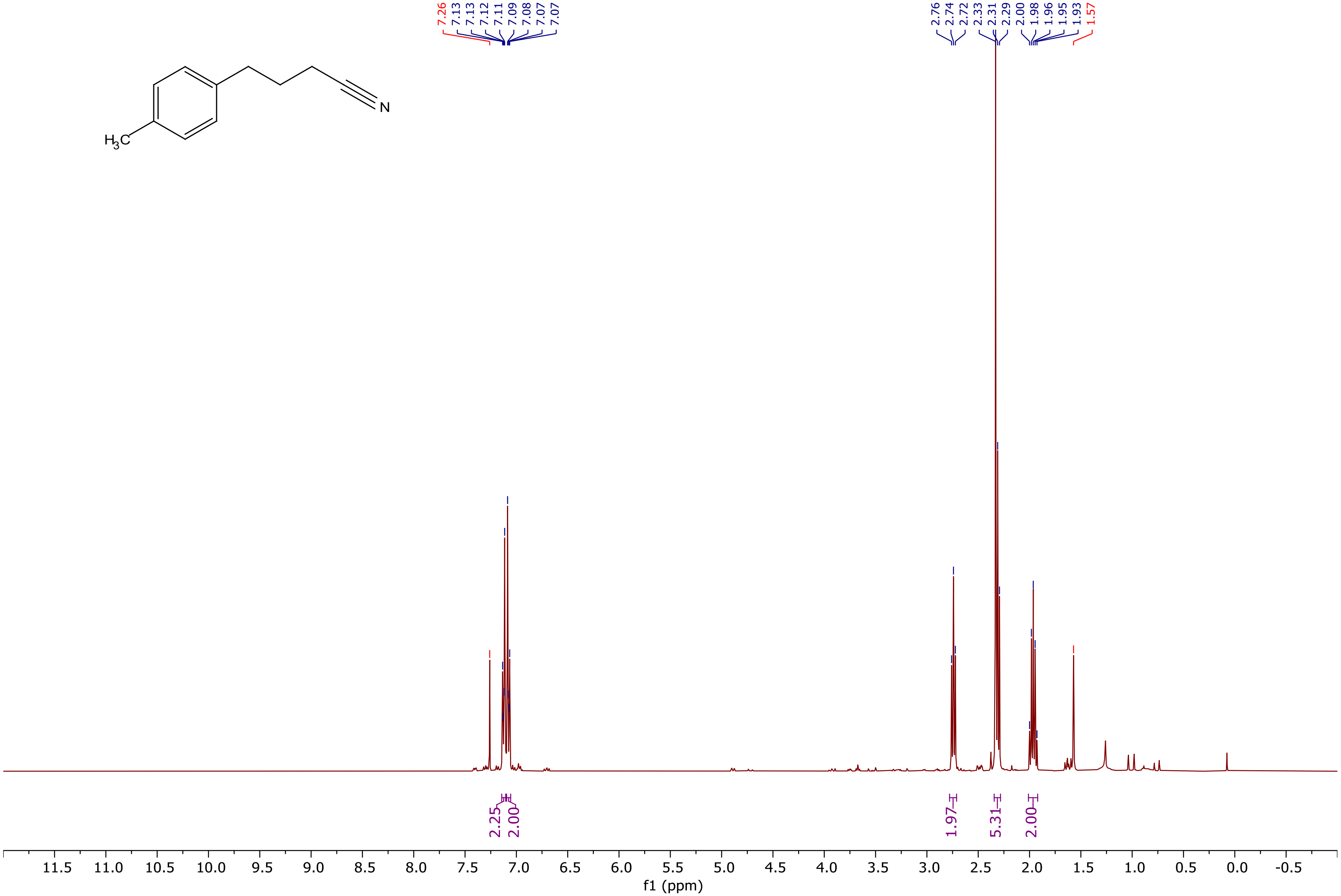

[3c]
13C NMR at 201.27 MHz in CDCl3

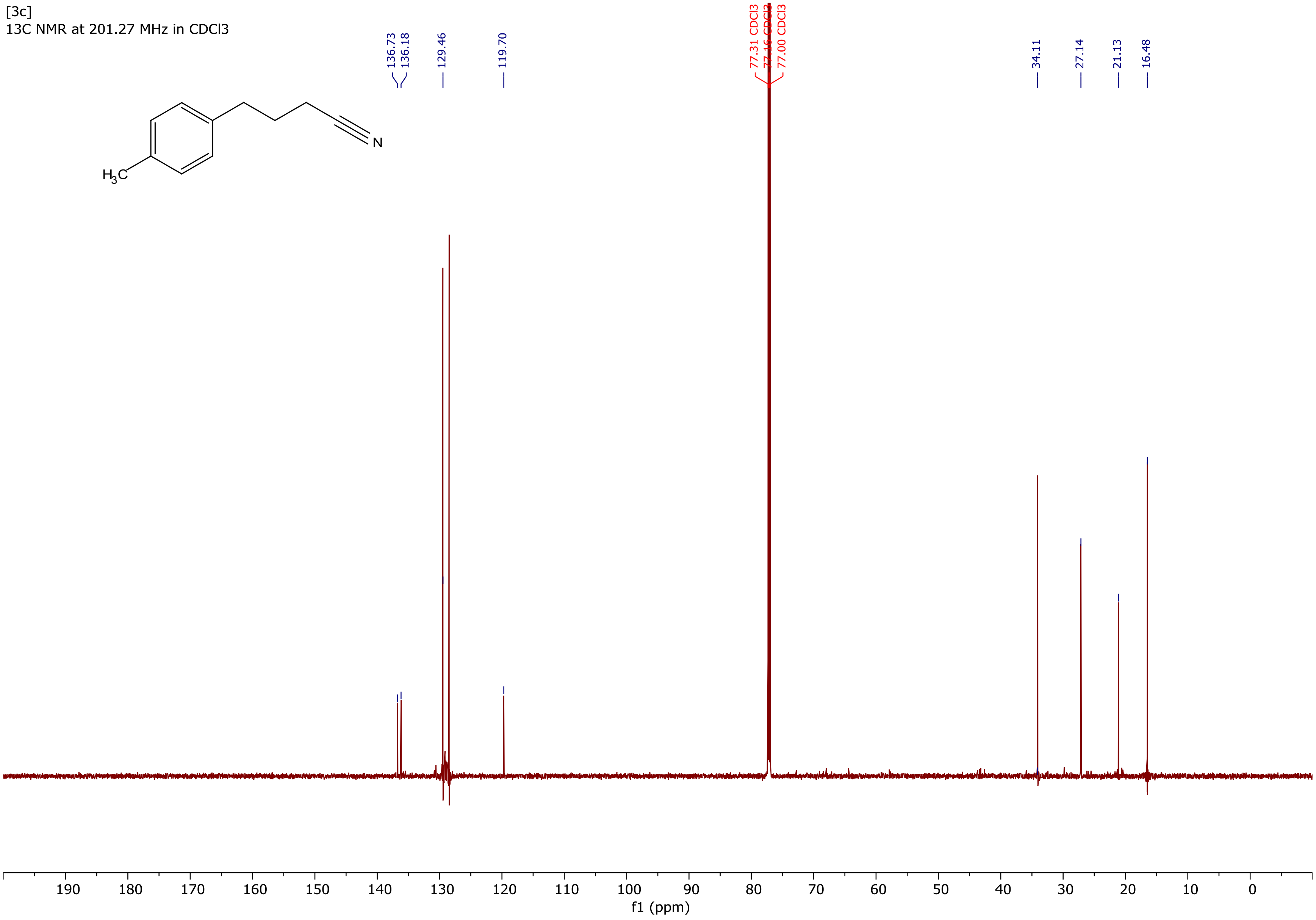

[3d]
1H NMR at 400.15 MHz in CDCl3

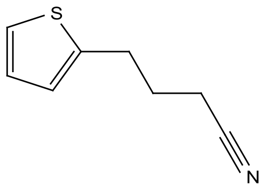

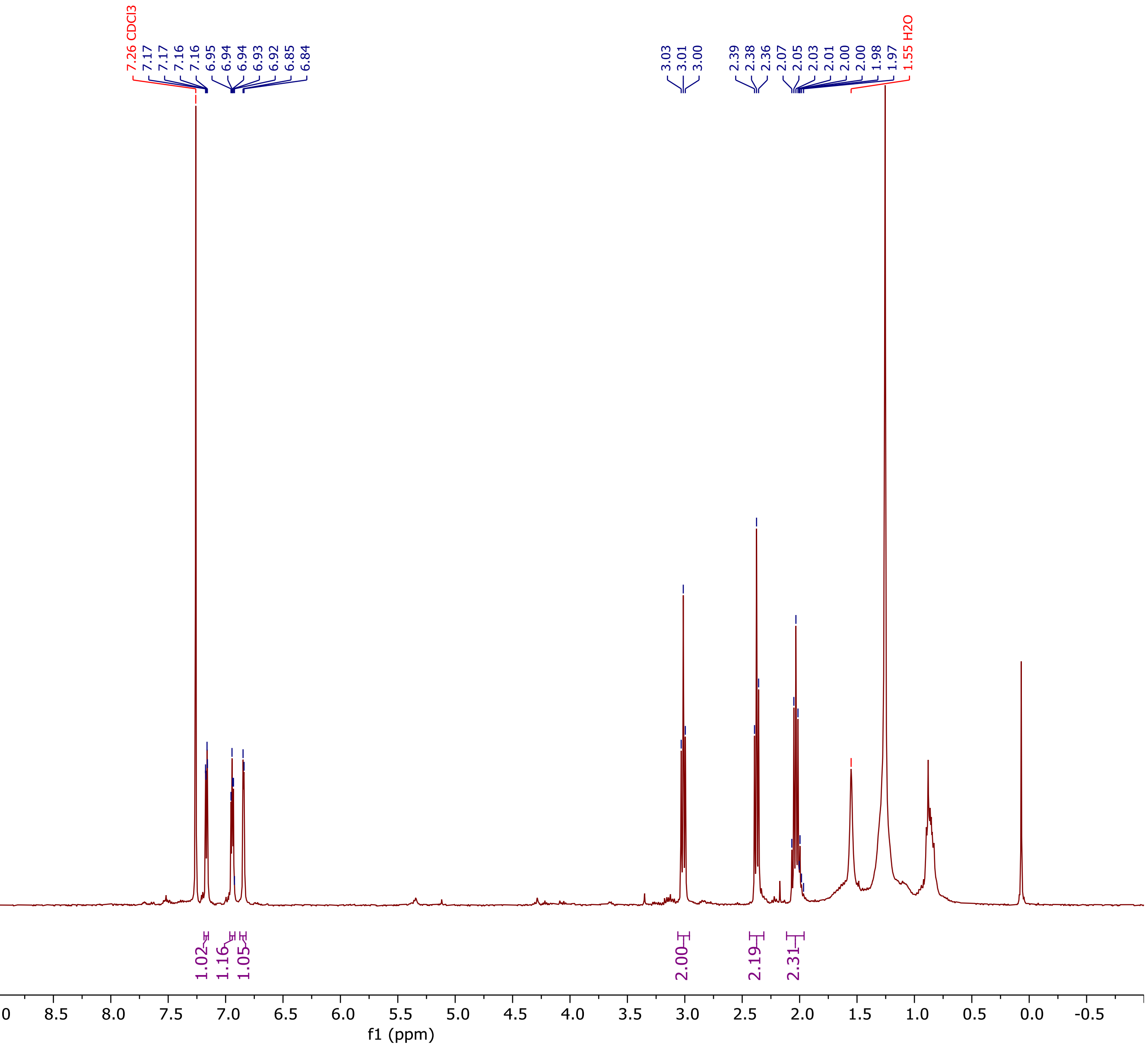

[3d]
1H NMR at 400.15 MHz in CDCl3

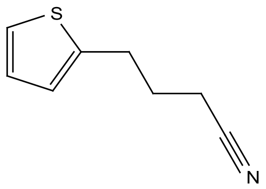


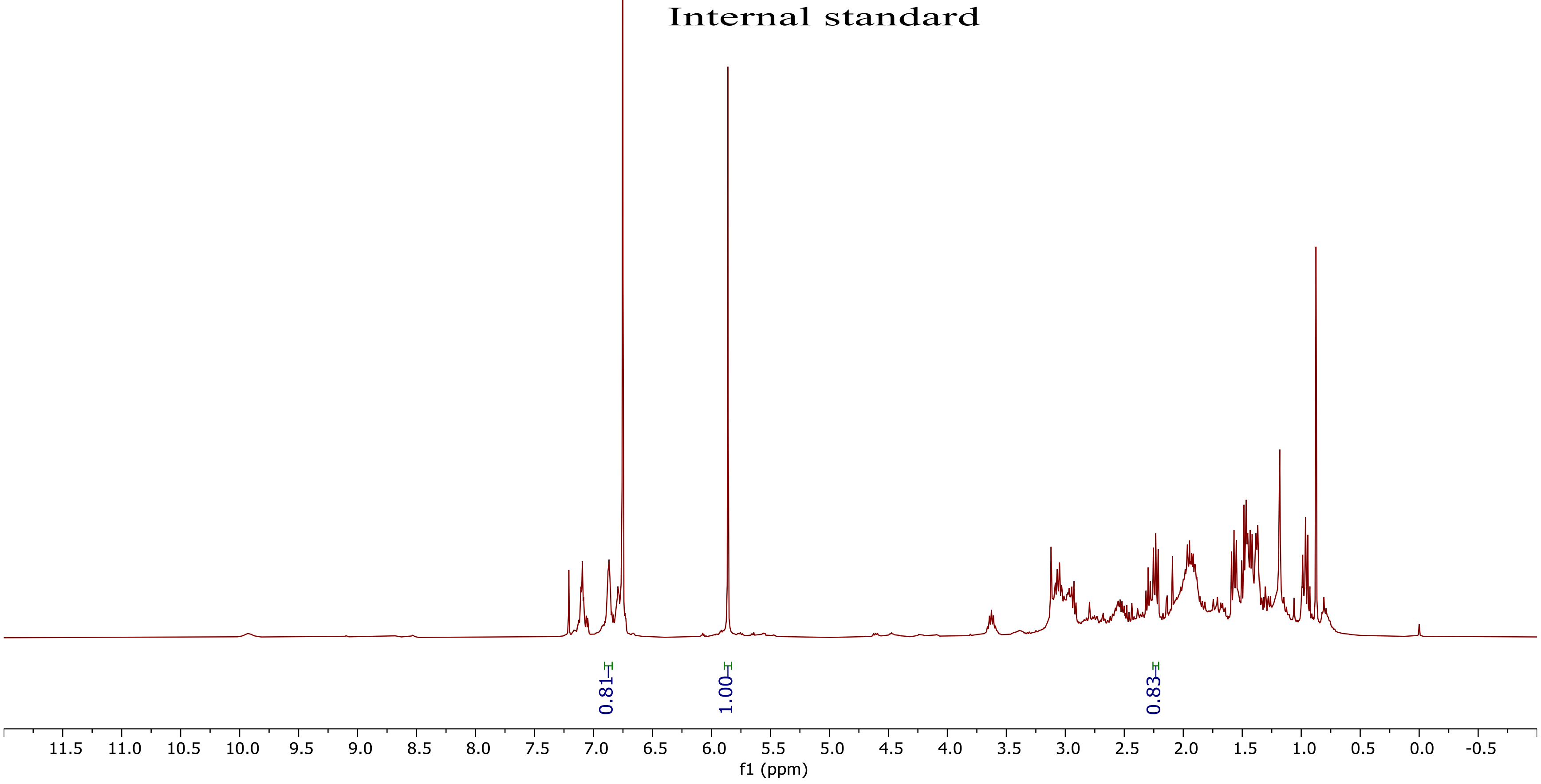

[3d]
13C NMR at 201.27 MHz in CDCl3

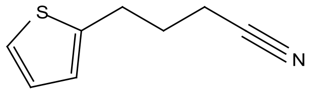

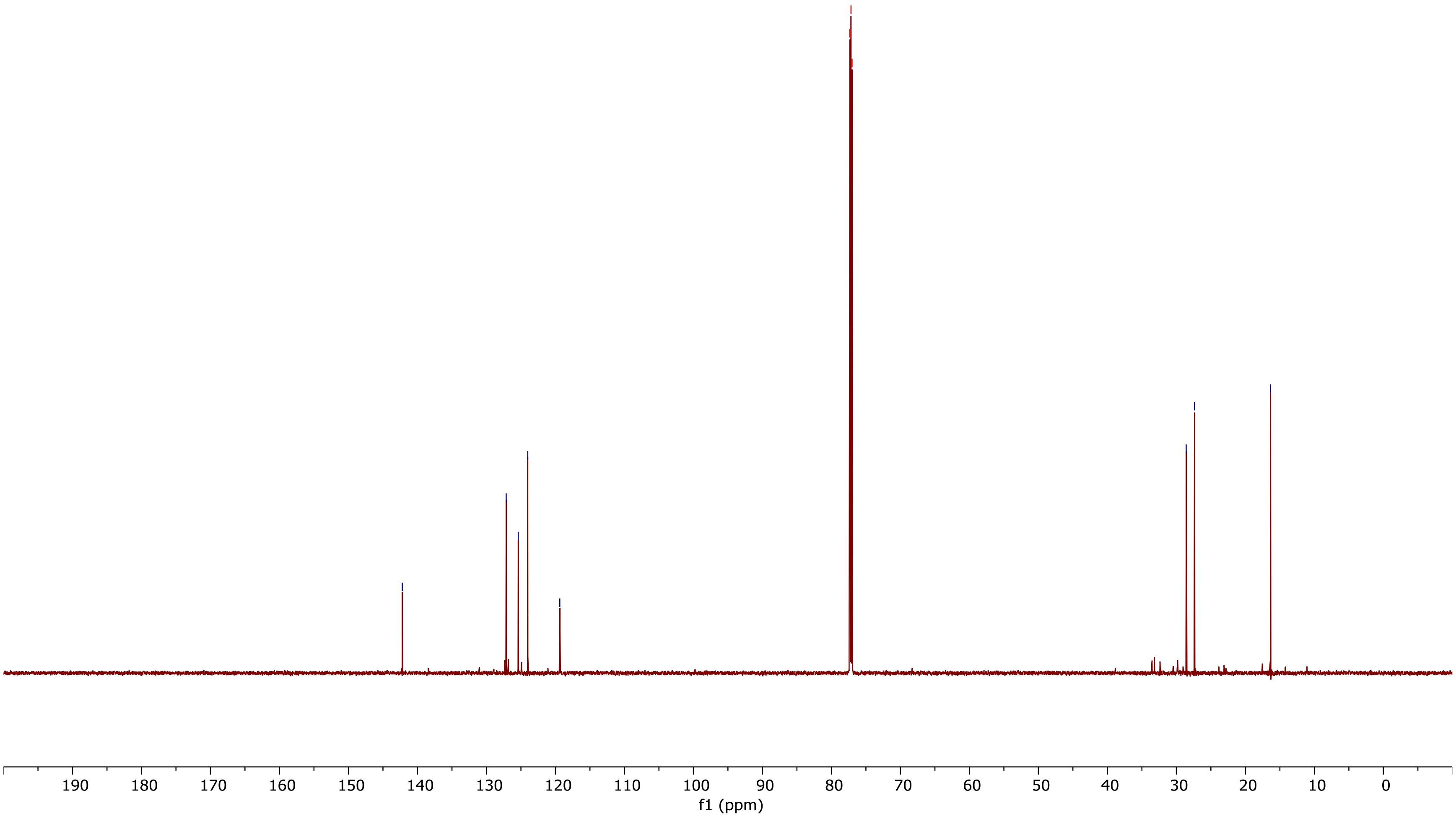

[3e]
1H NMR at 400.15 MHz in CDCl3

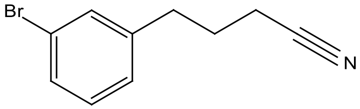


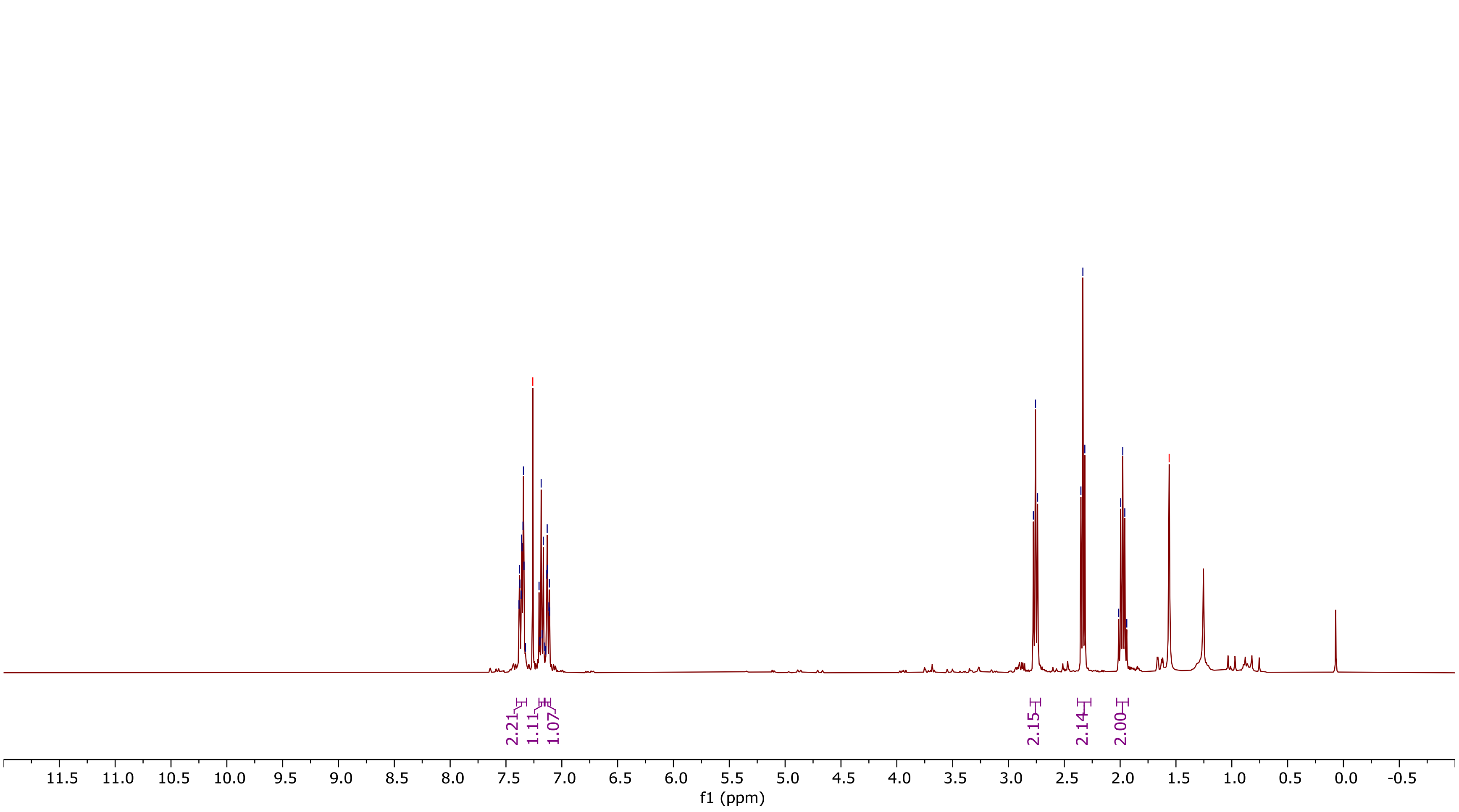

[3e]
13C NMR at 201.27 MHz in CDCl3

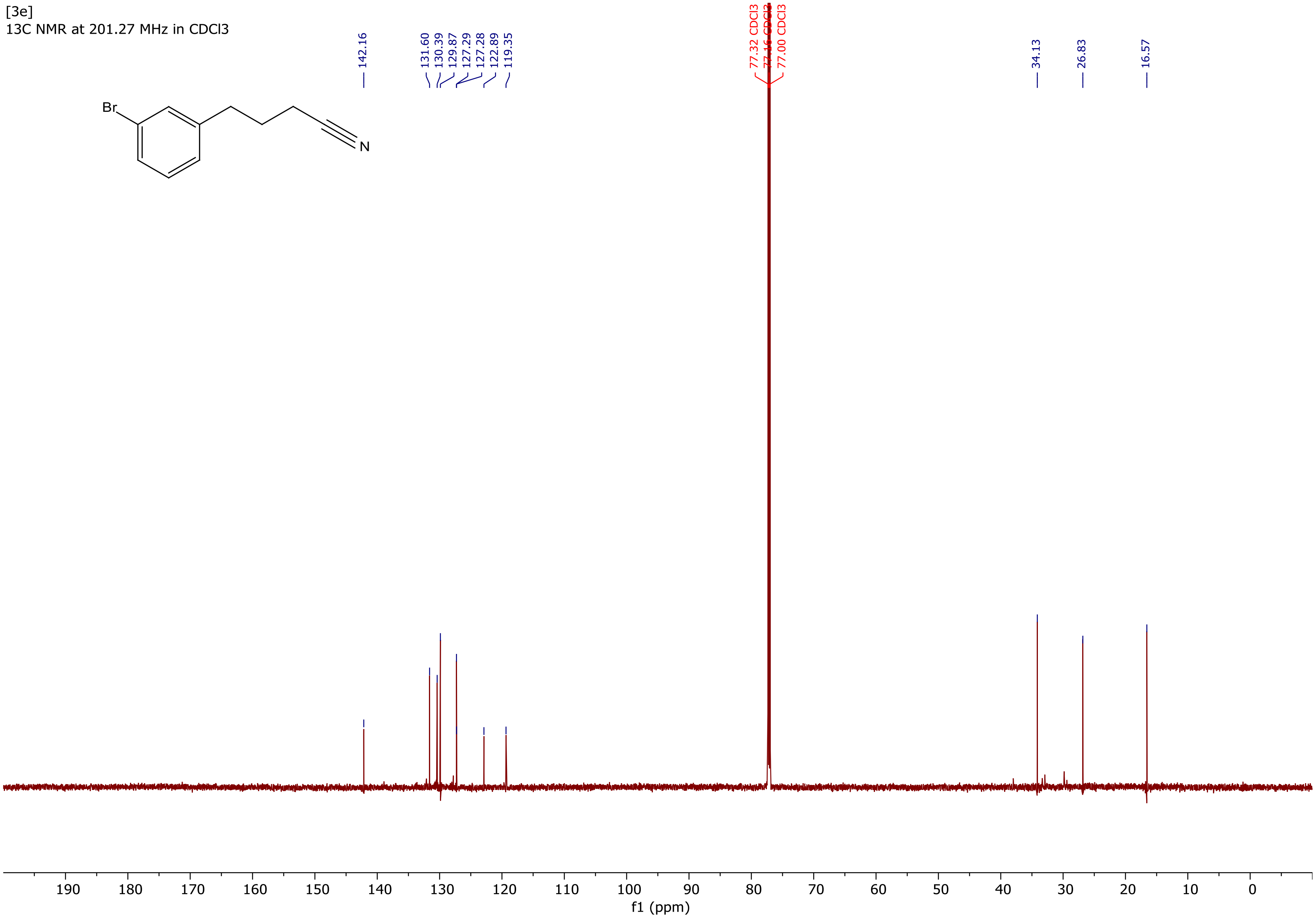

[3f]
1H NMR at 400.15 MHz in CDCl3

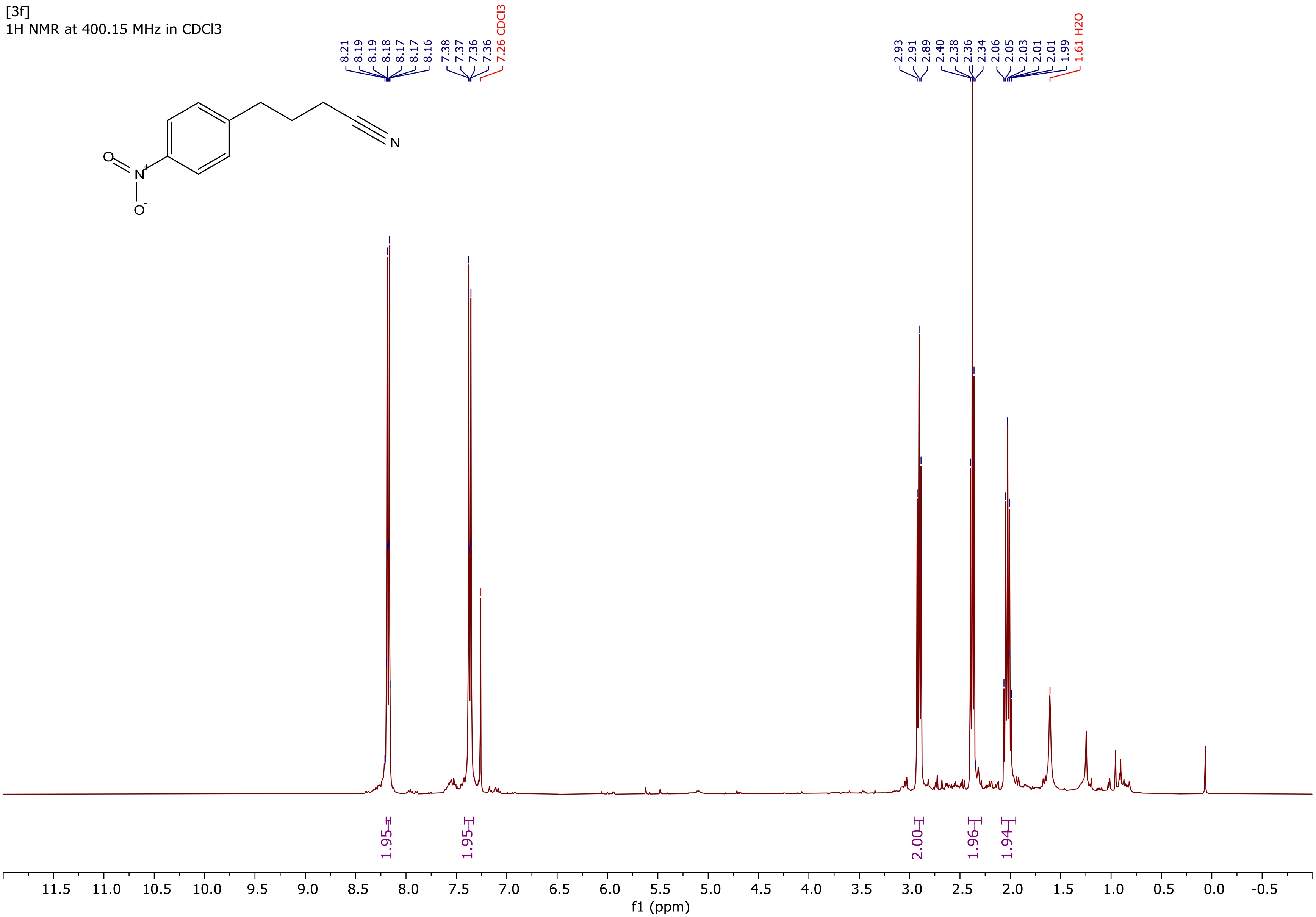

[3f]
13C NMR at 201.27 MHz in CDCl3

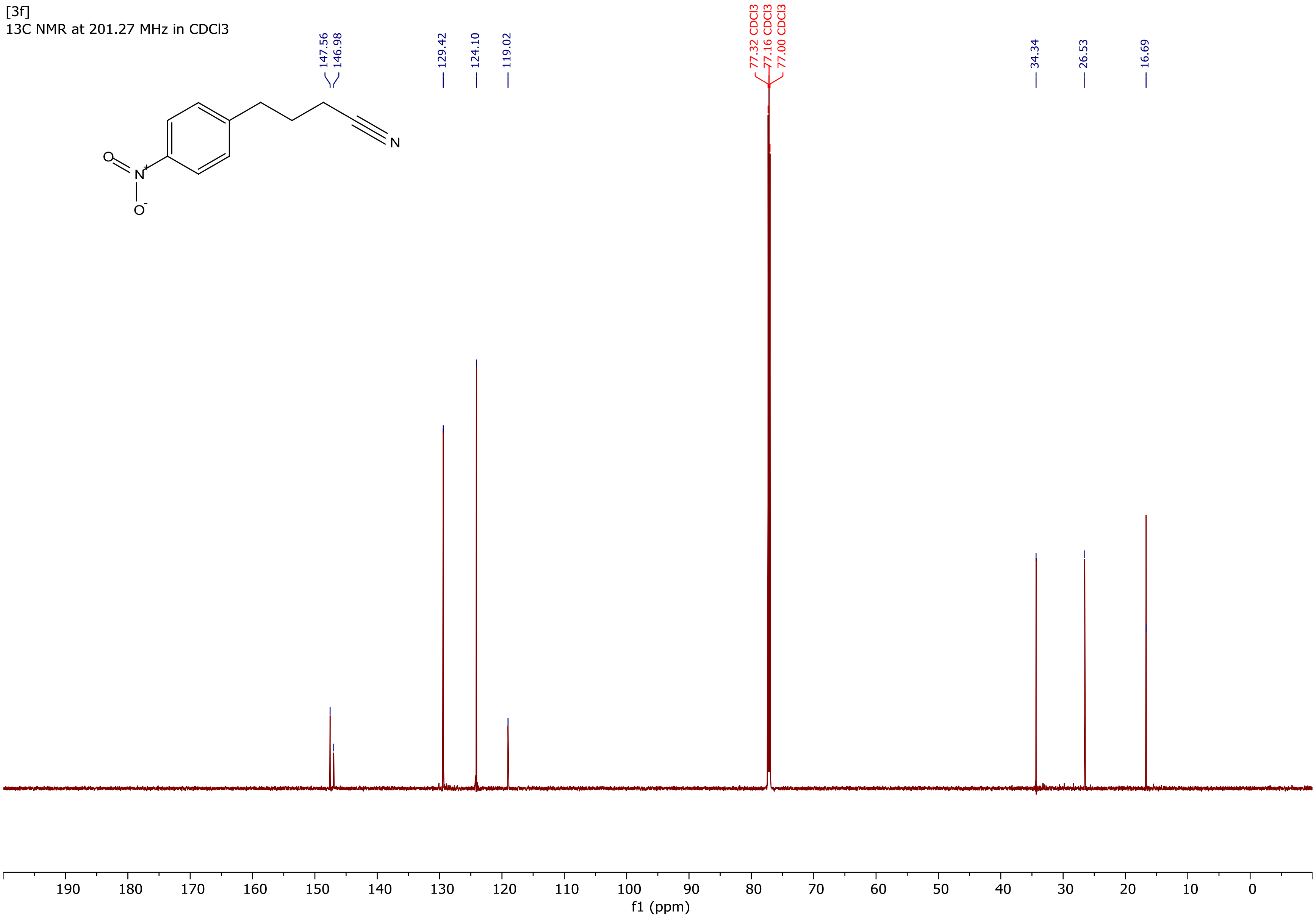

[3g]
1H NMR at 400.15 MHz in CDCl3

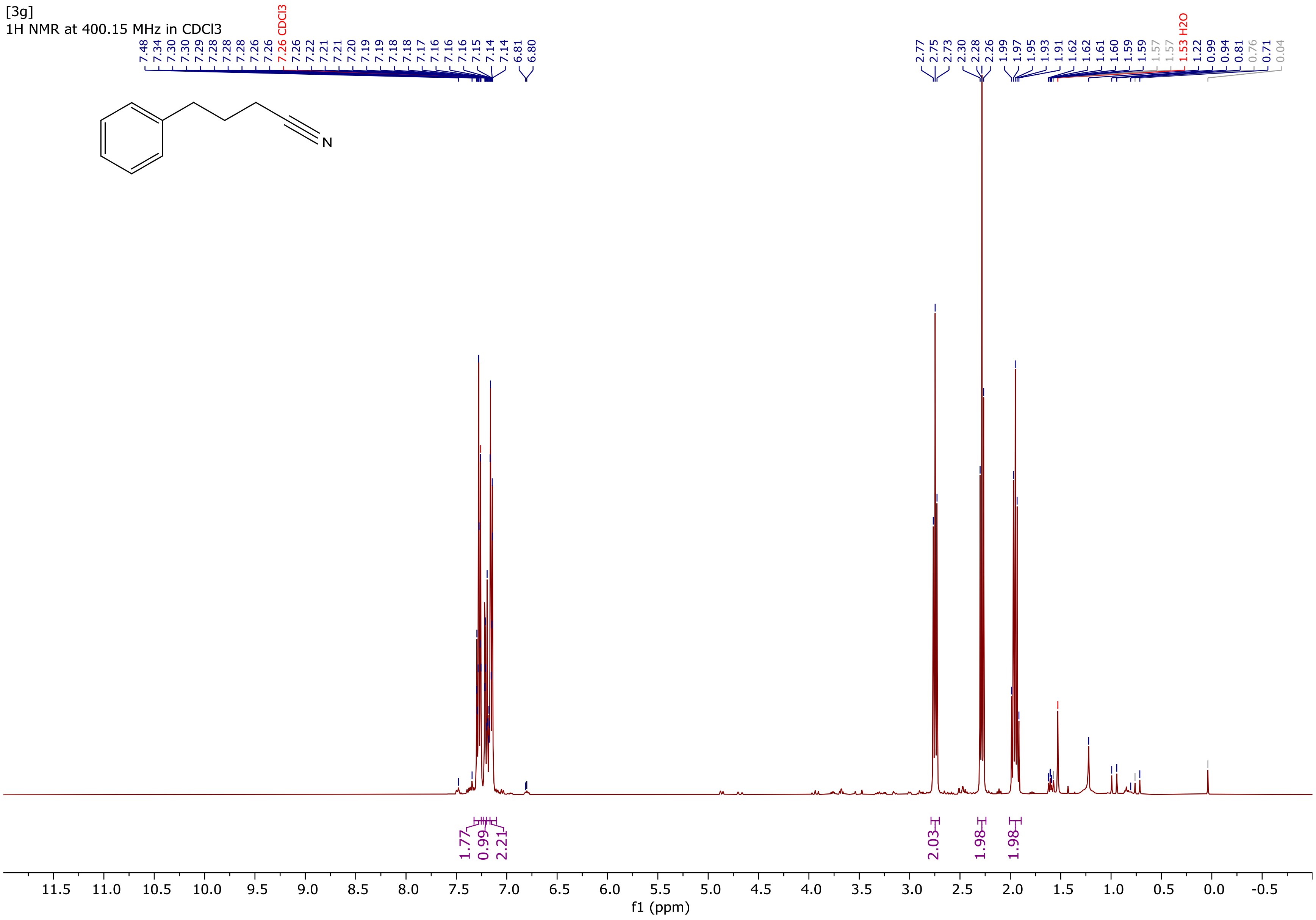

[3g]
13C NMR at 201.27 MHz in CDCl3

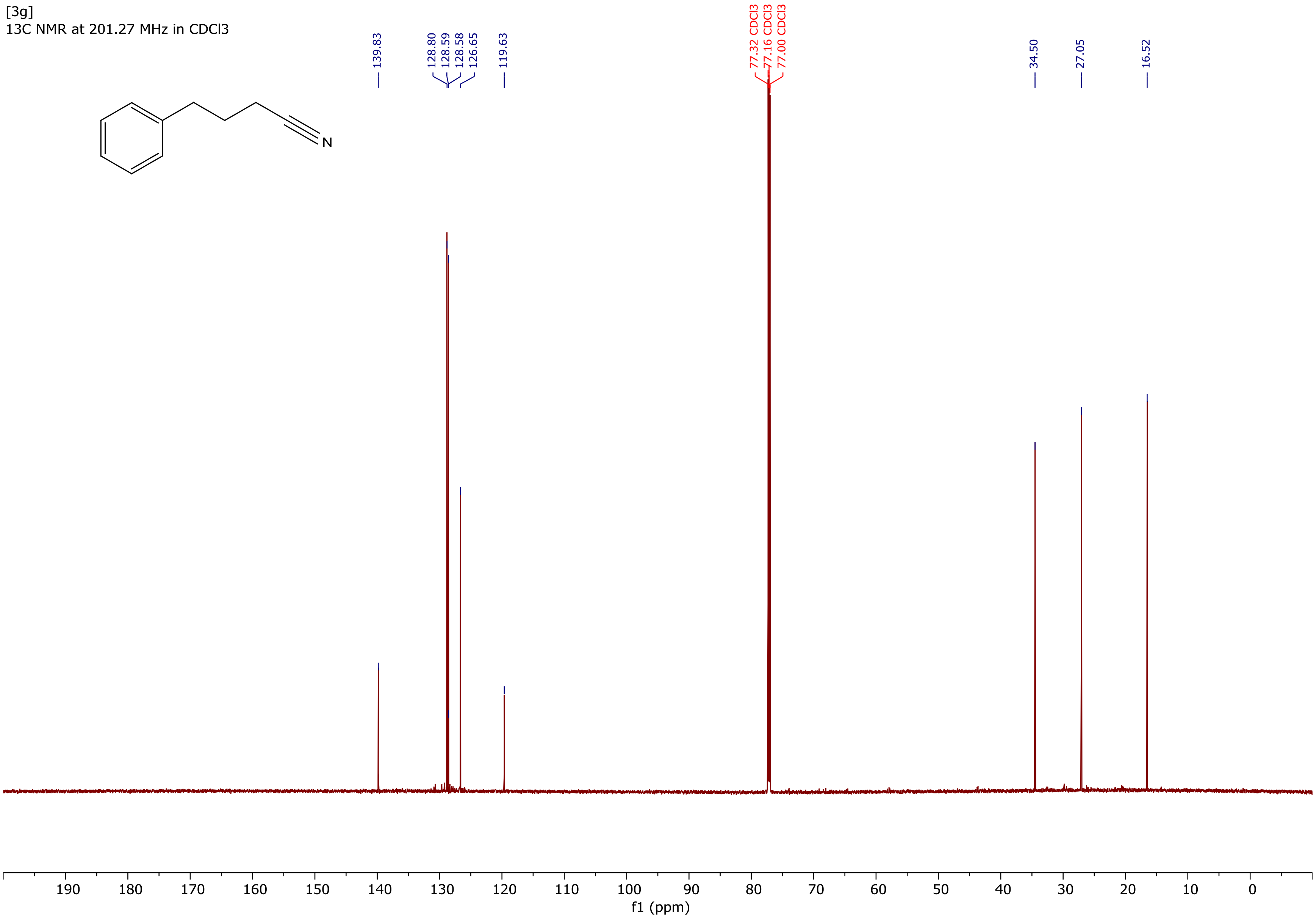

[3h]
1H NMR at 400.15 MHz in CDCl3

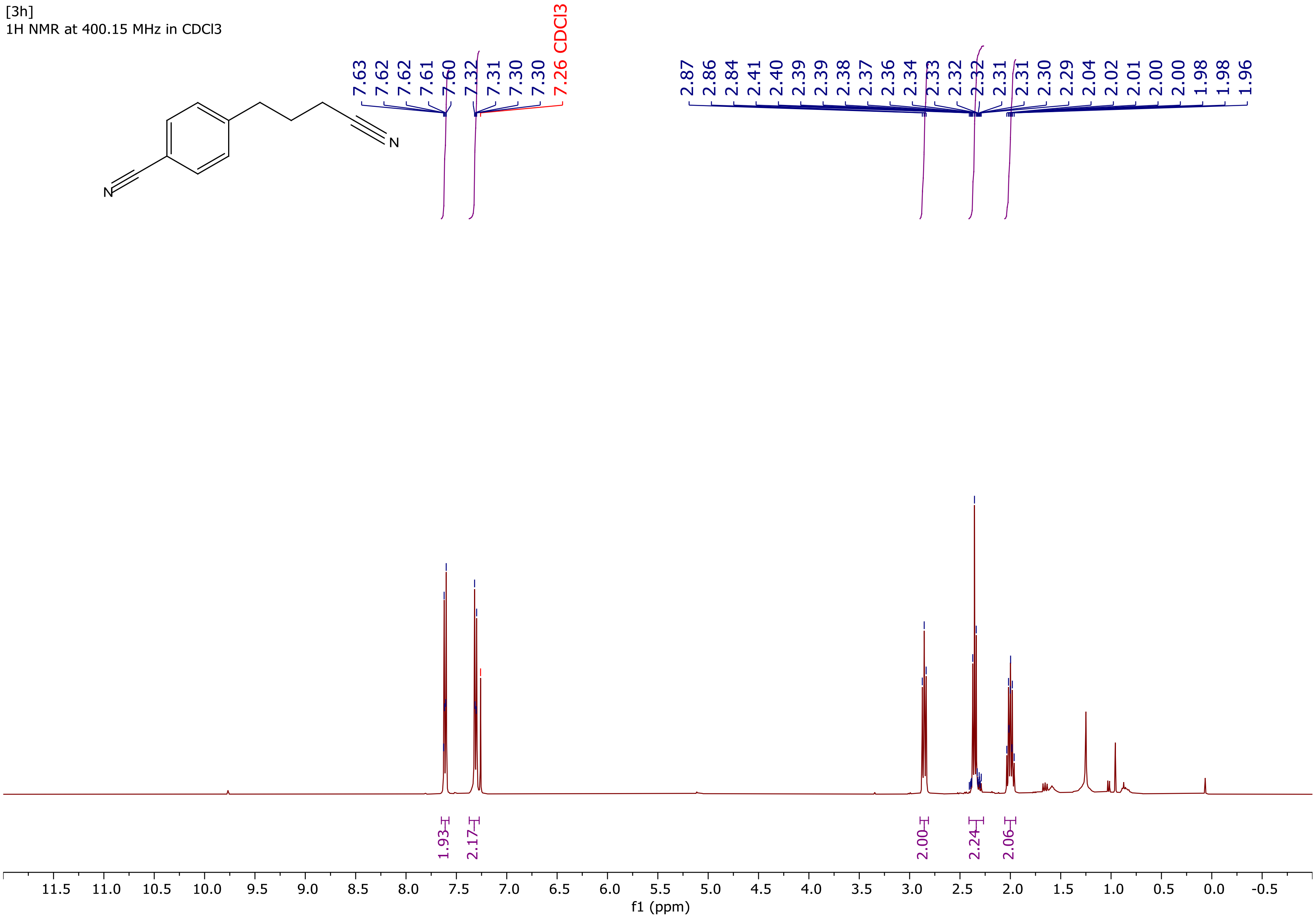

[3h]
13C NMR at 201.27 MHz in CDCl3

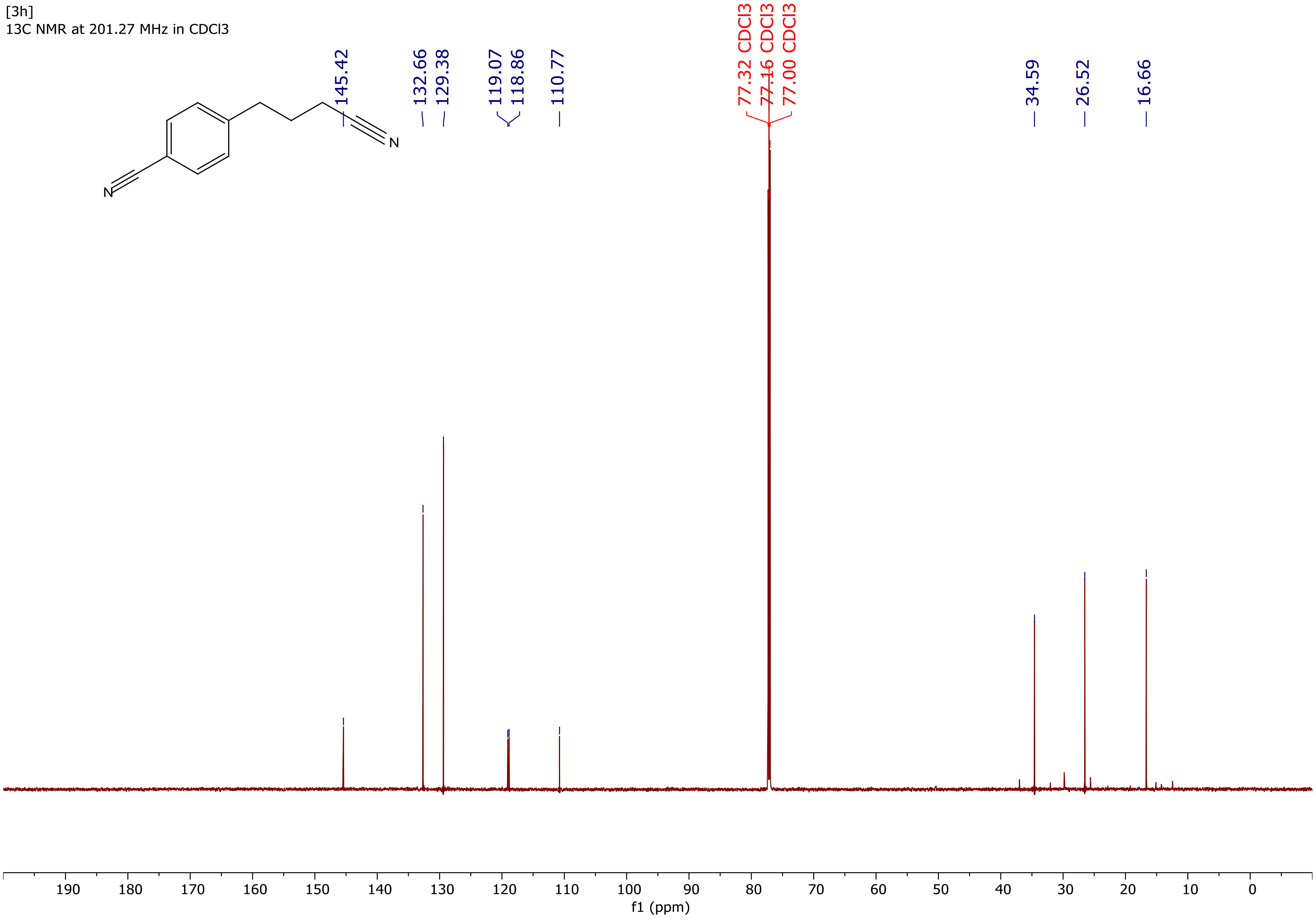

[3i]
1H NMR at 400.15 MHz in CDCl3

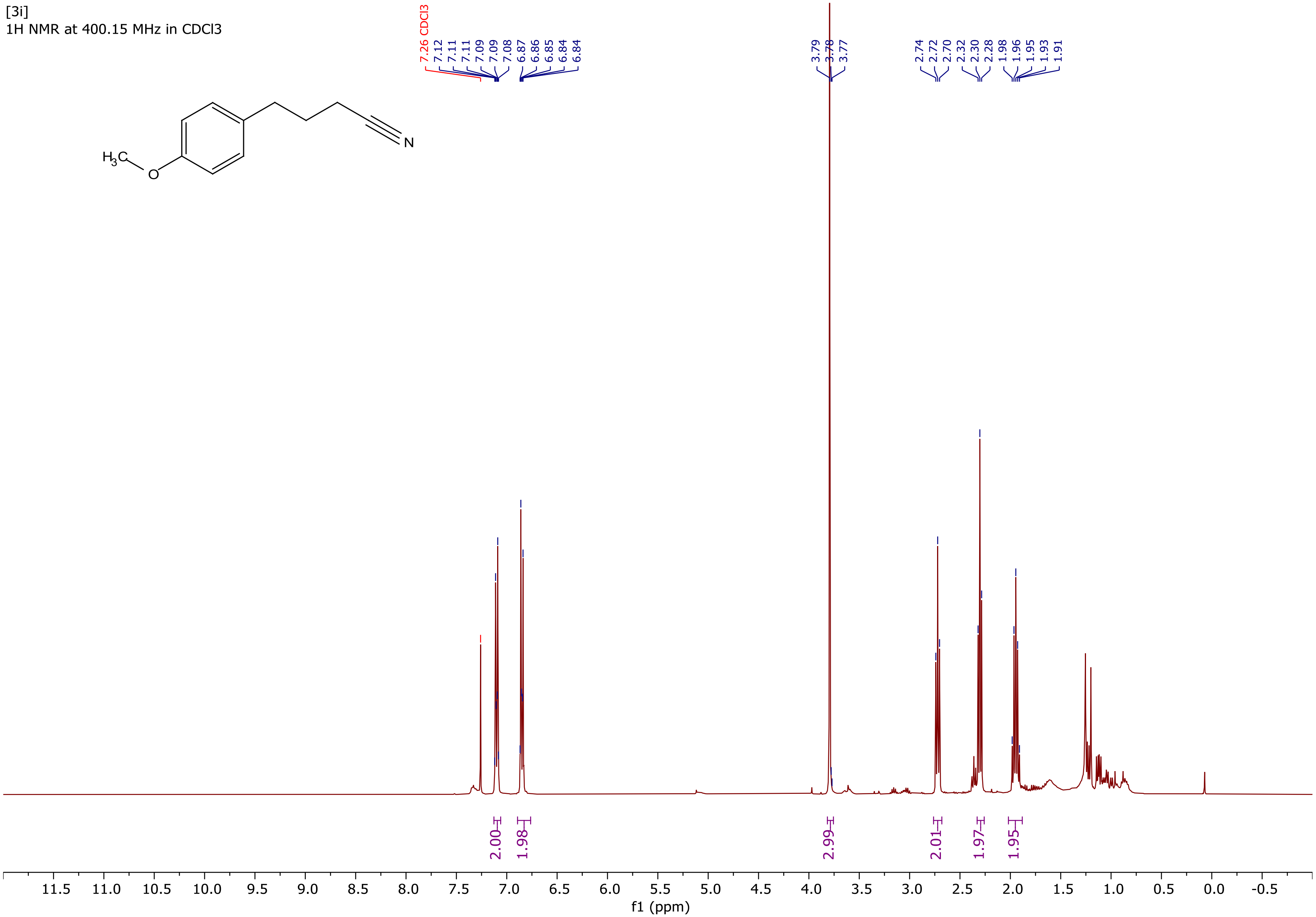

[3i]
13C NMR at 201.27 MHz in CDCl3

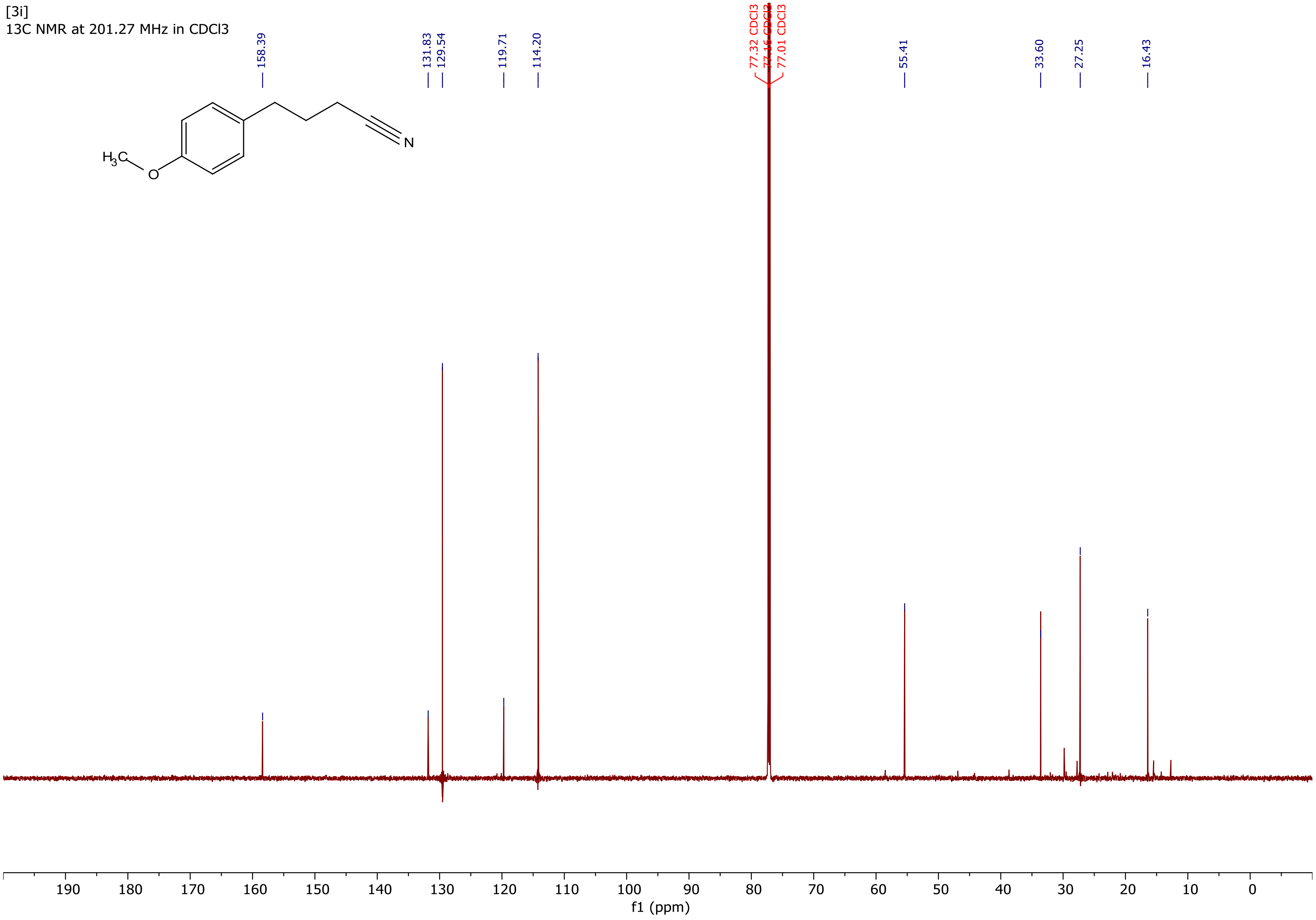

[3j]
1H NMR at 400.15 MHz in CDCl3

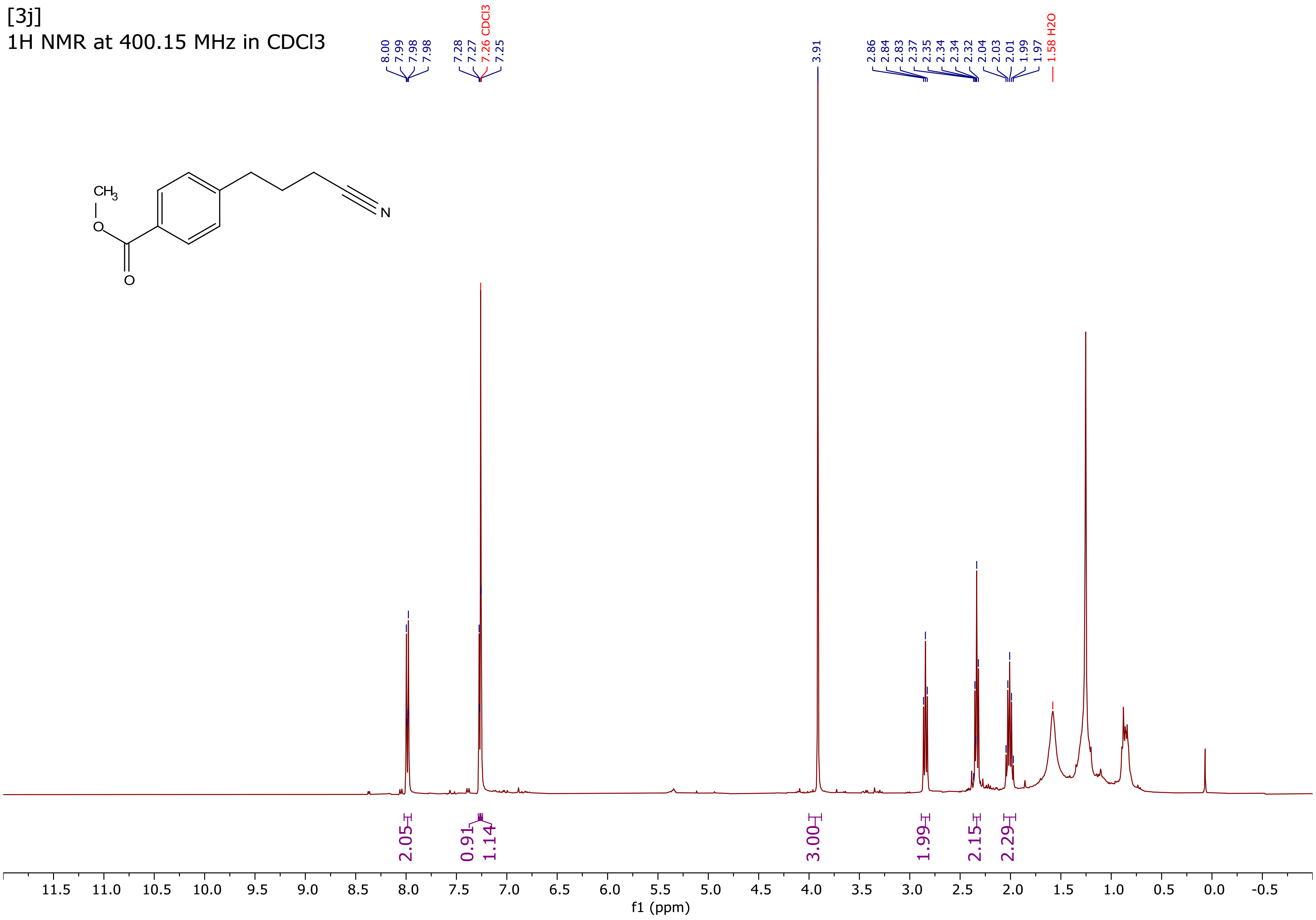

[3j]
13C NMR at 201.27 MHz in CDCl3

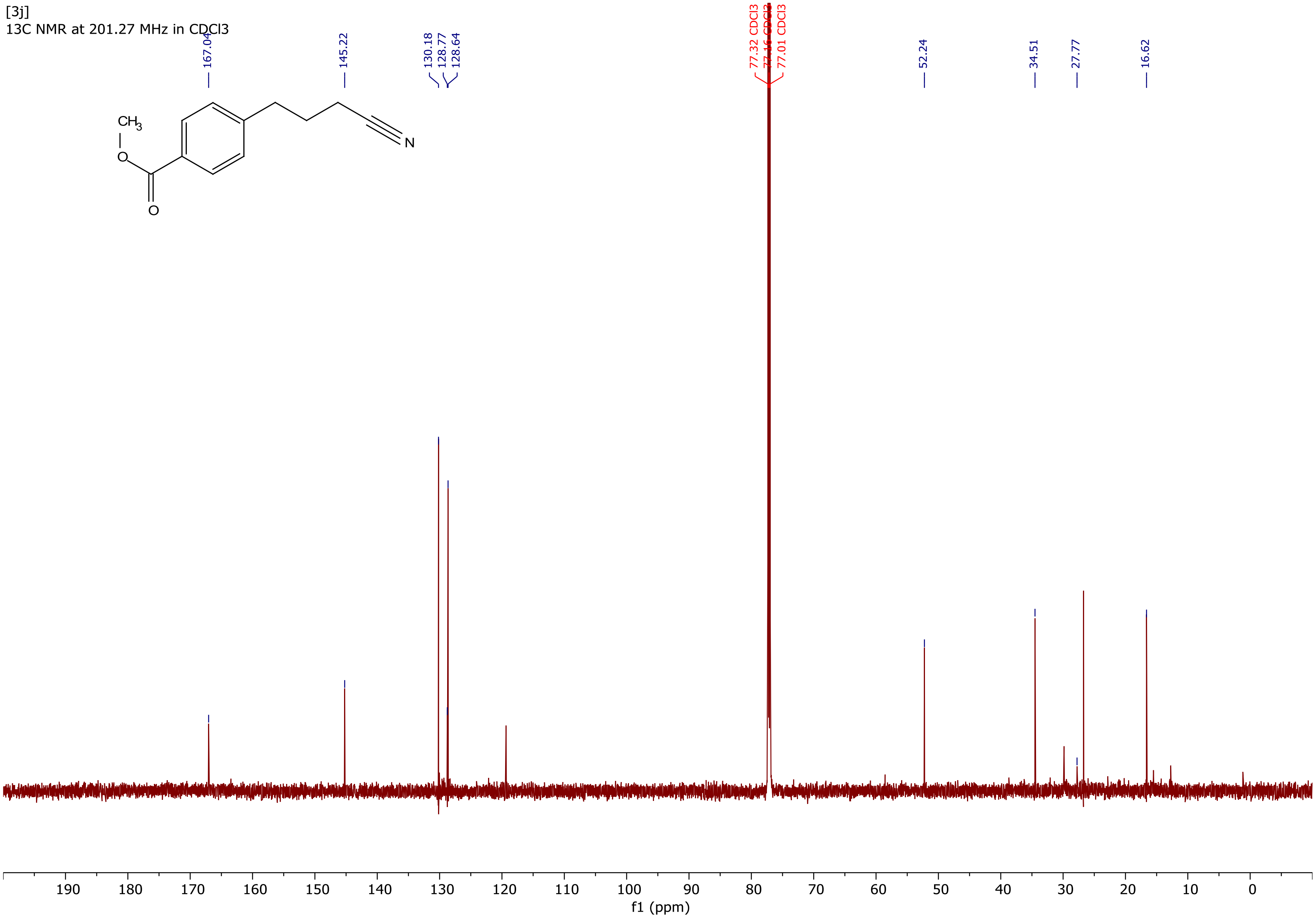

[3k]
1H NMR at 400.15 MHz in CDCl3

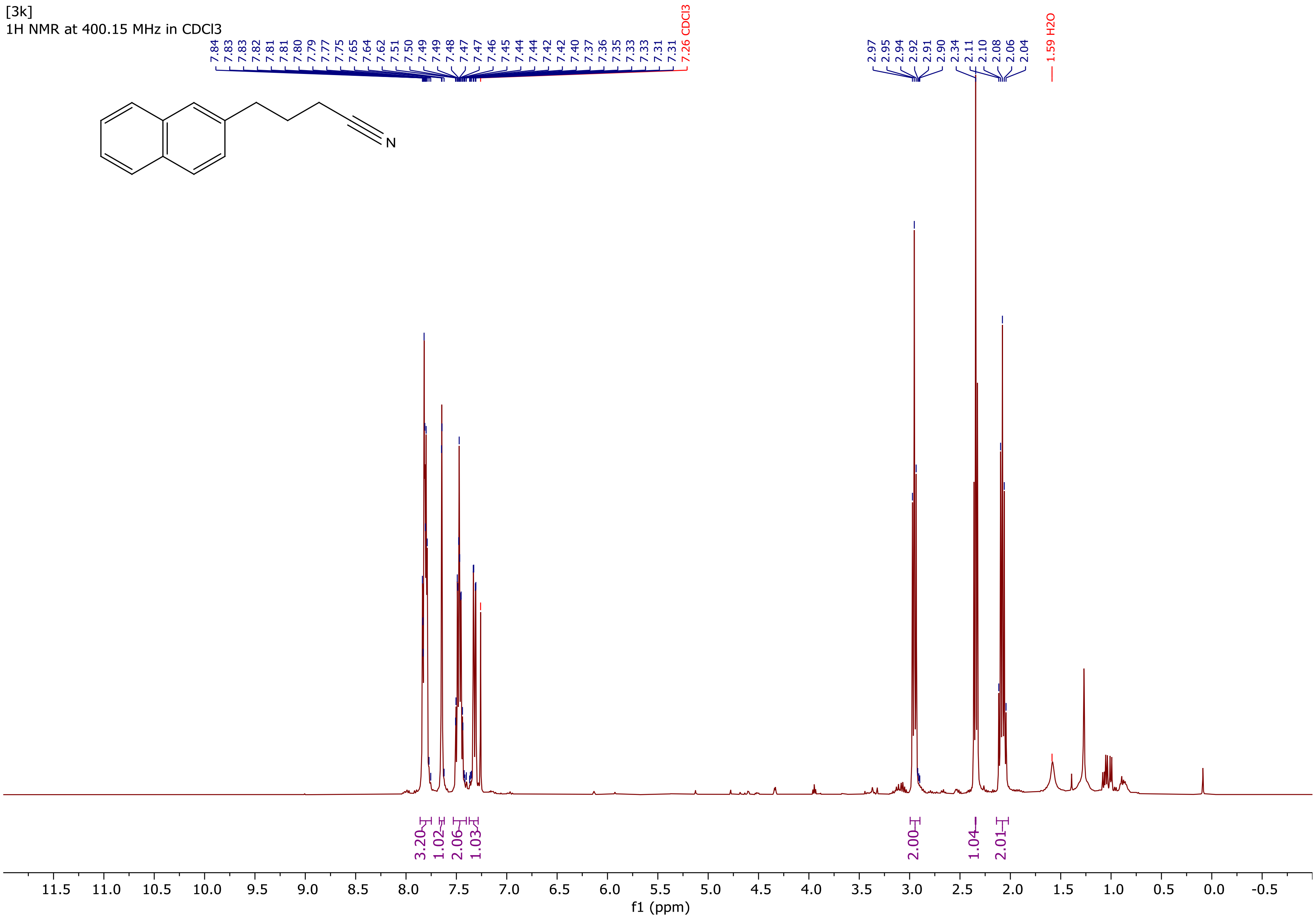

[3k]
13C NMR at 201.27 MHz in CDCl3

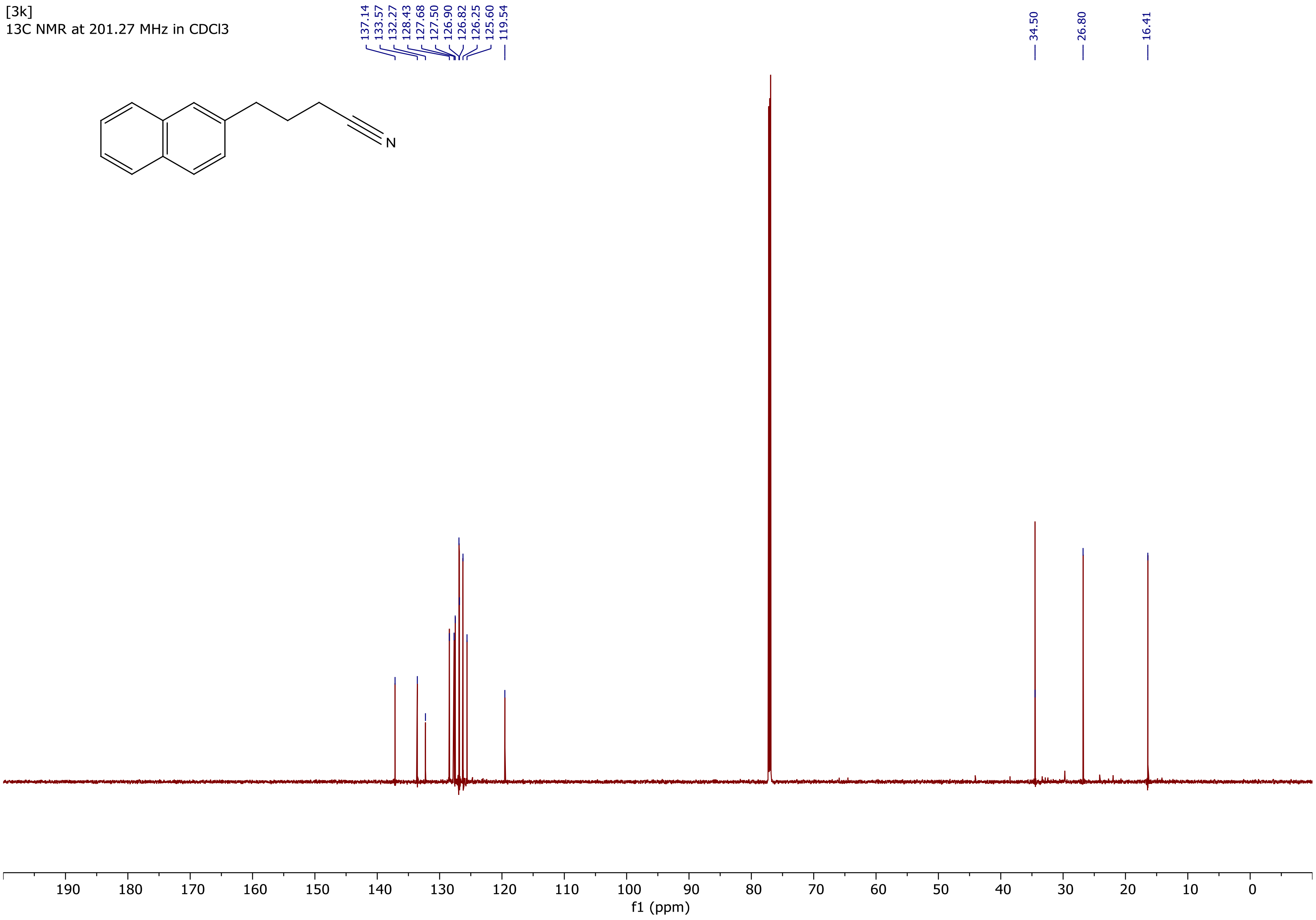

[3l]
1H NMR at 400.15 MHz in CDCl3

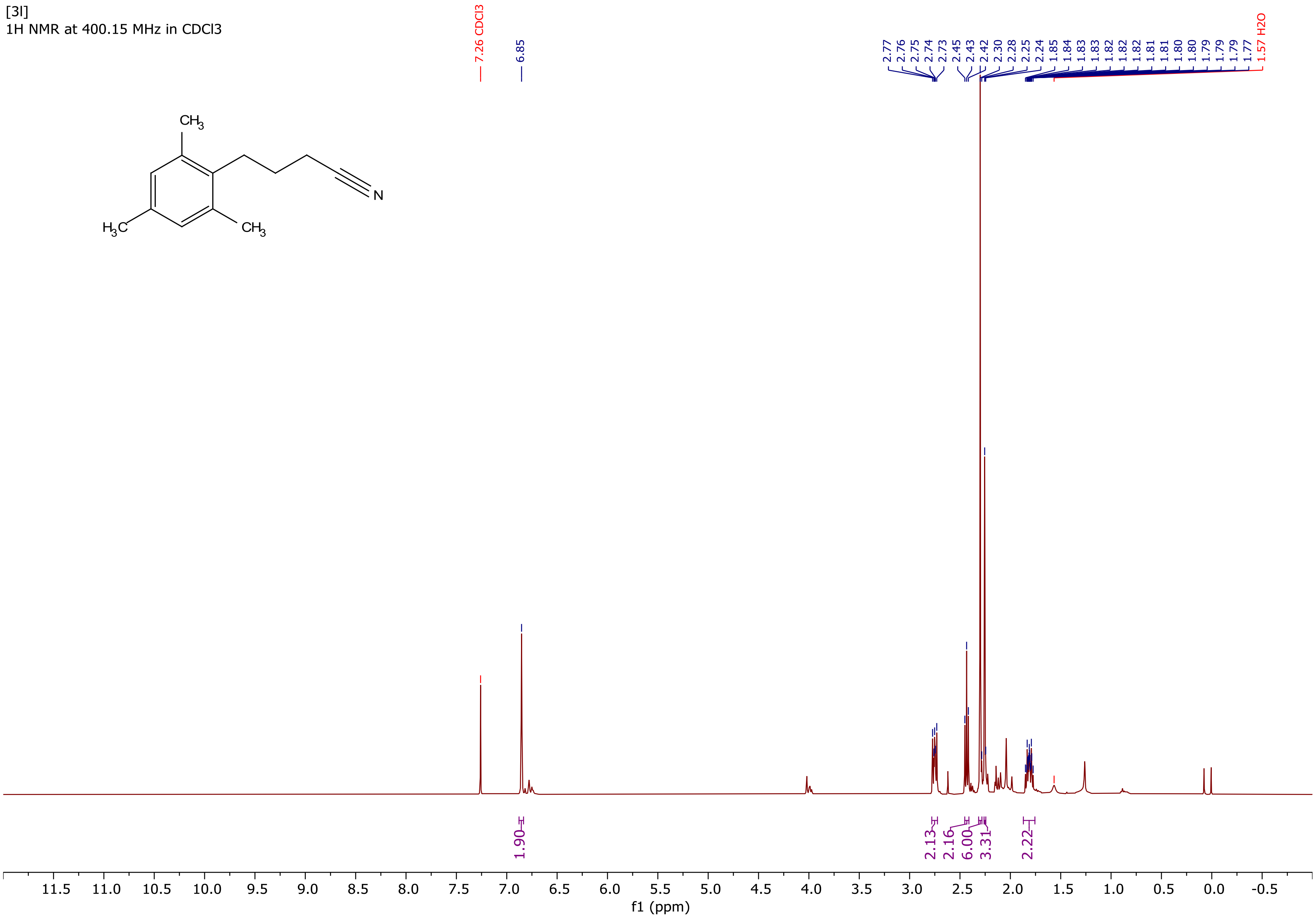

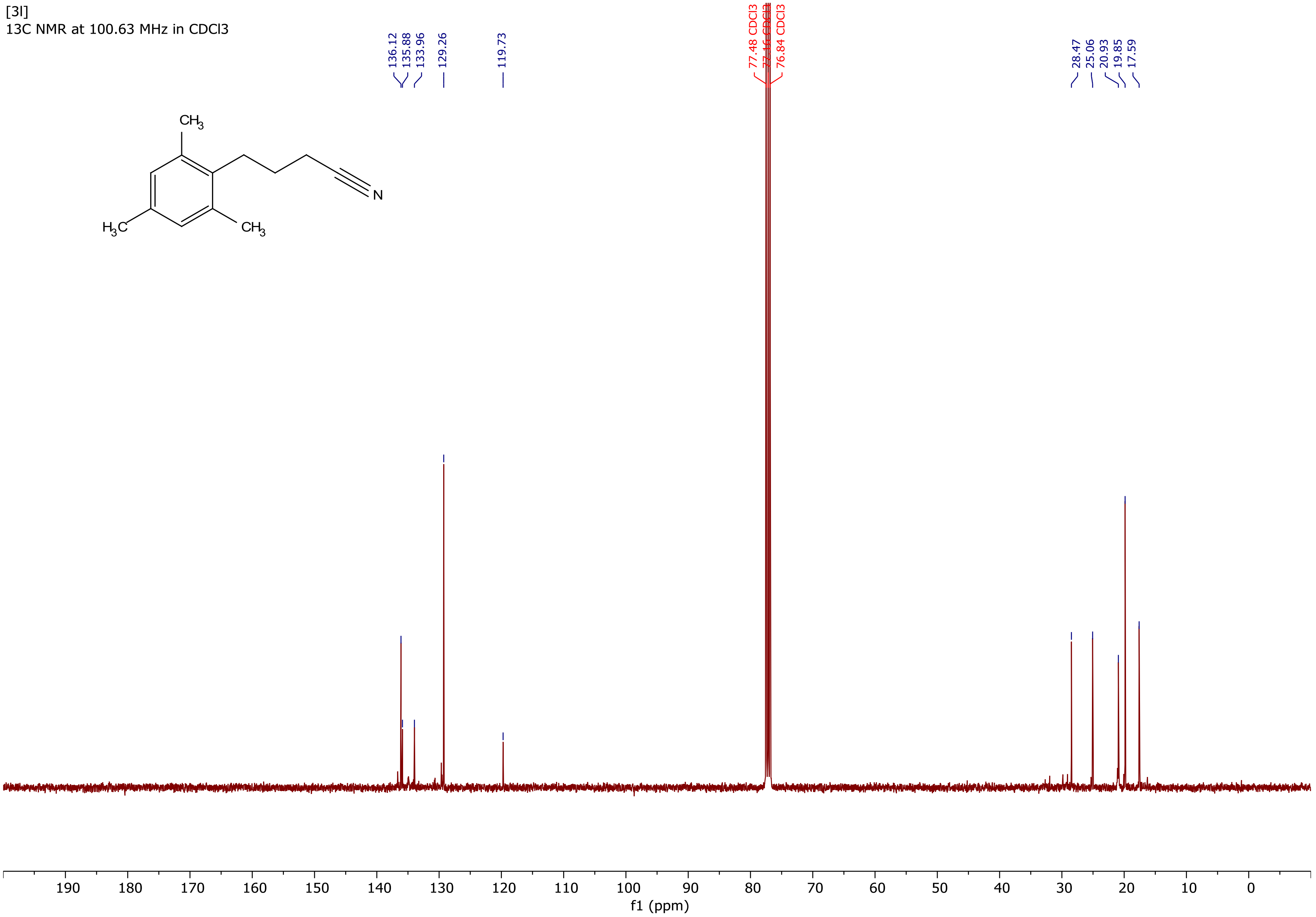
[3l]
13C NMR at 100.63 MHz in CDCl3
136.12
135.88
133.96
129.26
119.73
77.48 CDCl3
76.84 CDCl3
28.47
25.06
20.93
19.85
17.59
CH3
H3C
CH3
N
f1 (ppm)

[3m]
1H NMR at 400.15 MHz in CDCl3

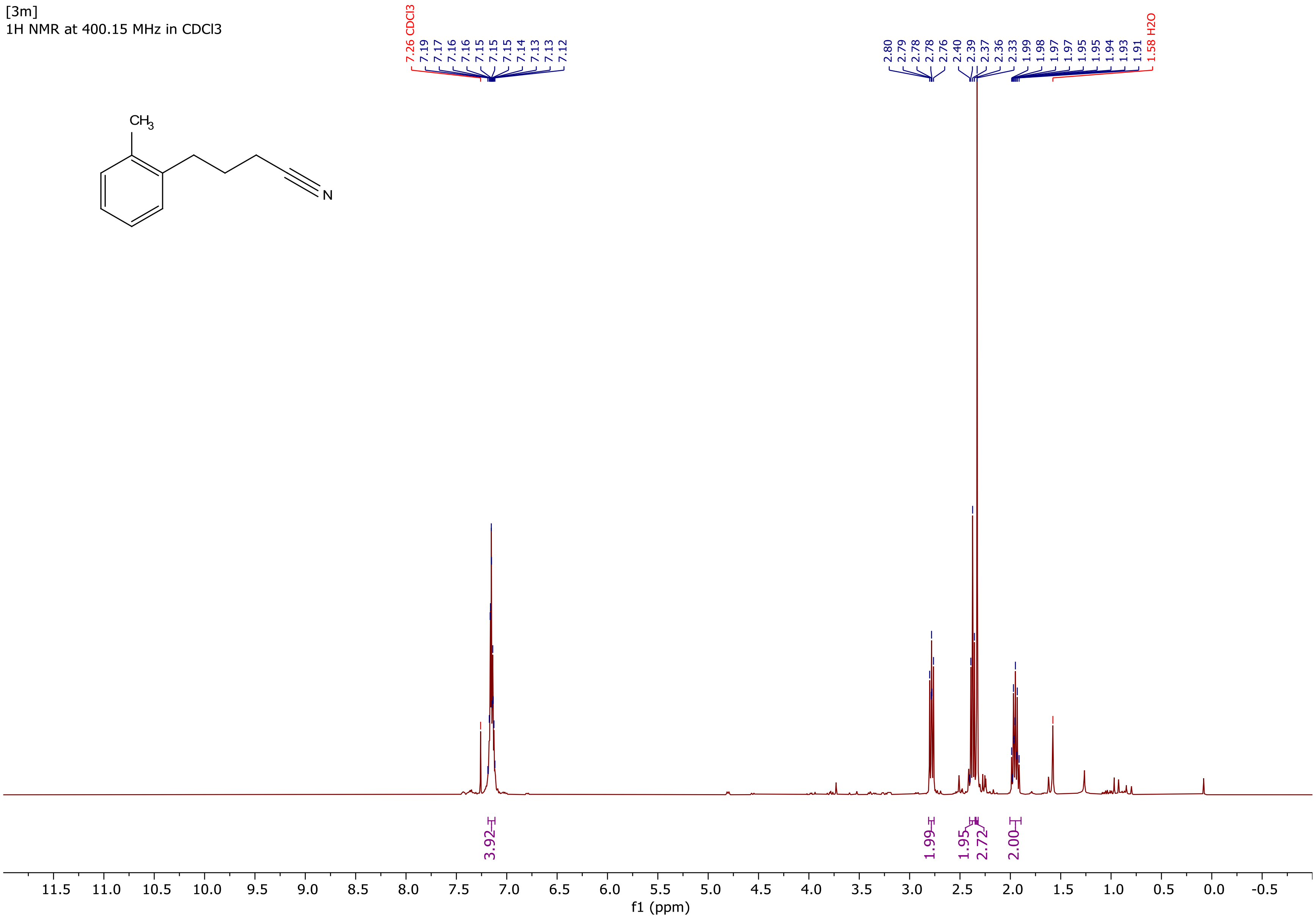

[3m]
13C NMR at 100.63 MHz in CDCl3

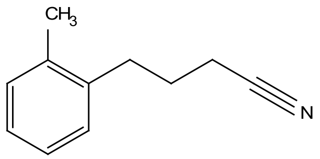

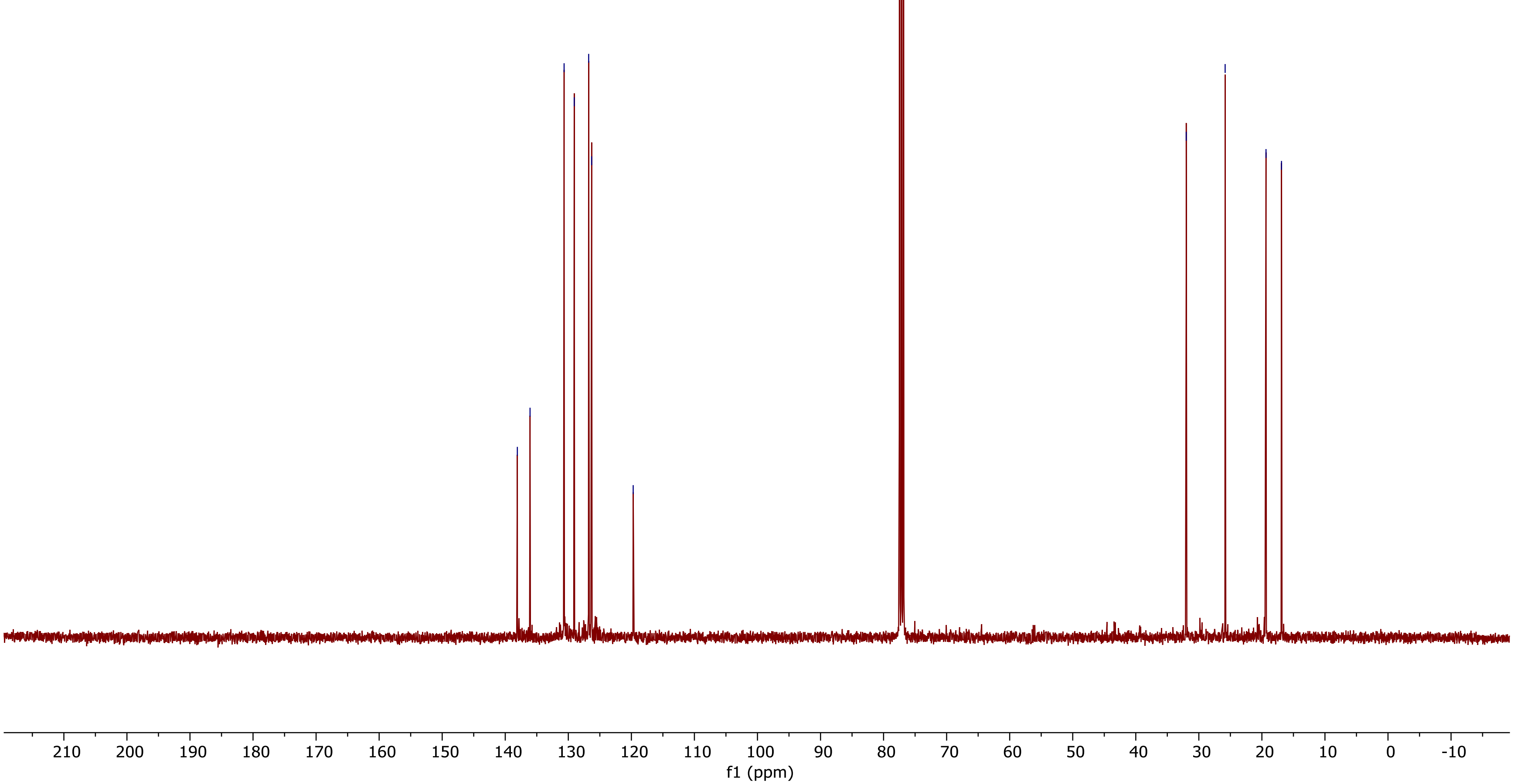

[3n]
1H NMR at 400.15 MHz in CDCl3

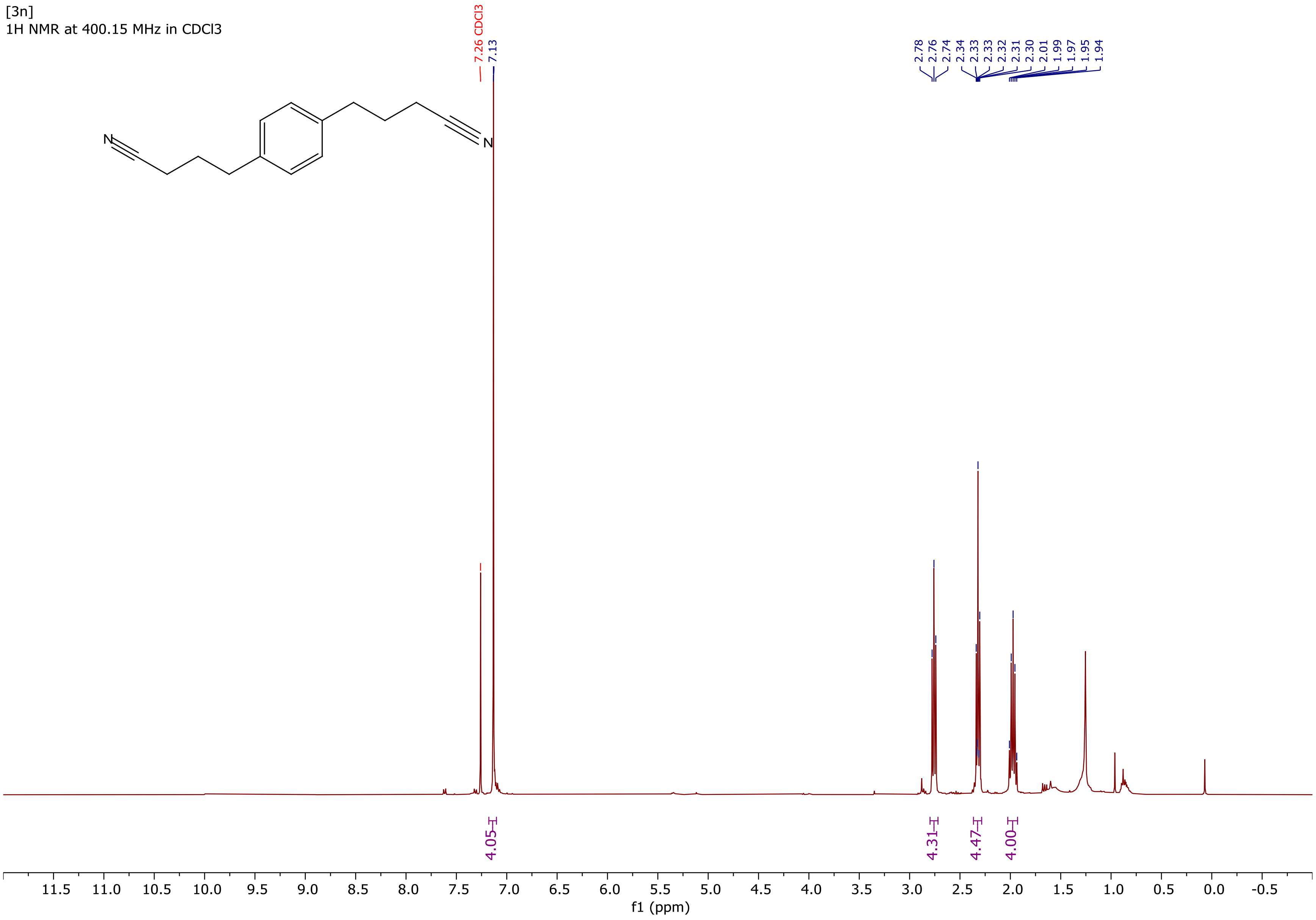

[3n]
13C NMR at 201.27 MHz in CDCl3

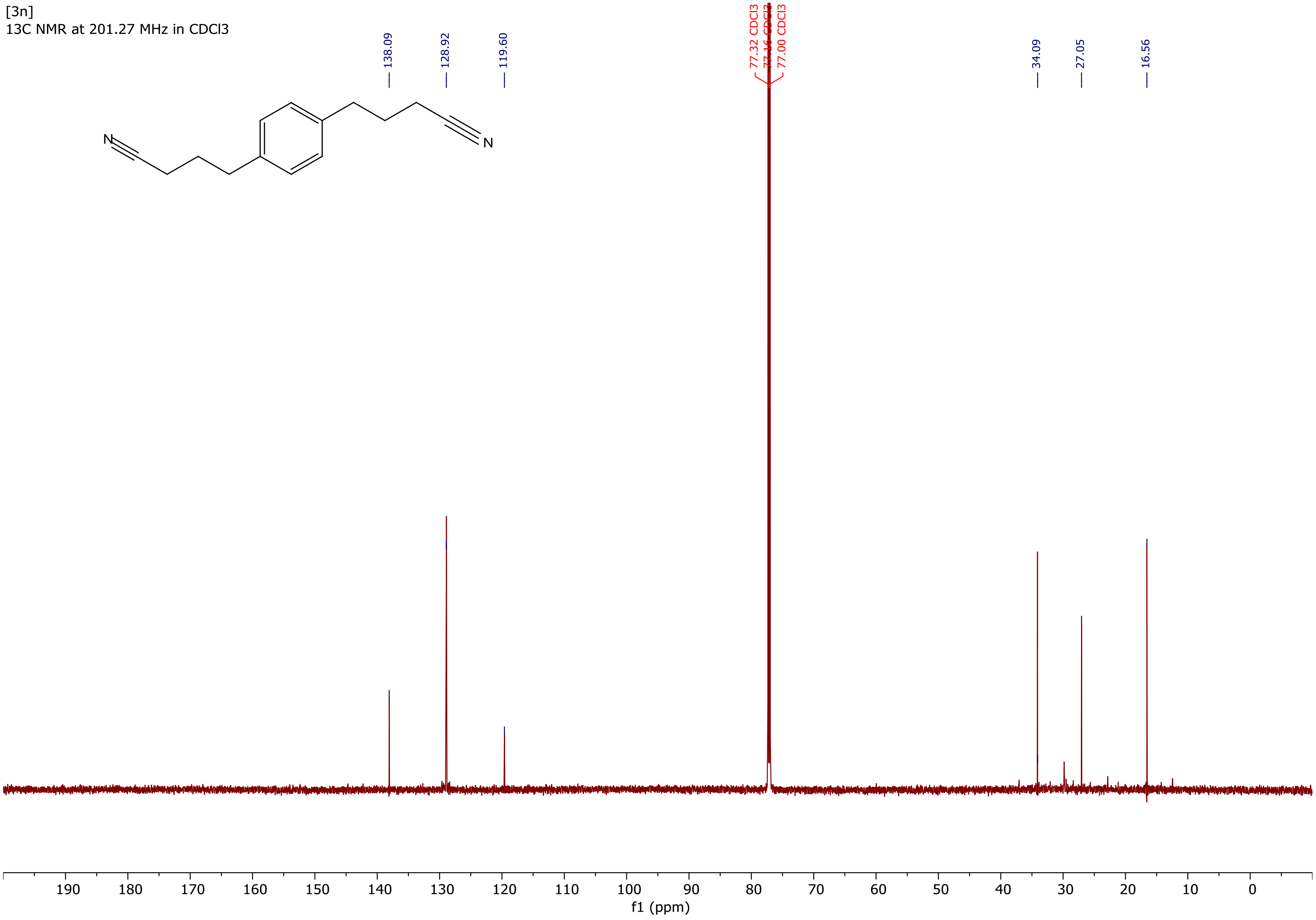

[3o]
1H NMR at 400.15 MHz in CDCl3

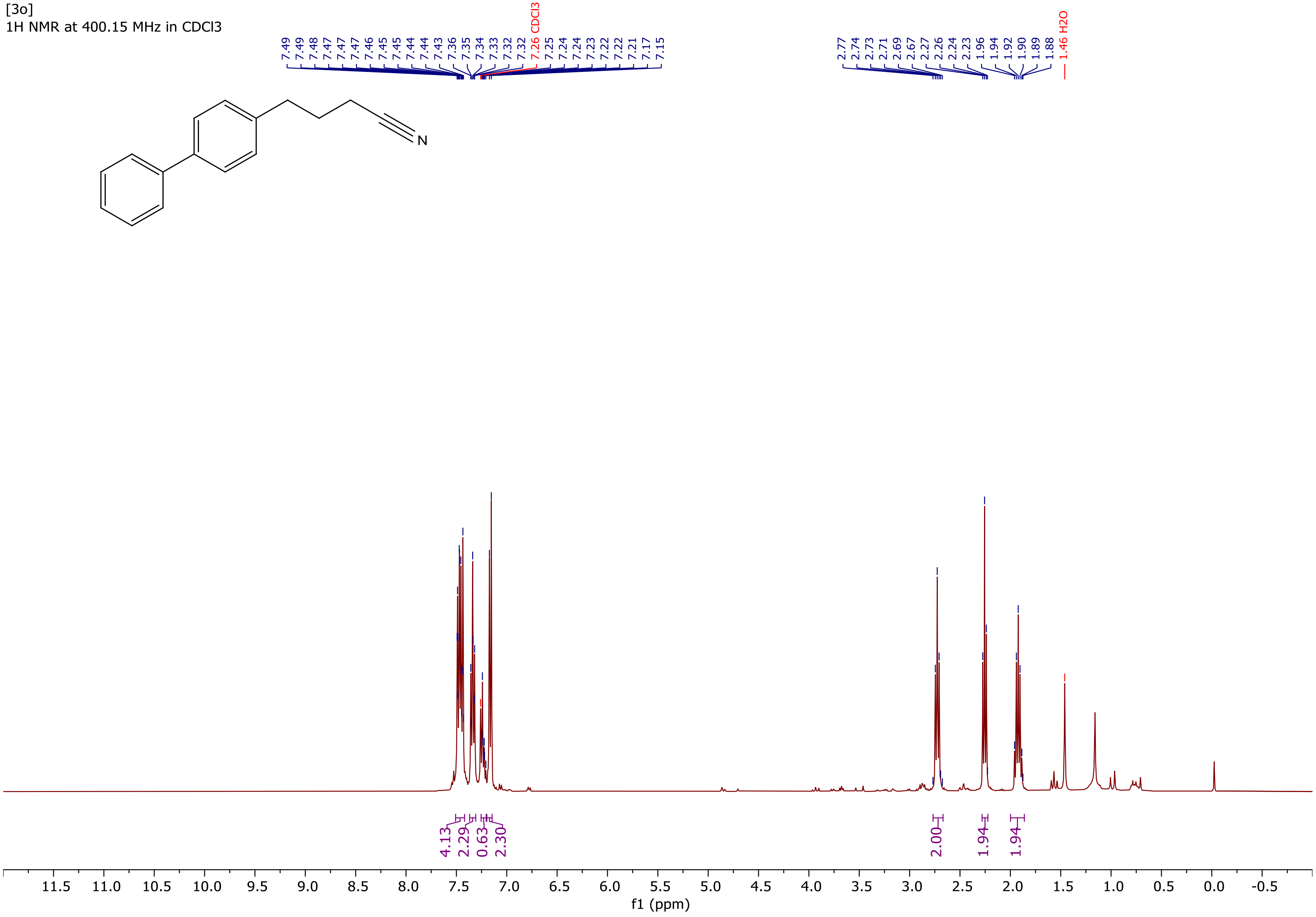

[3o]
13C NMR at 201.27 MHz in CDCl3

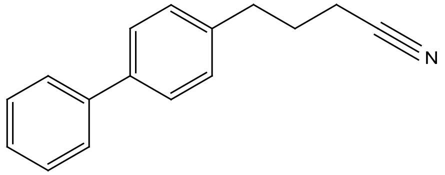

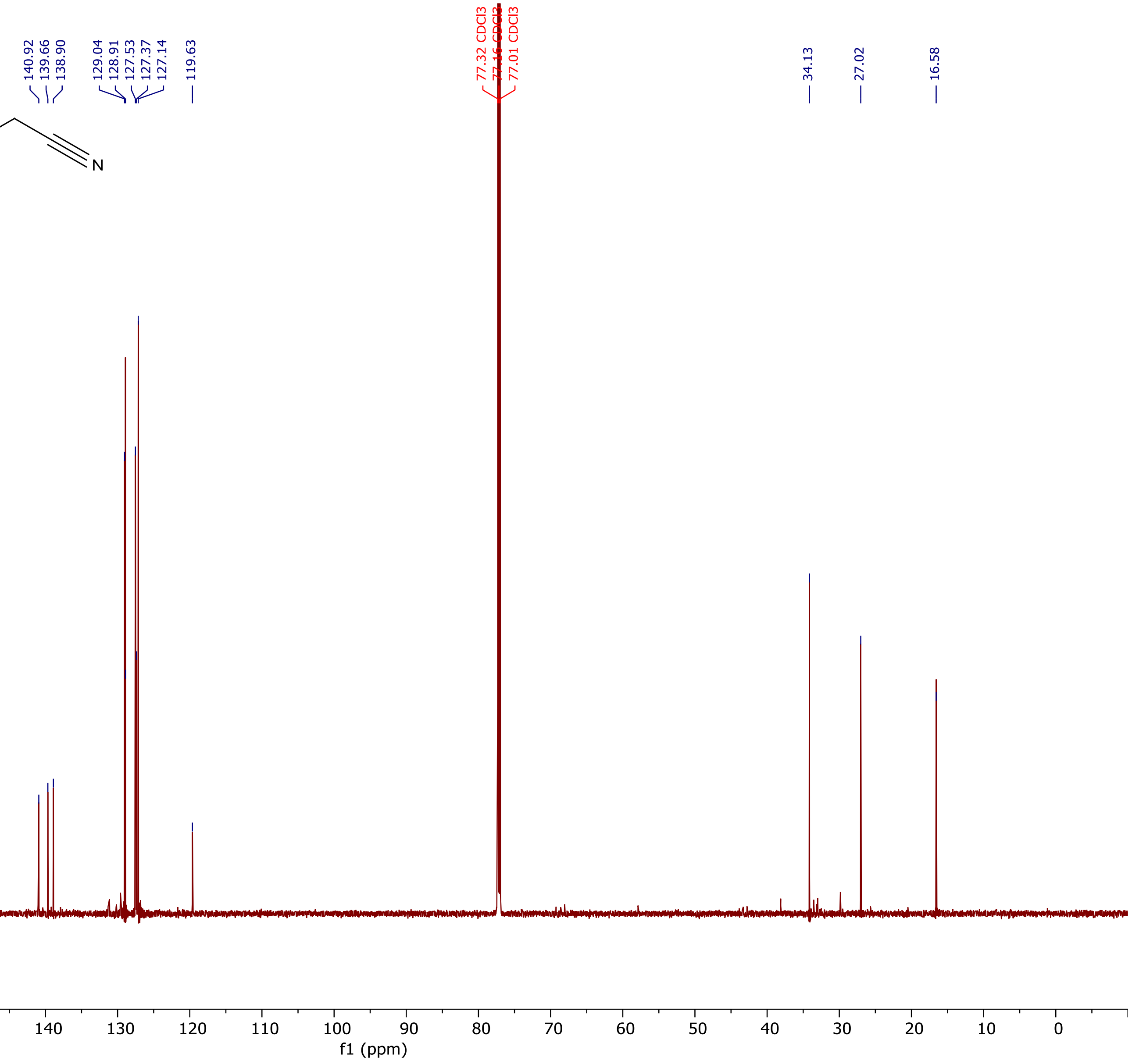

[3p]
1H NMR at 400.15 MHz in CDCl3

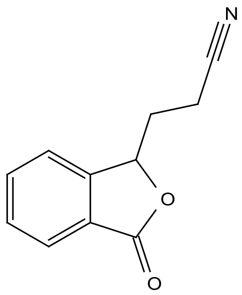

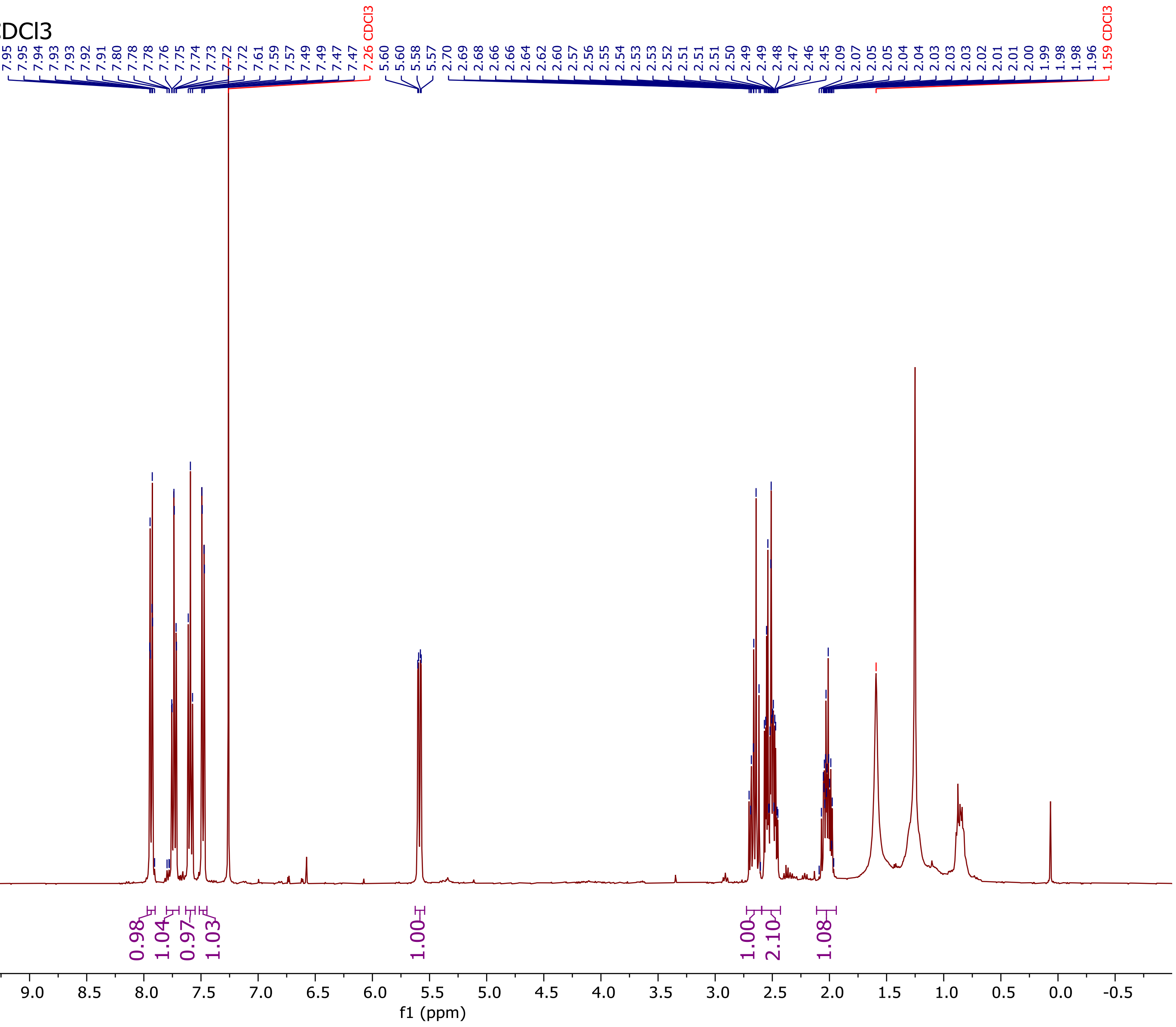

[3p]
13C NMR at 201.27 MHz in CDCl3

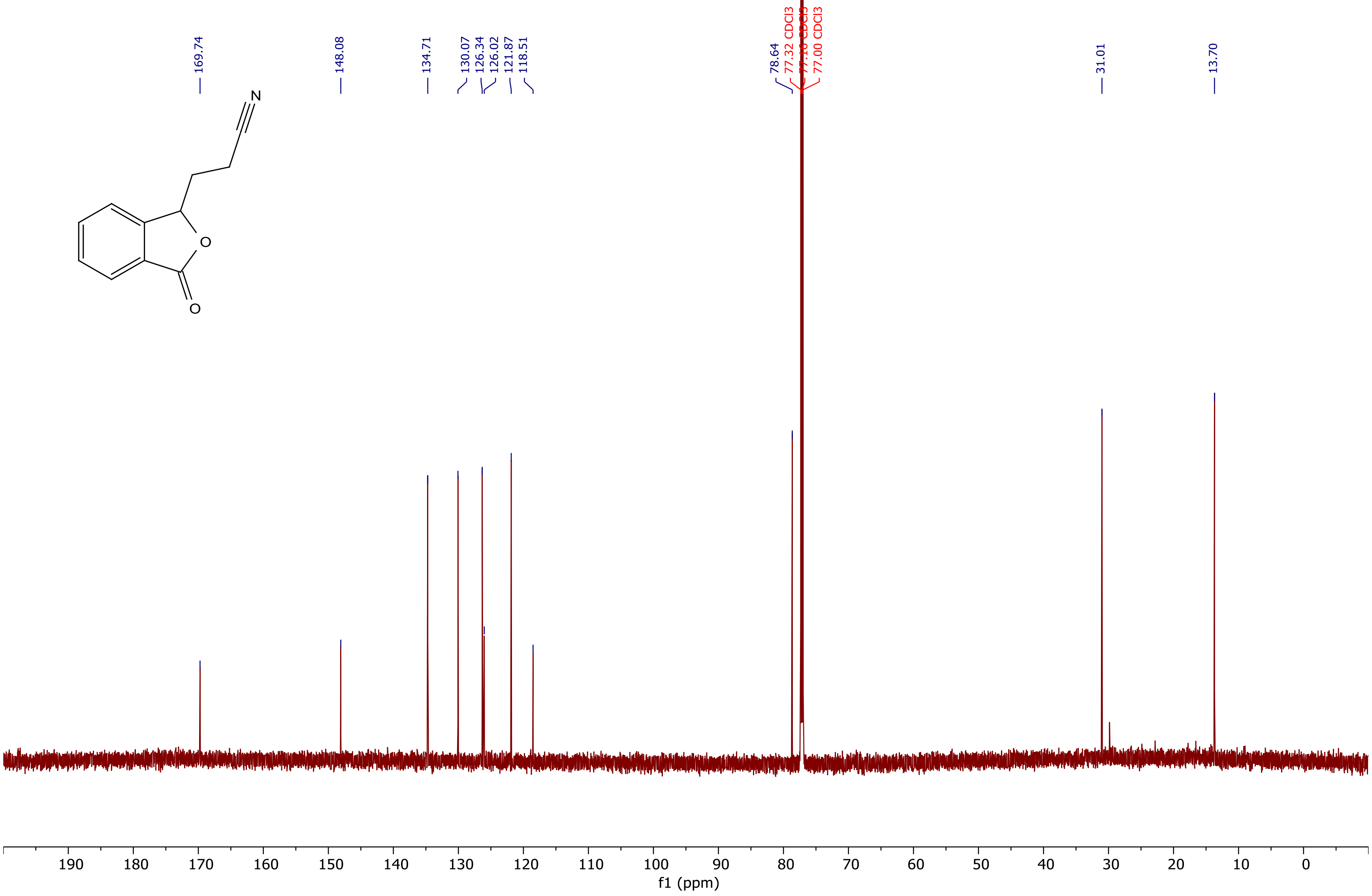

[3q]
1H NMR at 800.34 MHz in CDCl3

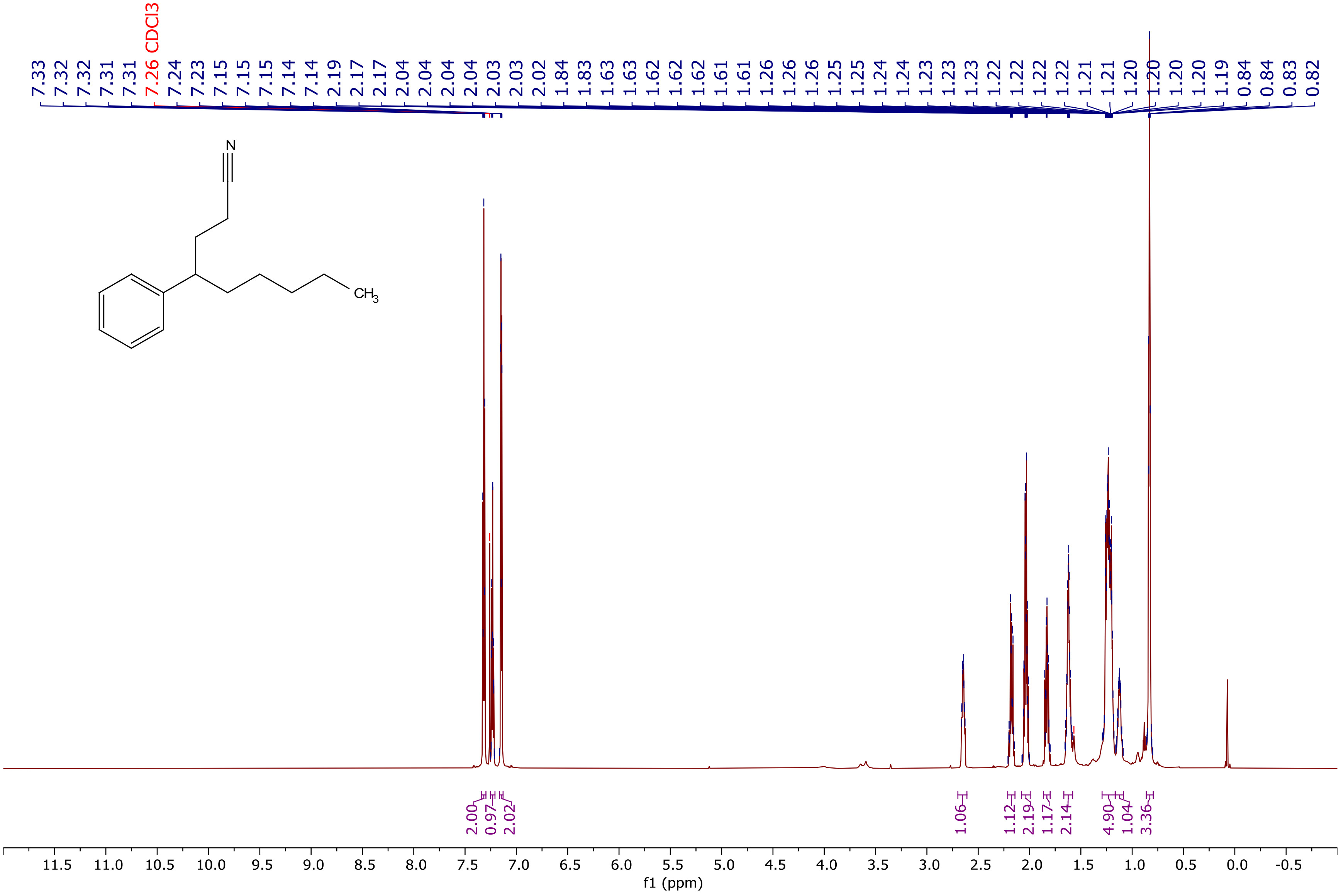

[3q]
13C NMR at 201.27 MHz in CDCl3

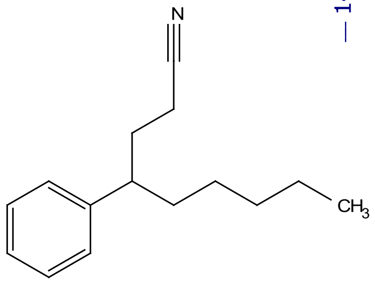

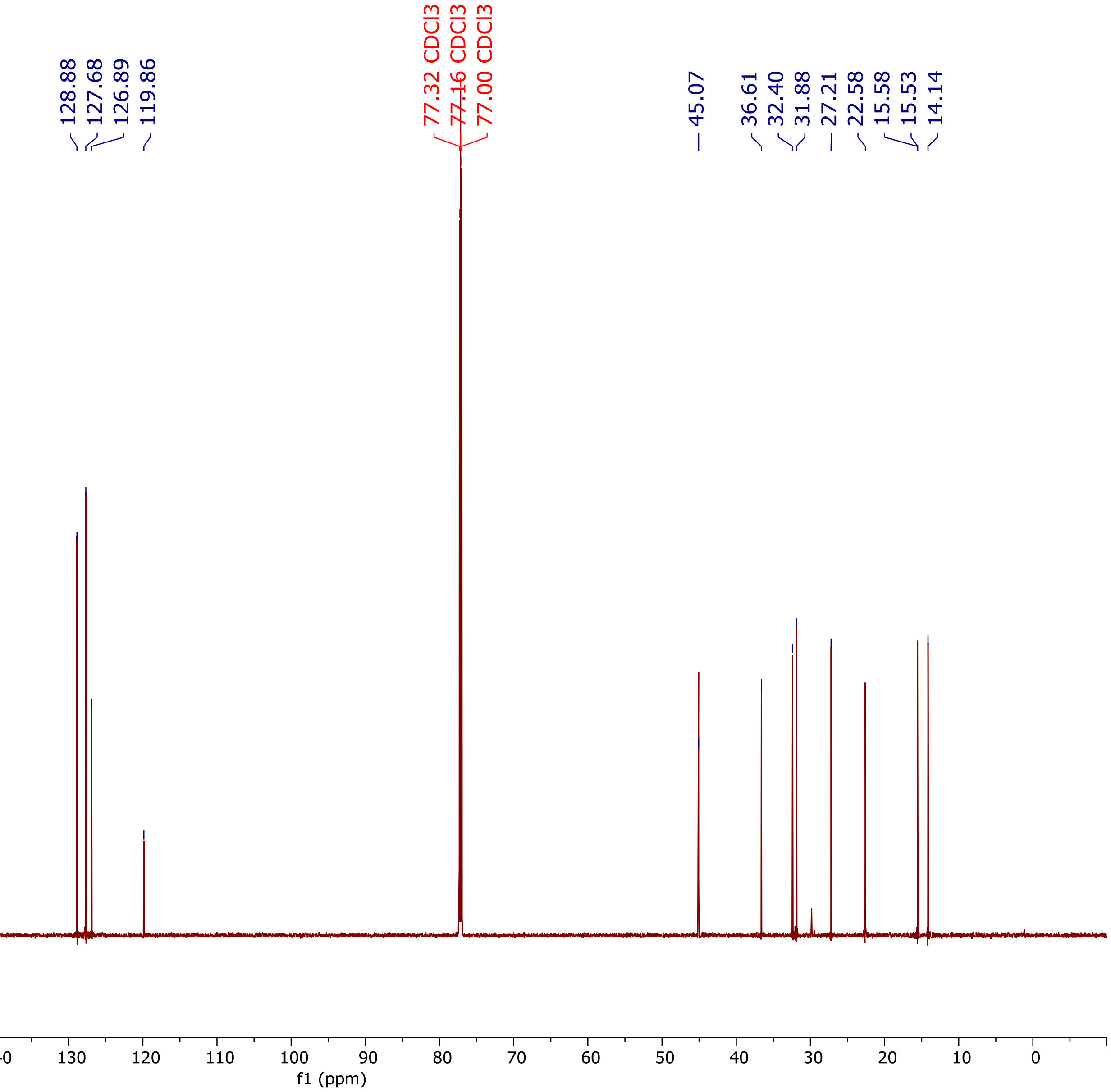

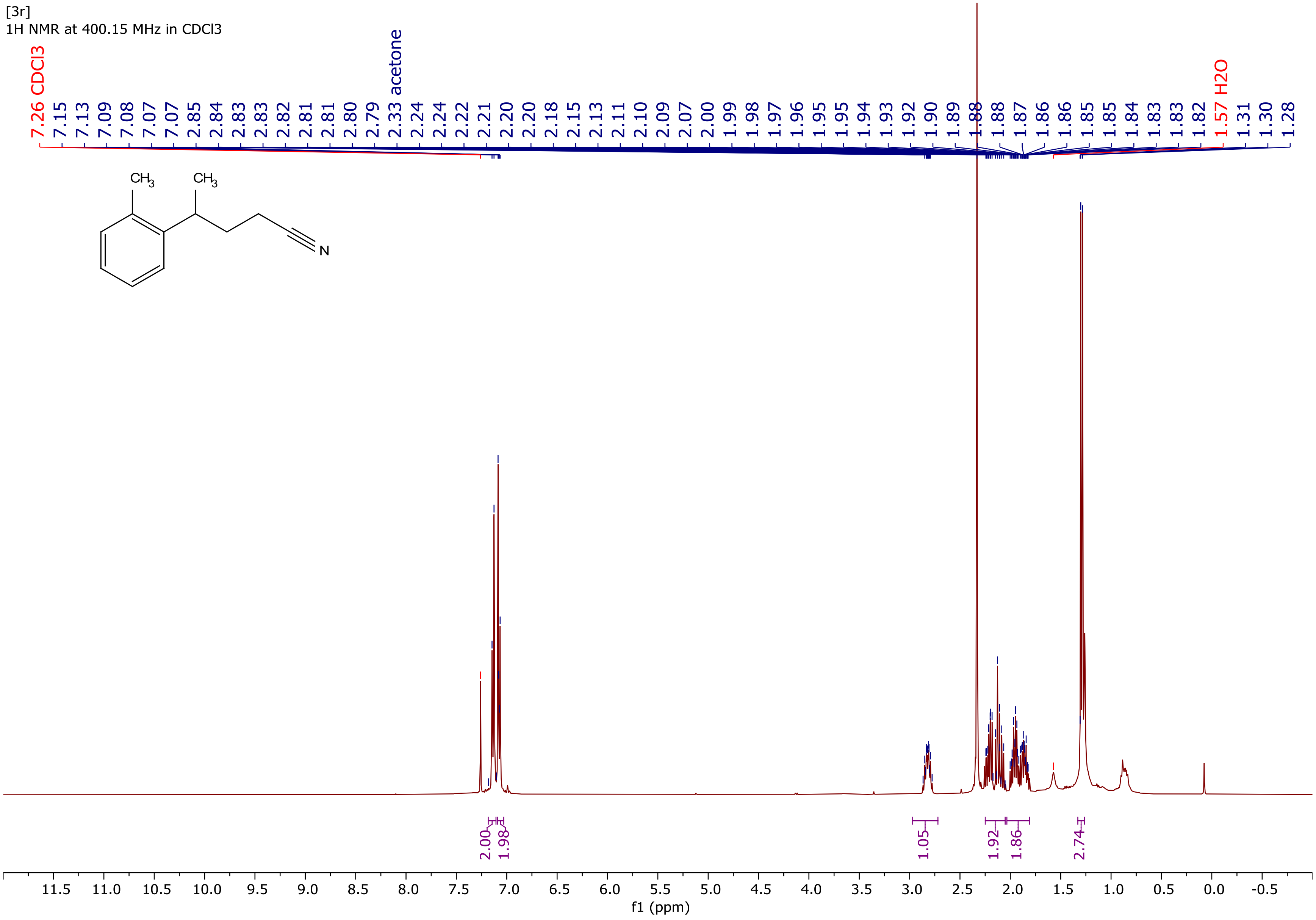
[3r]
1H NMR at 400.15 MHz in CDCl3
7.26 CDCl3
7.15
7.13
7.09
7.08
7.07
7.07
2.85
2.84
2.83
2.83
2.82
2.81
2.81
2.80
2.79
2.33 acetone
2.24
2.24
2.22
2.21
2.20
2.20
2.18
2.15
2.13
2.11
2.10
2.09
2.07
2.00
1.99
1.98
1.97
1.96
1.95
1.95
1.94
1.93
1.92
1.90
1.89
1.88
1.88
1.87
1.86
1.86
1.85
1.85
1.84
1.83
1.83
1.82
1.57 H2O
1.31
1.30
1.28
CH3
CH3
N
2.00
1.98
1.05
1.92
1.86
2.74
f1 (ppm)

[3r]
13C NMR at 201.27 MHz in CDCl3

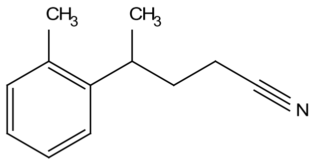

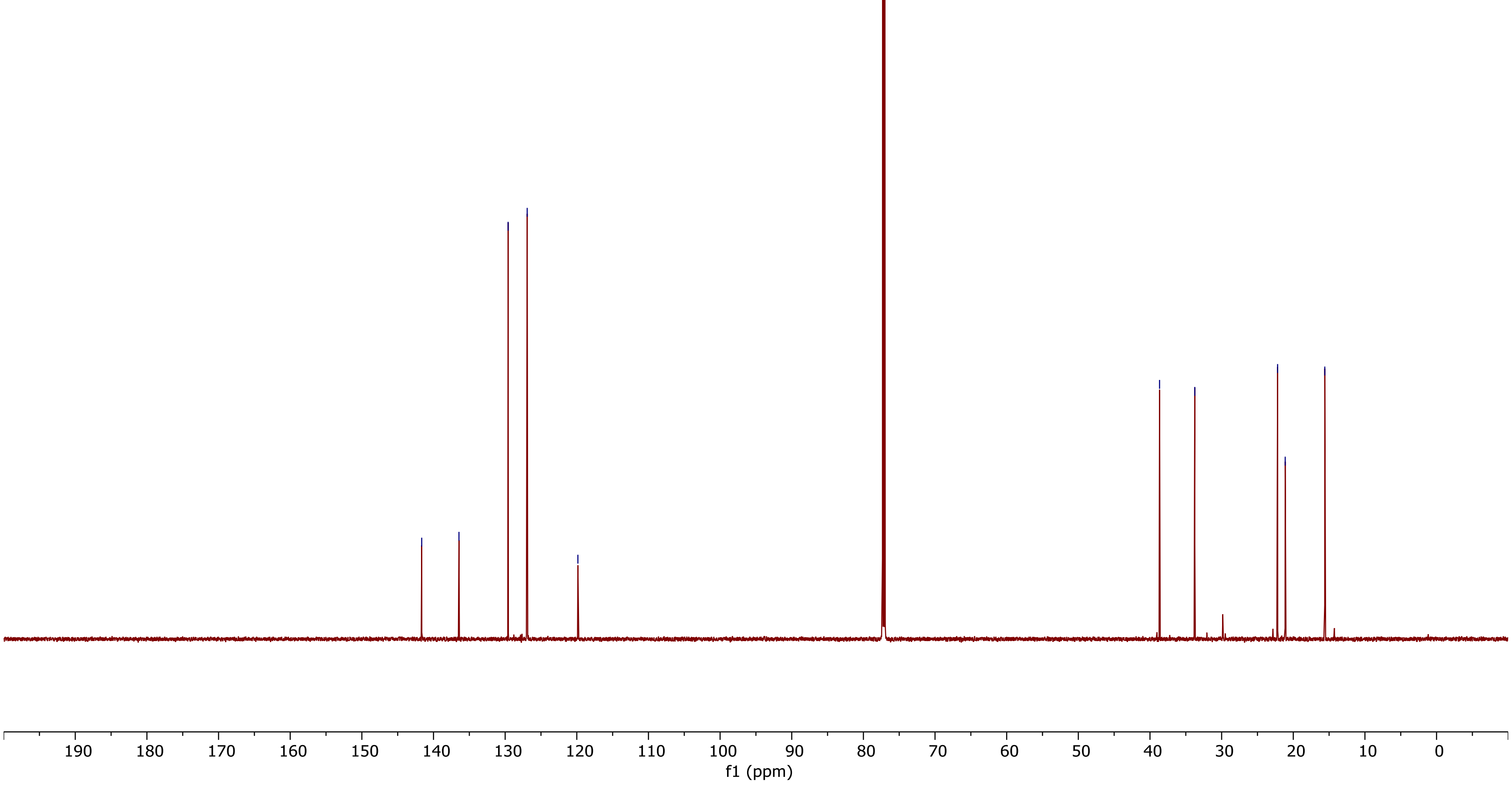

[3s]
1H NMR at 400.15 MHz in CDCl3

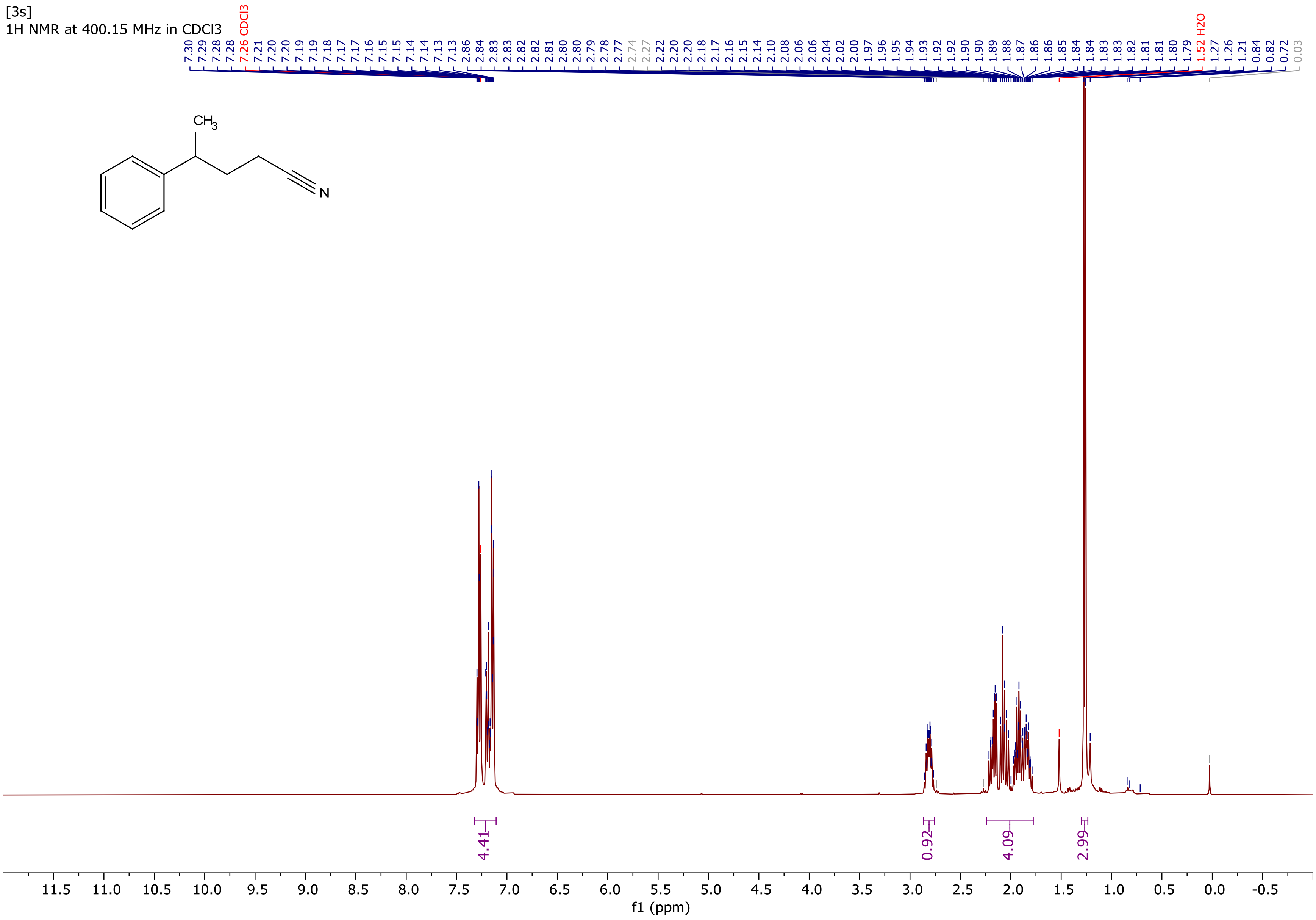

[3s]
13C NMR at 201.27 MHz in CDCl3

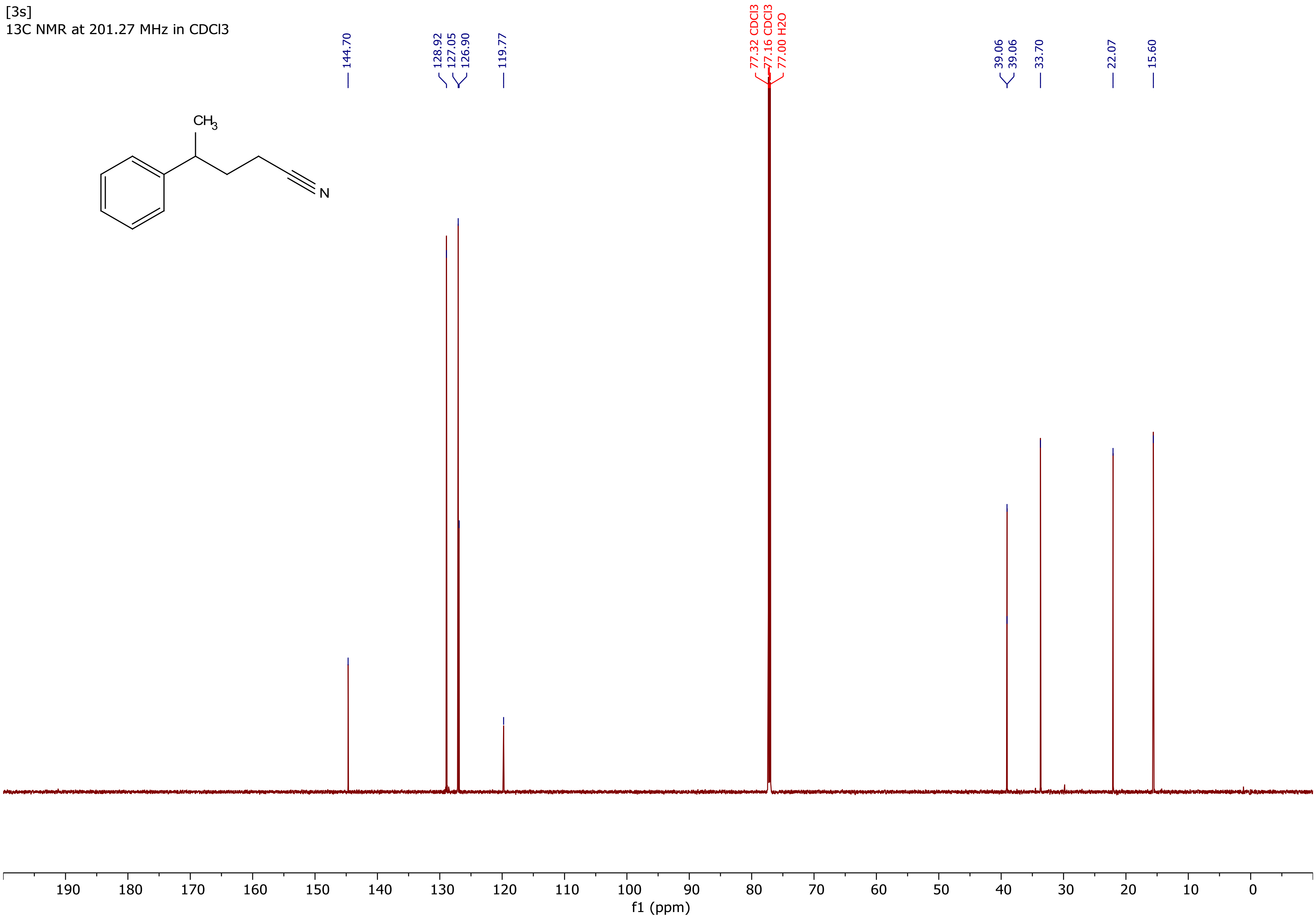

[3t]
1H NMR at 800.34 MHz in CDCl3

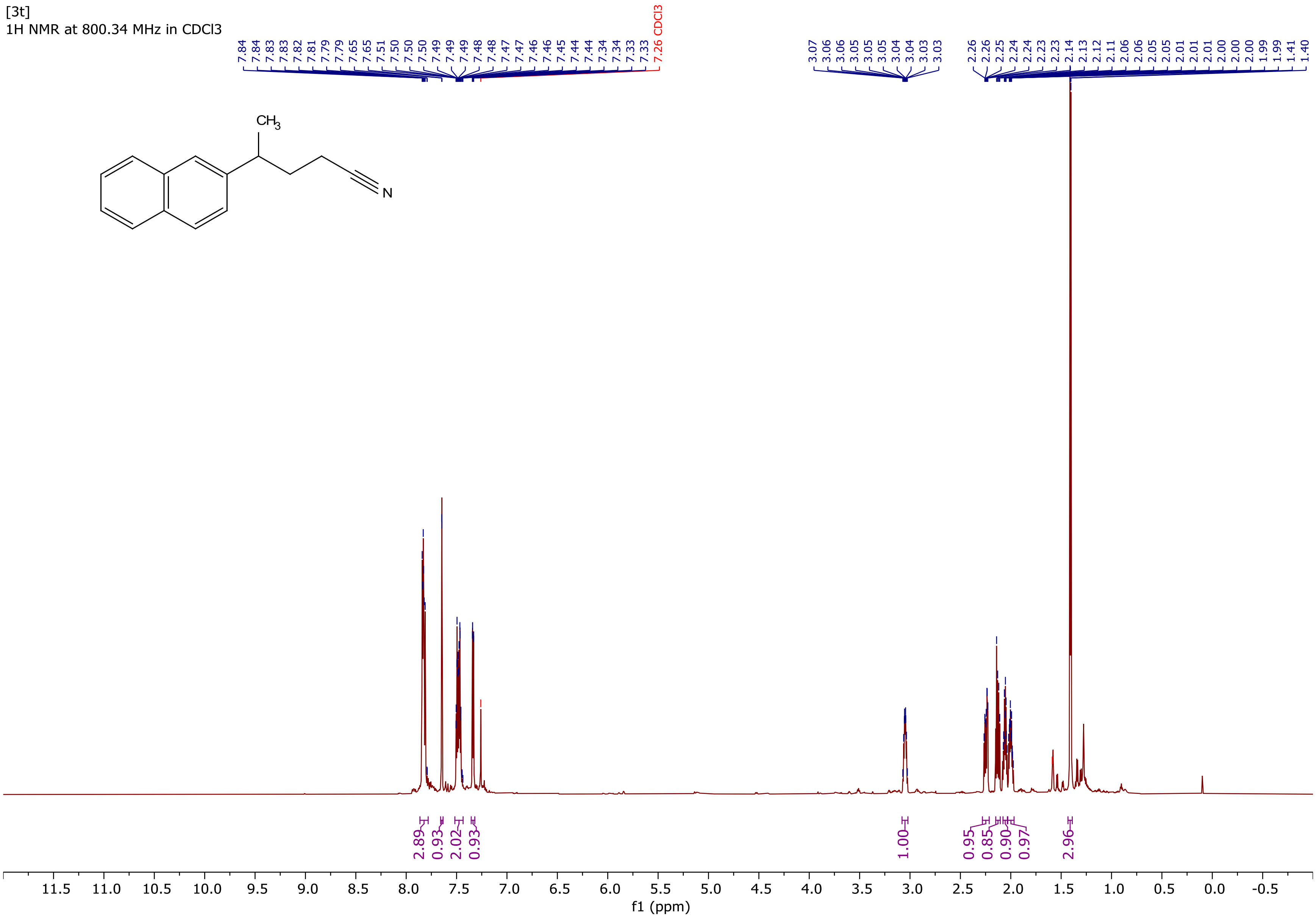

[3t]
13C NMR at 201.27 MHz in CDCl3

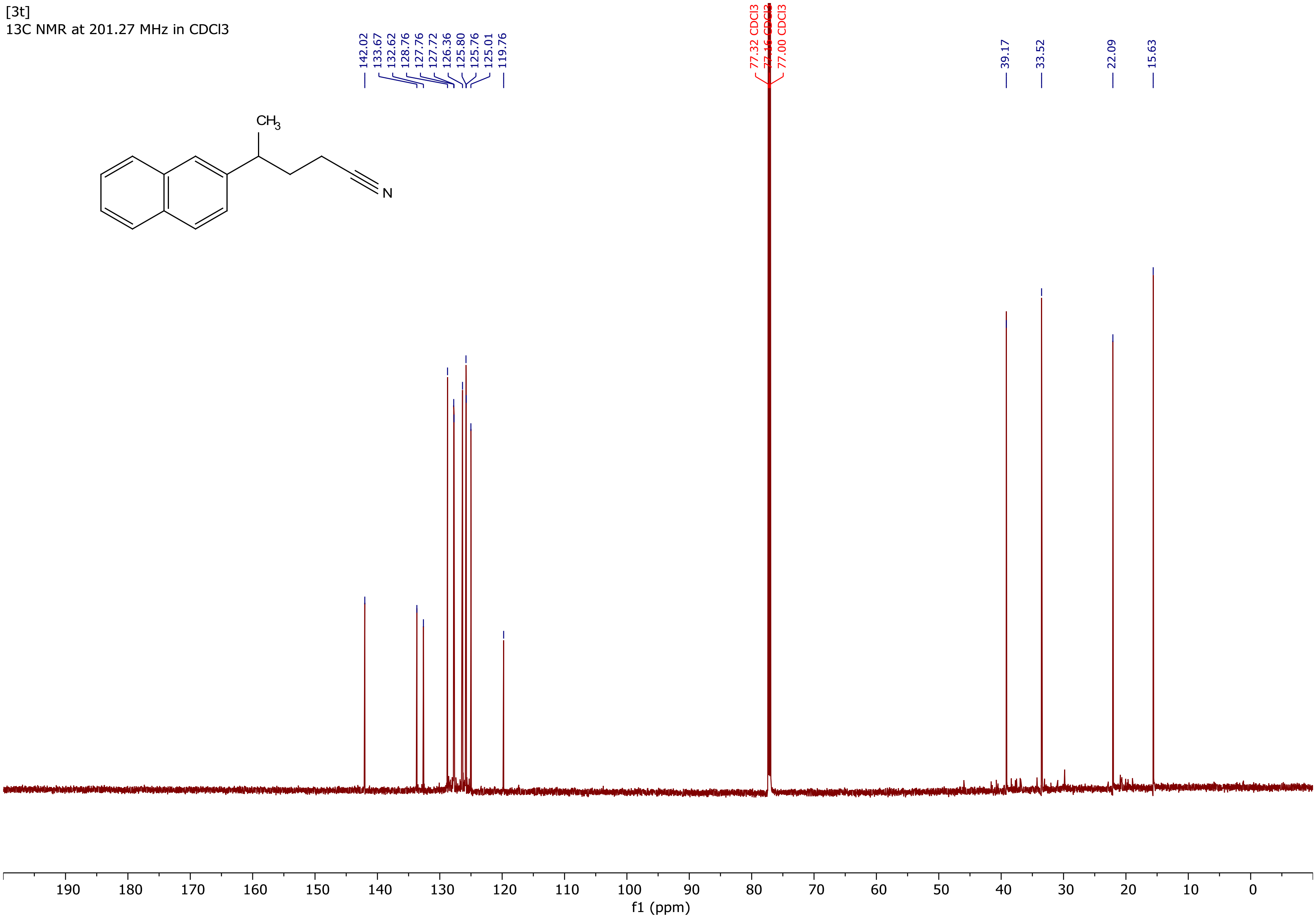

[3u]
1H NMR at 400.15 MHz in CDCl3

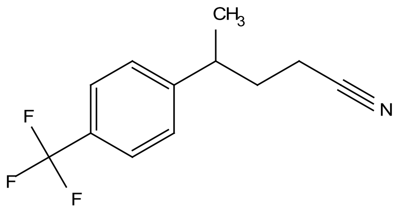

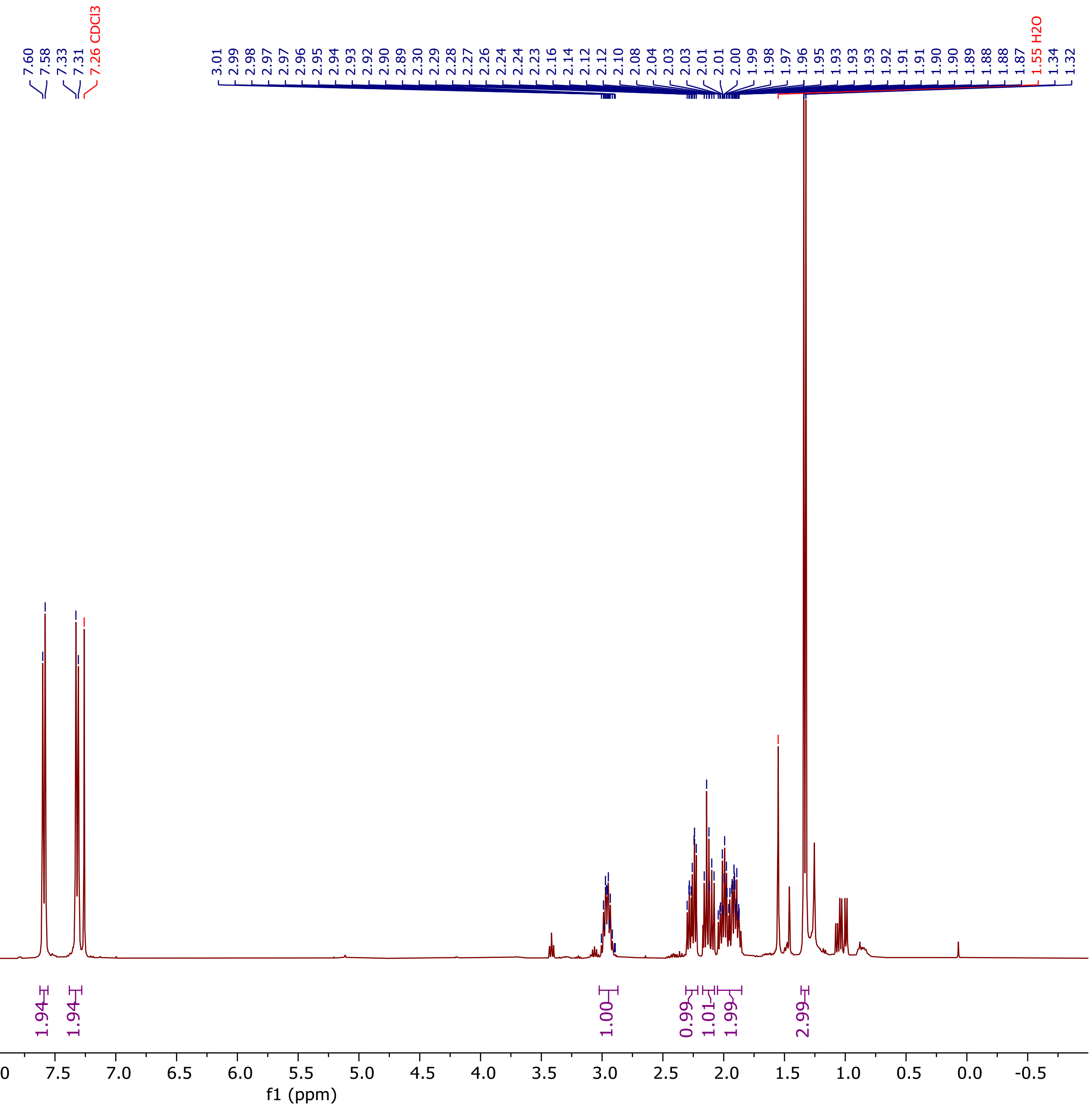

[3u]
13C NMR at 201.27 MHz in CDCl3

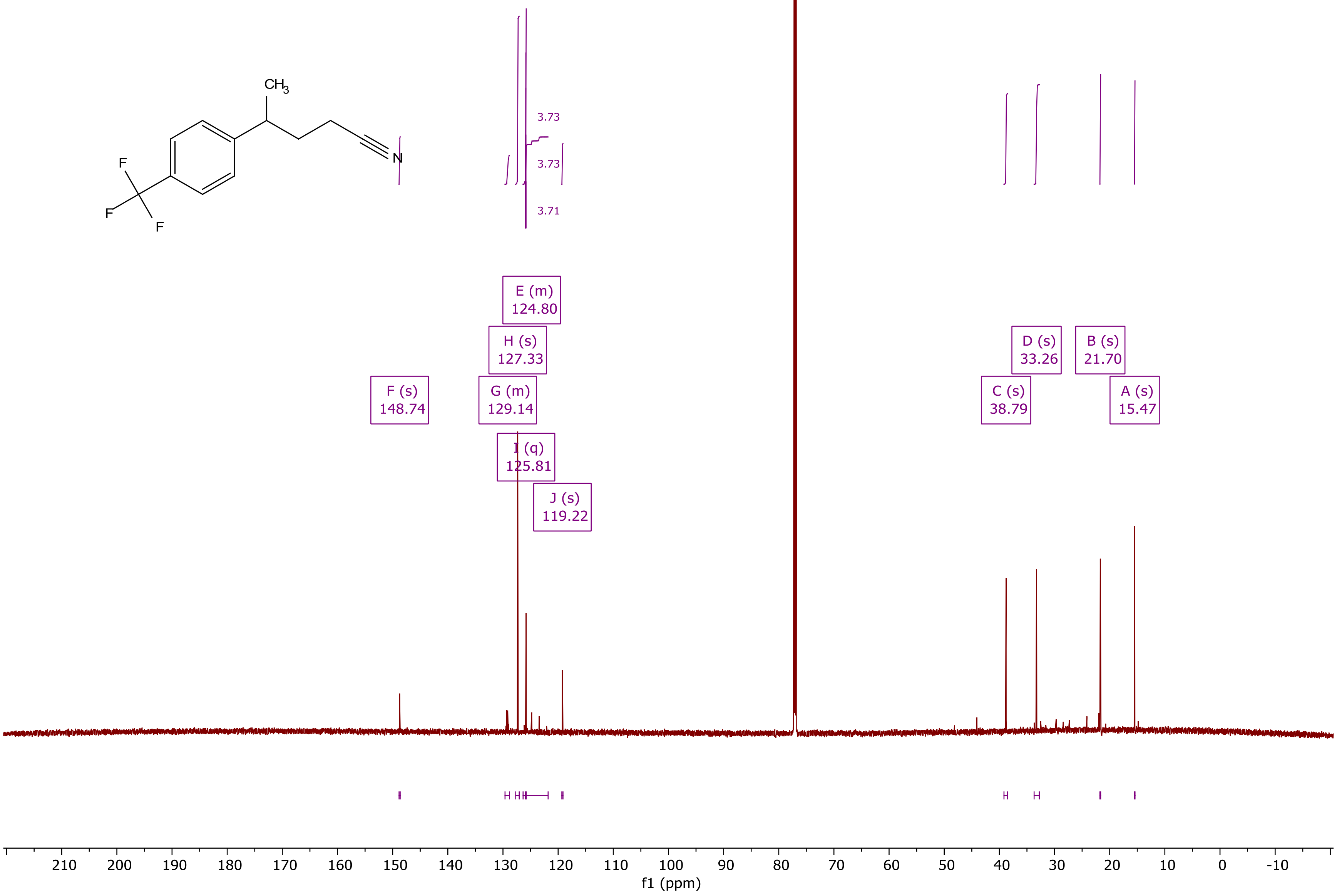

[3u]
19F NMR at 376.48 MHz in CDCl3

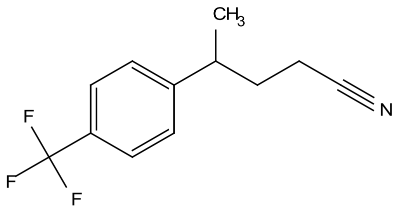

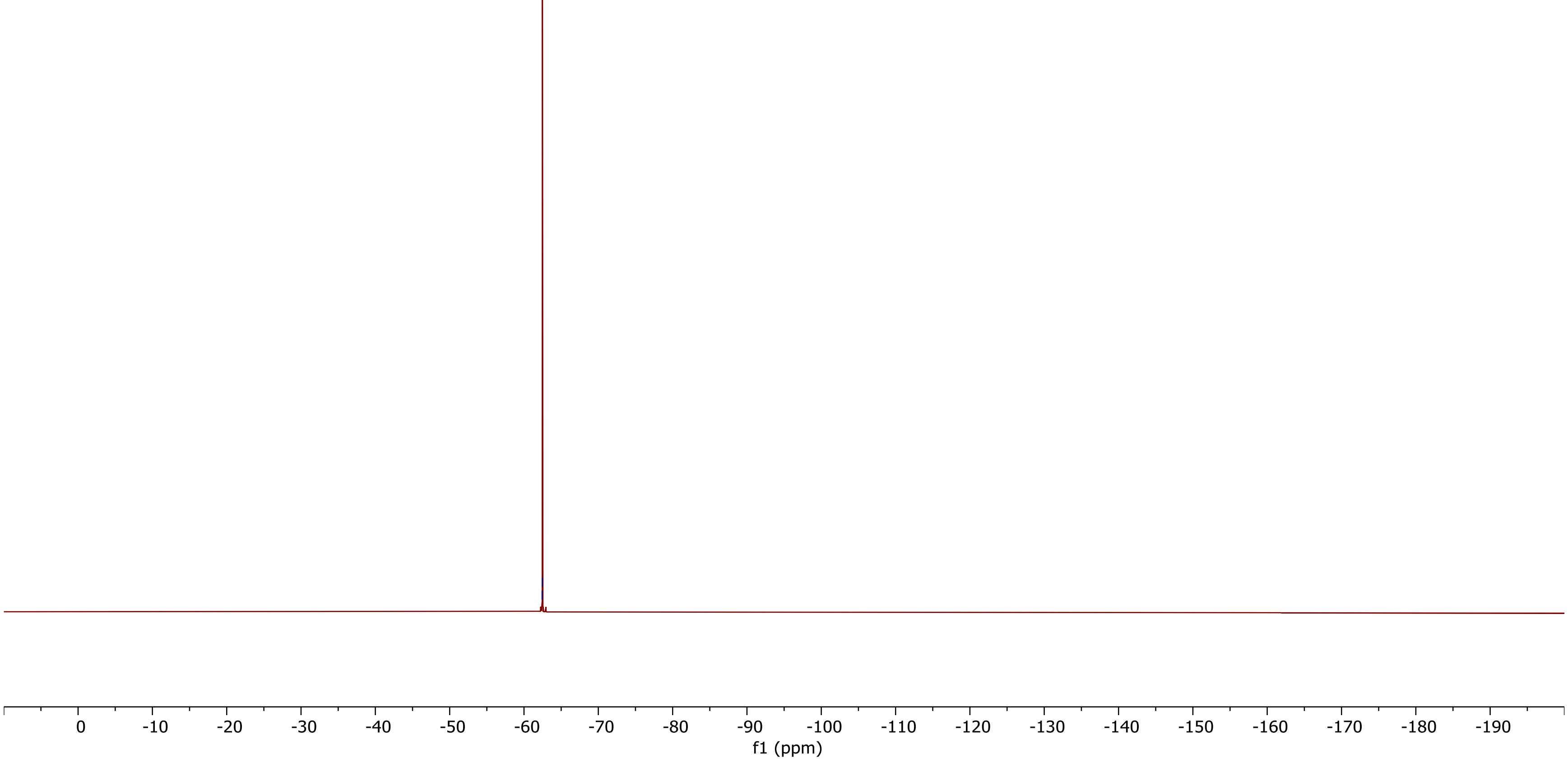

[3v]
1H NMR at 400.15 MHz in CDCl3

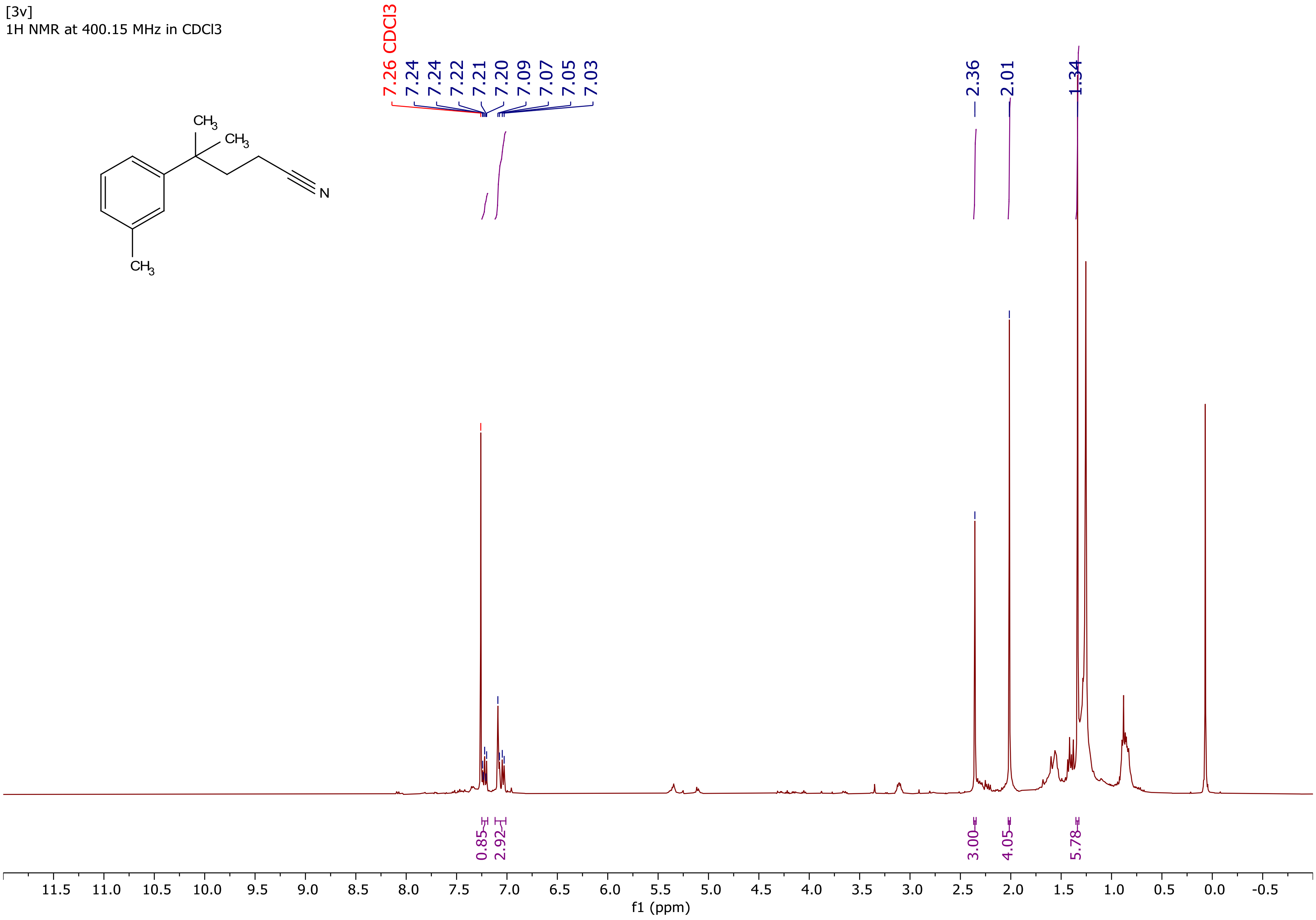

[3v]
13C NMR at 100.63 MHz in CDCl3

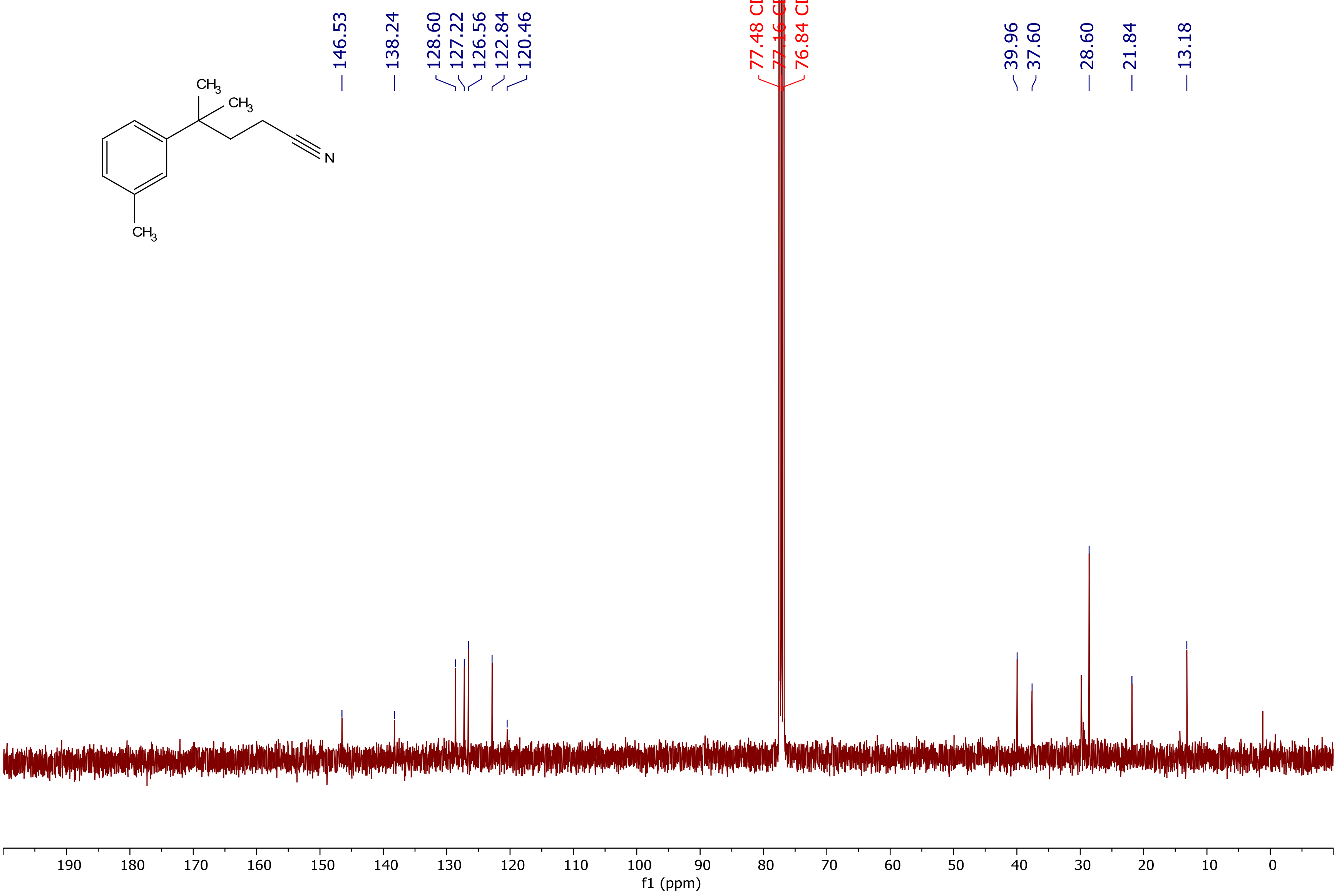

[3w]
1H NMR at 400.15 MHz in CDCl3

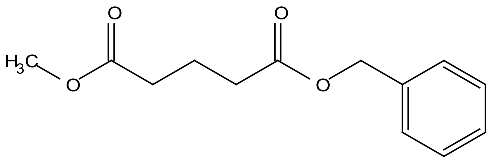

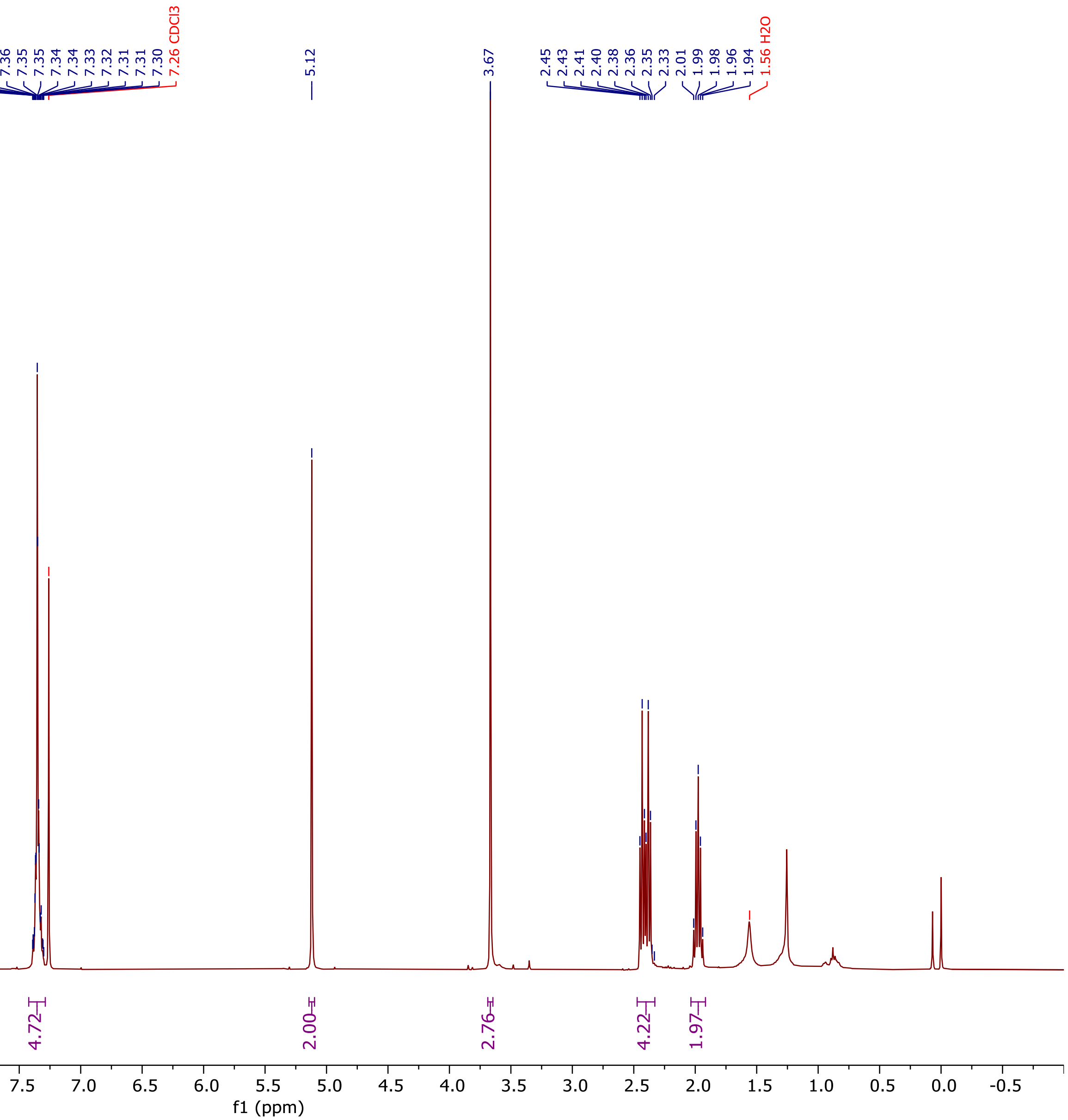

[3w]
13C NMR at 100.63 MHz in CDCl3

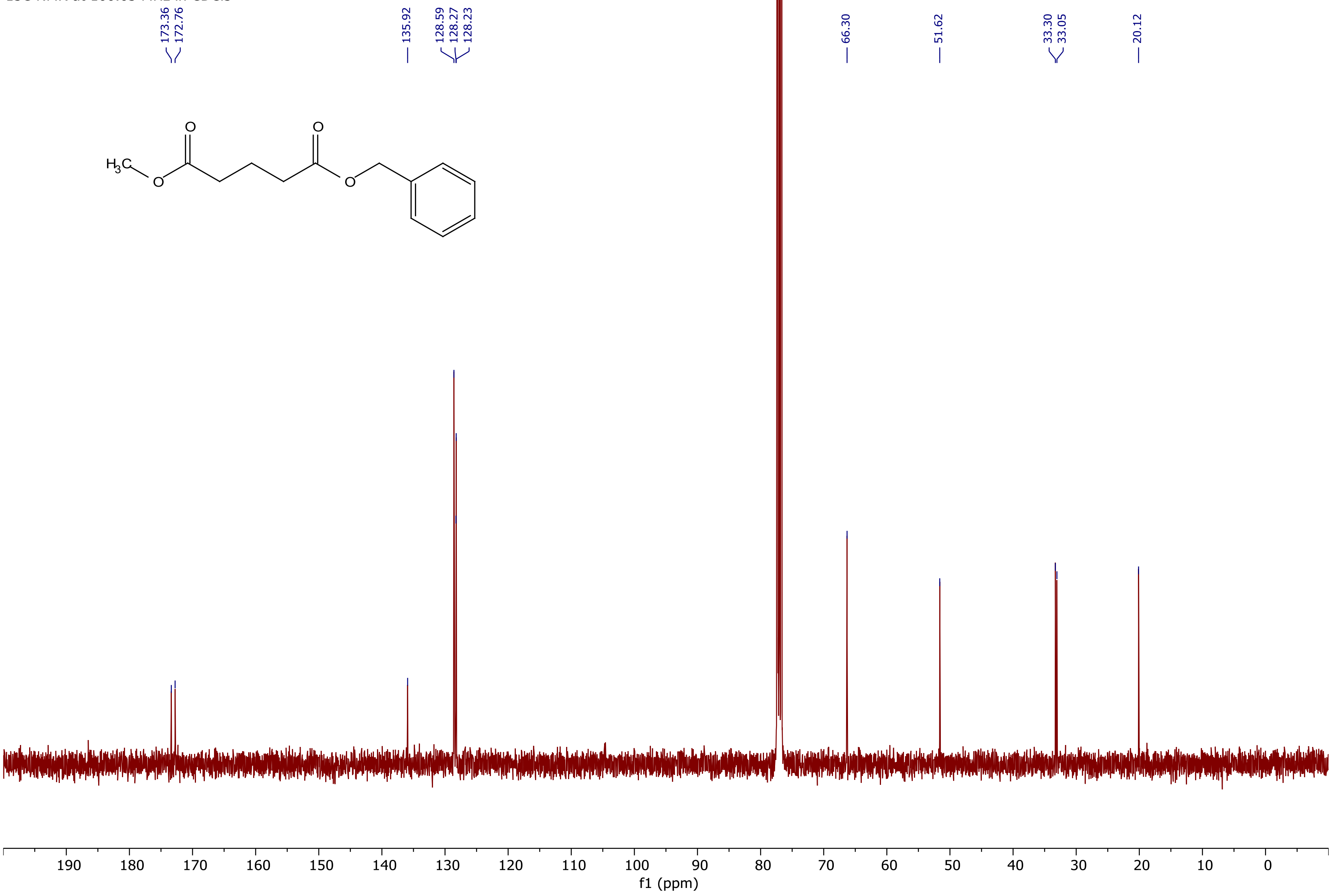

Supplement: Supplementary file 1 — ol4c01413_si_001.pdf [file ol4c01413_si_001.pdf]
