# Supplementary material for: Cooperative Catalytic Coupling of Benzyl Chlorides and Bromides with Electron-Deficient Alkenes
Source: Org Lett. 2024 Jun 19;26(25):5248–52. doi: 10.1021/acs.orglett.4c01413 (PMC11217938; doi:10.1021/acs.orglett.4c01413)
Supplement: Supplementary file 2 — ol4c01413_si_002.zip [file ol4c01413_si_002.zip › FID for publication/1a/Primary_NMR_Data_files/1H/pdata/1/email_RH-3056-4_11_1.pdf]

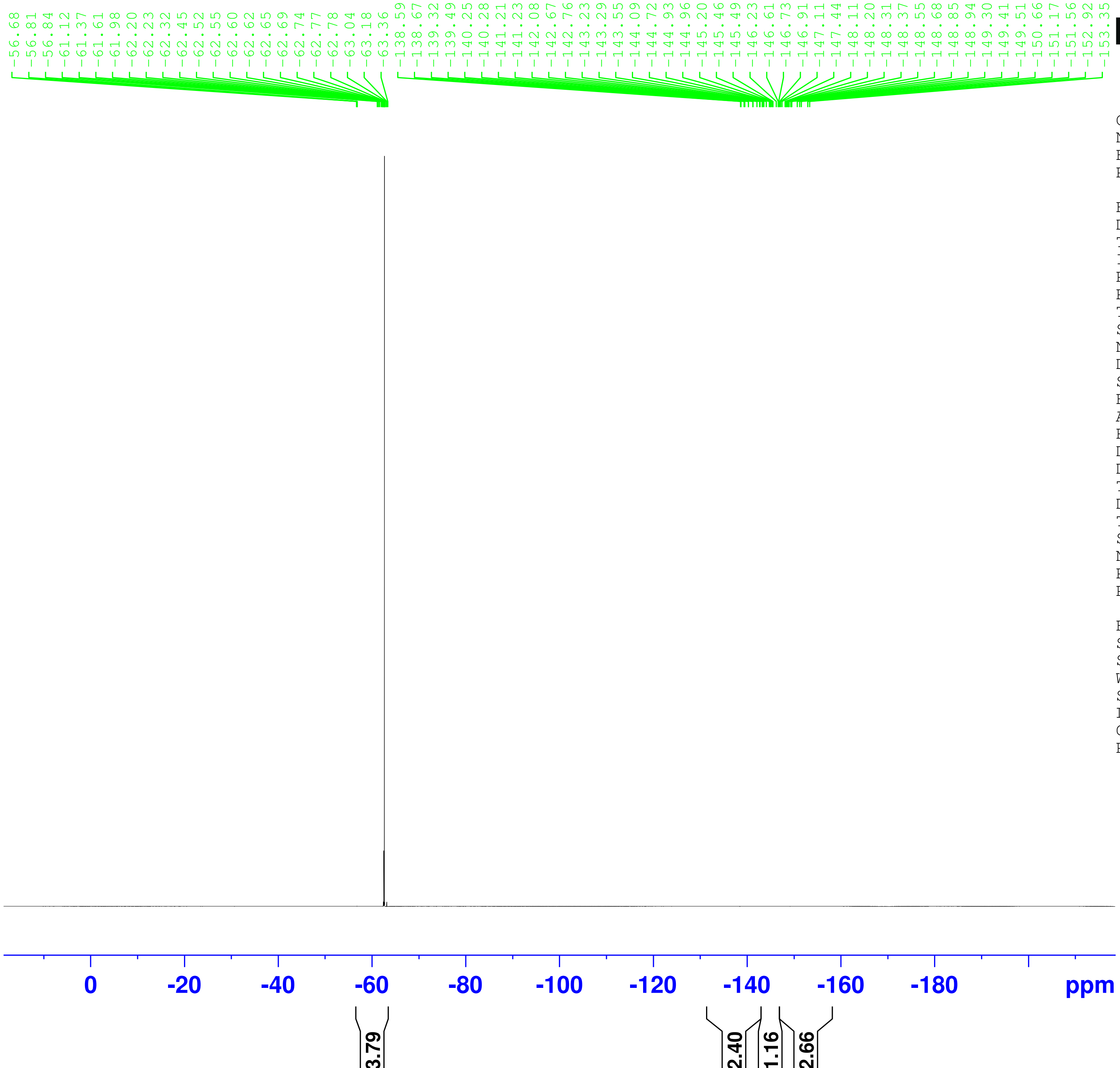


Current Data Parameters

| | |
|---|---|
| NAME | RH-3056-4 |
| EXPNO | 11 |
| PROCNO | 1 |

F2 - Acquisition Parameters

| | |
|---|---|
| Date_ | 20240118 |
| Time | 21.17 h |
| INSTRUM | spect |
| PROBHD | Z116098_0222 ( |
| PULPROG | zgflqn |
| TD | 131072 |
| SOLVENT | CDCl3 |
| NS | 16 |
| DS | 4 |
| SWH | 89285.711 Hz |
| FIDRES | 1.362392 Hz |
| AQ | 0.7340032 sec |
| RG | 124.73 |
| DW | 5.600 usec |
| DE | 6.50 usec |
| TE | 298.0 K |
| D1 | 1.00000000 sec |
| TD0 | 1 |
| SFO1 | 376.4795333 MHz |
| NUC1 | 19F |
| P1 | 14.00 usec |
| PLW1 | 22.00000000 W |

F2 - Processing parameters

| | |
|---|---|
| SI | 65536 |
| SF | 376.5171850 MHz |
| WDW | EM |
| SSB | 0 |
| LB | 0.30 Hz |
| GB | 0 |
| PC | 1.00 |
