# Supplementary figures and images for: Cooperative Catalytic Coupling of Benzyl Chlorides and Bromides with Electron-Deficient Alkenes
Source: Org Lett. 2024 Jun 19;26(25):5248–52. doi: 10.1021/acs.orglett.4c01413 (PMC11217938; doi:10.1021/acs.orglett.4c01413)

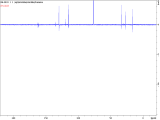

Supplement: Supplementary file 2 — ol4c01413_si_002.zip [file ol4c01413_si_002.zip › FID for publication/3a/Primary_NMR_data_files/13C/1/pdata/1/thumb.png]
