# Supplementary material for: Cooperative Catalytic Coupling of Benzyl Chlorides and Bromides with Electron-Deficient Alkenes
Source: Org Lett. 2024 Jun 19;26(25):5248–52. doi: 10.1021/acs.orglett.4c01413 (PMC11217938; doi:10.1021/acs.orglett.4c01413)
Supplement: Supplementary file 2 — ol4c01413_si_002.zip [file ol4c01413_si_002.zip › FID for publication/3a/Primary_NMR_data_files/19F/11/pdata/1/email_RH-3033_11_1.pdf]

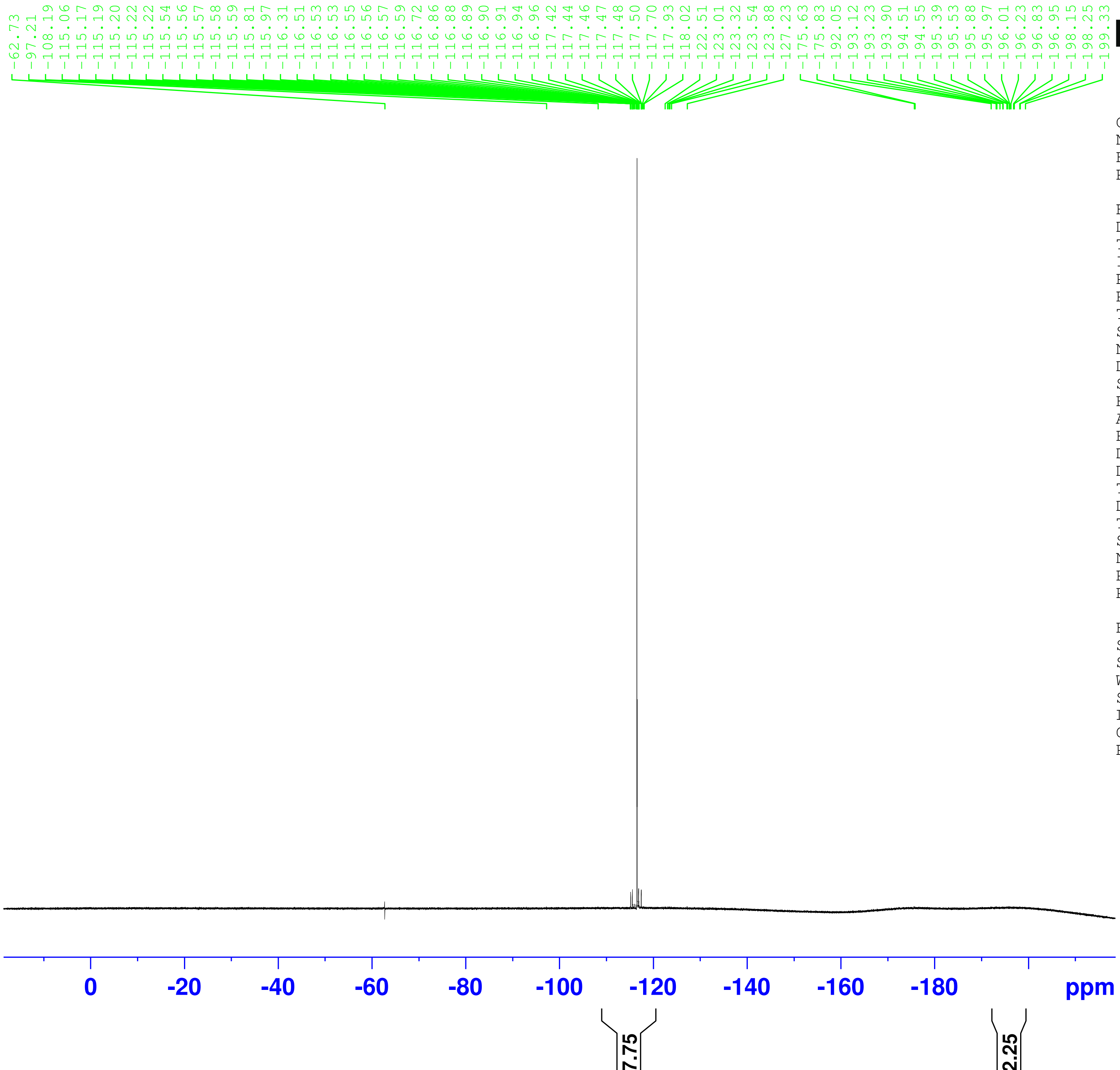

BRUKER

Current Data Parameters
NAME RH-3033
EXPNO 11
PROCNO 1

F2 - Acquisition Parameters
Date_ 20231127
Time 10.43 h
INSTRUM spect
PROBHD Z116098_0222 (
PULPROG zgflqn
TD 131072
SOLVENT CDCl3
NS 16
DS 4
SWH 89285.711 Hz
FIDRES 1.362392 Hz
AQ 0.7340032 sec
RG 206.31
DW 5.600 usec
DE 6.50 usec
TE 298.0 K
D1 5.00000000 sec
TD0 1
SFO1 376.4795333 MHz
NUC1 19F
P1 14.00 usec
PLW1 22.00000000 W

F2 - Processing parameters
SI 65536
SF 376.5171850 MHz
WDW EM
SSB 0
LB 0.30 Hz
GB 0
PC 1.00
