# Supplementary material for: Cooperative Catalytic Coupling of Benzyl Chlorides and Bromides with Electron-Deficient Alkenes
Source: Org Lett. 2024 Jun 19;26(25):5248–52. doi: 10.1021/acs.orglett.4c01413 (PMC11217938; doi:10.1021/acs.orglett.4c01413)
Supplement: Supplementary file 2 — ol4c01413_si_002.zip [file ol4c01413_si_002.zip › FID for publication/3b/Primary_NMR_data_files/19F/10/pdata/1/email_RH-3021-19F_10_1.pdf]

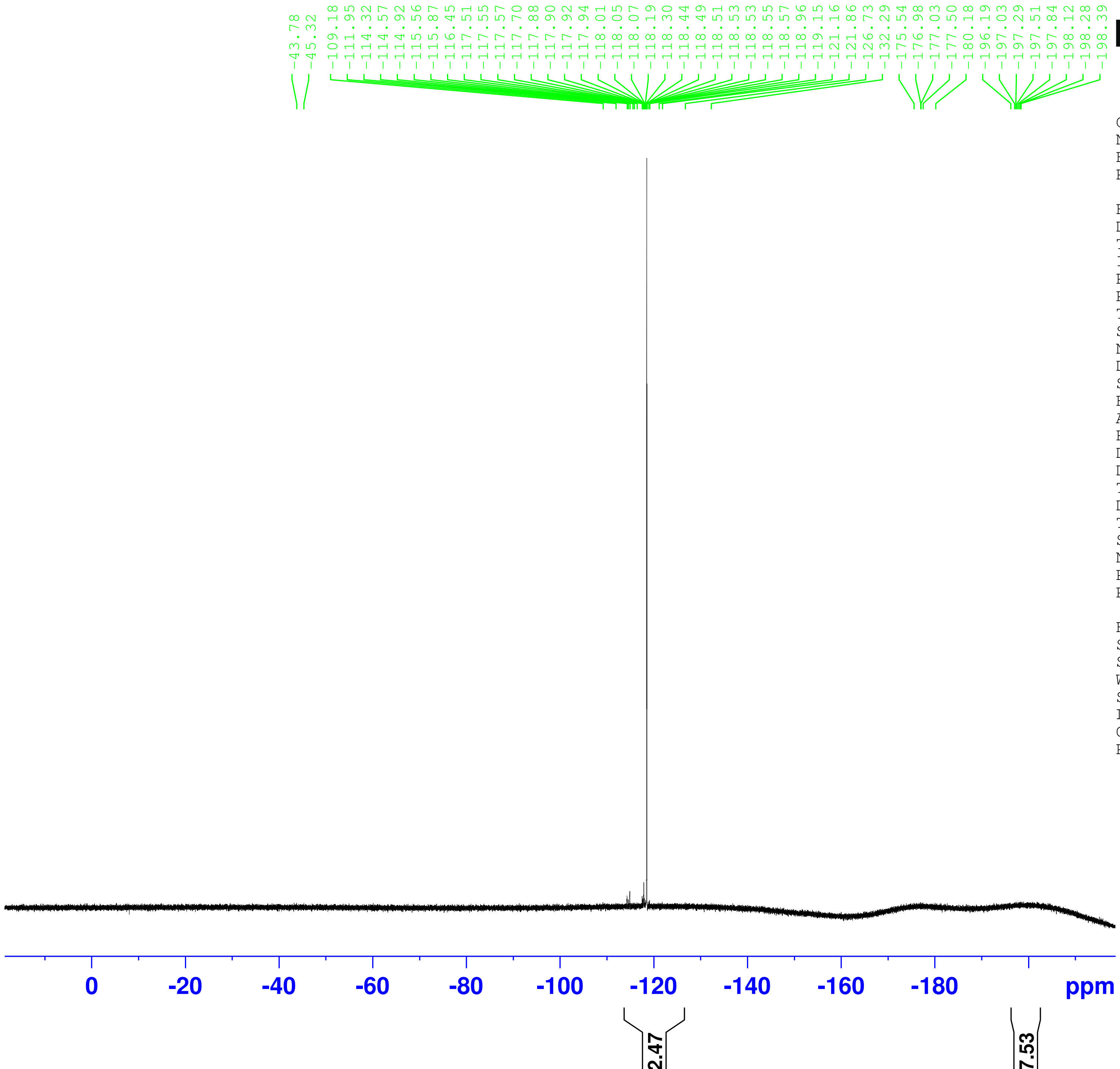

BRUKER

```
Current Data Parameters
NAME           RH-3021-19F
EXPNO                   10
PROCNO                   1

F2 - Acquisition Parameters
Date_             20231026
Time                 12.33 h
INSTRUM              spect
PROBHD      Z116098_0222 (
PULPROG             zgflqn
TD                  131072
SOLVENT              CDCl3
NS                      16
DS                       4
SWH              89285.711 Hz
FIDRES            1.362392 Hz
AQ               0.7340032 sec
RG                  206.31
DW                   5.600 usec
DE                    6.50 usec
TE                   298.0 K
D1              5.00000000 sec
TD0                      1
SFO1           376.4795333 MHz
NUC1                   19F
P1                   14.00 usec
PLW1           22.00000000 W

F2 - Processing parameters
SI                   65536
SF             376.5171850 MHz
WDW                     EM
SSB       0
LB                    0.30 Hz
GB        0
PC                    1.00
```
