# Supplementary material for: Cooperative Catalytic Coupling of Benzyl Chlorides and Bromides with Electron-Deficient Alkenes
Source: Org Lett. 2024 Jun 19;26(25):5248–52. doi: 10.1021/acs.orglett.4c01413 (PMC11217938; doi:10.1021/acs.orglett.4c01413)
Supplement: Supplementary file 2 — ol4c01413_si_002.zip [file ol4c01413_si_002.zip › FID for publication/3d/Primary_NMR_data_files/13C/20/pdata/1/email_RH-3045-p-2_20_1.pdf]

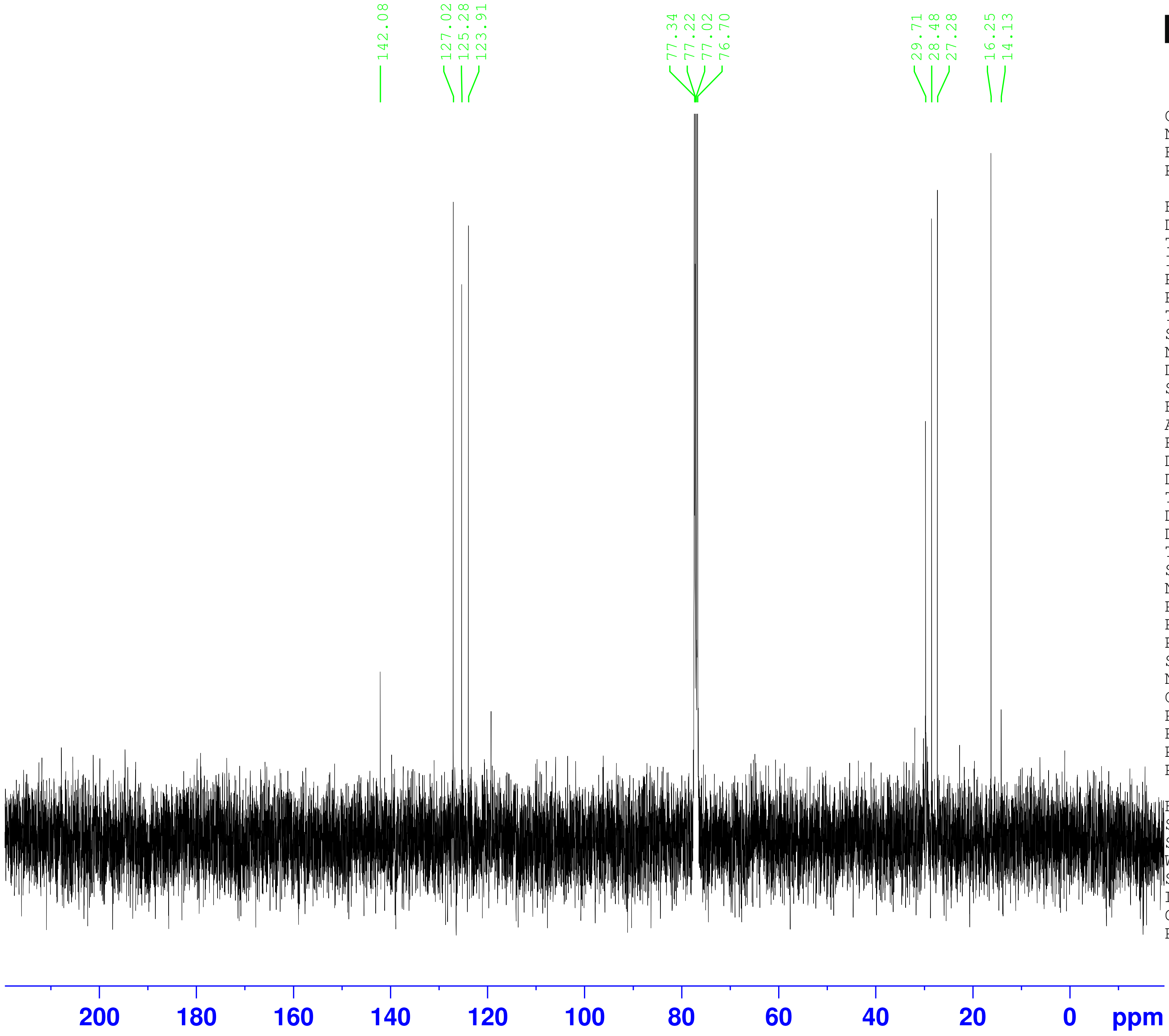

BRUKER

Current Data Parameters
NAME RH-3045-p-2
EXPNO 20
PROCNO 1

F2 - Acquisition Parameters
Date_ 20240104
Time 1.01 h
INSTRUM spect
PROBHD Z116098_0222 (
PULPROG zgpg30
TD 65536
SOLVENT CDCl3
NS 1024
DS 4
SWH 24038.461 Hz
FIDRES 0.733596 Hz
AQ 1.3631488 sec
RG 206.31
DW 20.800 usec
DE 6.50 usec
TE 298.0 K
D1 2.00000000 sec
D11 0.03000000 sec
TD0 1
SFO1 100.6278593 MHz
NUC1 13C
P0 3.33 usec
P1 10.00 usec
PLW1 70.00000000 W
SFO2 400.1516006 MHz
NUC2 1H
CPDPRG[2 waltz65
PCPD2 90.00 usec
PLW2 13.89000034 W
PLW12 0.17148000 W
PLW13 0.08625400 W

F2 - Processing parameters
SI 32768
SF 100.6177975 MHz
WDW EM
SSB 0
LB 1.00 Hz
GB 0
PC 1.40
