# Supplementary material for: Cooperative Catalytic Coupling of Benzyl Chlorides and Bromides with Electron-Deficient Alkenes
Source: Org Lett. 2024 Jun 19;26(25):5248–52. doi: 10.1021/acs.orglett.4c01413 (PMC11217938; doi:10.1021/acs.orglett.4c01413)
Supplement: Supplementary file 2 — ol4c01413_si_002.zip [file ol4c01413_si_002.zip › FID for publication/3e/Primary_NMR_data_files/1H/10/pdata/1/email_RH-3001-col_10_1.pdf]

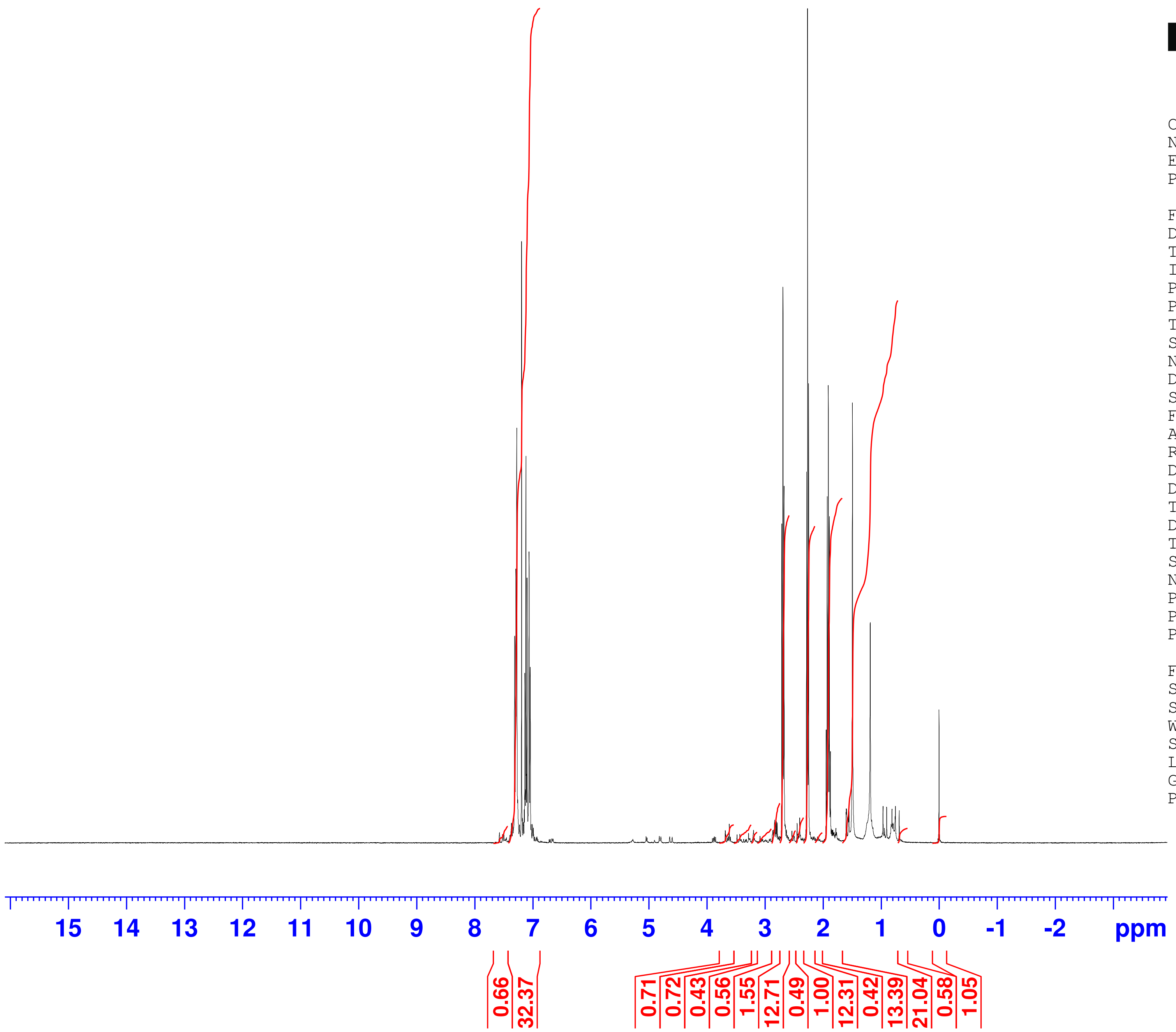

BRUKER

```
Current Data Parameters
NAME          RH-3001-col
EXPNO                  10
PROCNO                  1

F2 - Acquisition Parameters
Date_            20231009
Time                19.23 h
INSTRUM             spect
PROBHD   Z116098_0222 (
PULPROG              zg30
TD                  65536
SOLVENT             CDCl3
NS                     16
DS                      2
SWH              8012.820 Hz
FIDRES           0.244532 Hz
AQ              4.0894465 sec
RG                 124.73
DW                 62.400 usec
DE                   6.50 usec
TE                  298.0 K
D1             1.00000000 sec
TD0                     1
SFO1          400.1524709 MHz
NUC1                   1H
P0                   3.33 usec
P1                  10.00 usec
PLW1          13.89000034 W

F2 - Processing parameters
SI                  65536
SF            400.1500375 MHz
WDW                    EM
SSB      0
LB                   0.30 Hz
GB       0
PC                   1.00
```
