# Supplementary material for: Cooperative Catalytic Coupling of Benzyl Chlorides and Bromides with Electron-Deficient Alkenes
Source: Org Lett. 2024 Jun 19;26(25):5248–52. doi: 10.1021/acs.orglett.4c01413 (PMC11217938; doi:10.1021/acs.orglett.4c01413)
Supplement: Supplementary file 2 — ol4c01413_si_002.zip [file ol4c01413_si_002.zip › FID for publication/3j/Primary_NMR_data_files/1H/10/pdata/1/email_RH-3048-f-25_10_1.pdf]

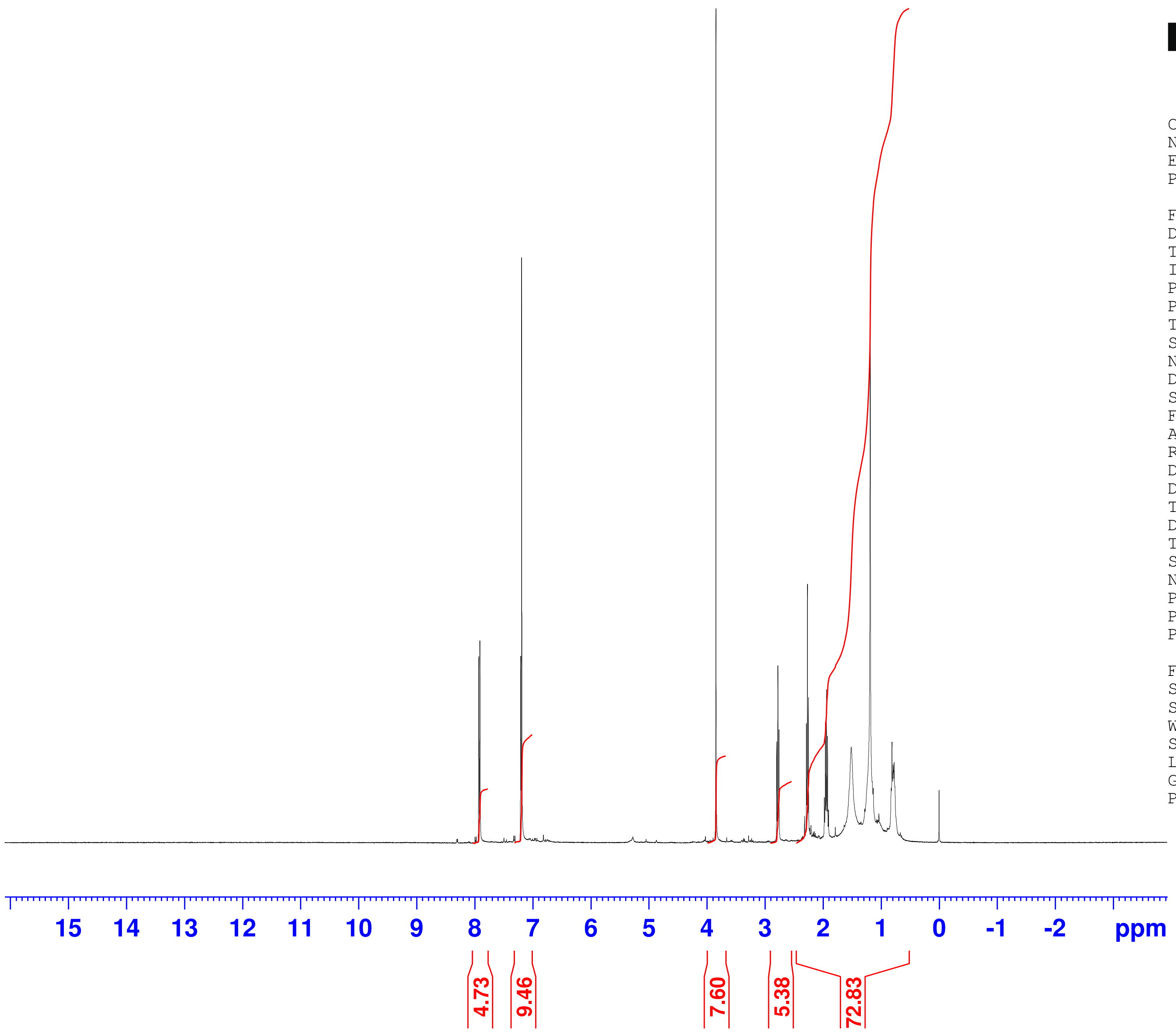

Current Data Parameters

NAME	RH-3048-f-25
EXPNO	10
PROCNO	1

F2 - Acquisition Parameters

Date_	20240521
Time	18.45 h
INSTRUM	spect
PROBHD	Z116098_0222 (
PULPROG	zg30
TD	65536
SOLVENT	CDCl3
NS	16
DS	2
SWH	8012.820 Hz
FIDRES	0.244532 Hz
AQ	4.0894465 sec
RG	81.62
DW	62.400 usec
DE	6.50 usec
TE	298.0 K
D1	1.00000000 sec
TD0	1
SFO1	400.1524709 MHz
NUC1	1H
P0	3.33 usec
P1	10.00 usec
PLW1	13.89000034 W

F2 - Processing parameters

SI	65536
SF	400.1500372 MHz
WDW	EM
SSB	0
LB	0.30 Hz
GB	0
PC	1.00
