# Supplementary material for: Cooperative Catalytic Coupling of Benzyl Chlorides and Bromides with Electron-Deficient Alkenes
Source: Org Lett. 2024 Jun 19;26(25):5248–52. doi: 10.1021/acs.orglett.4c01413 (PMC11217938; doi:10.1021/acs.orglett.4c01413)
Supplement: Supplementary file 2 — ol4c01413_si_002.zip [file ol4c01413_si_002.zip › FID for publication/3m/Primary_NMR_data_files/13C/11/pdata/1/email_RH-3002-col_11_1.pdf]

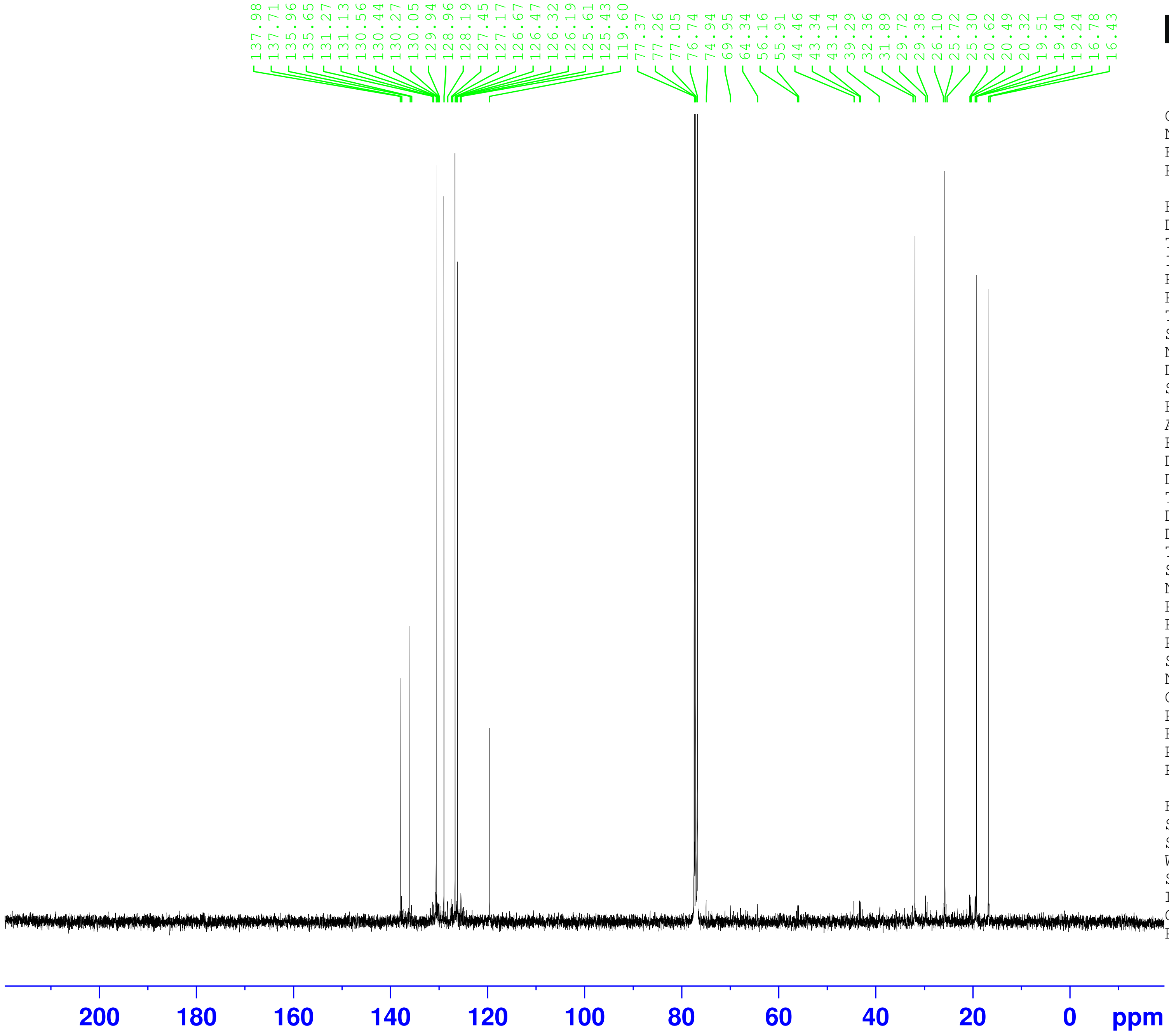


Current Data Parameters

| | | |
|---|---|---|
| NAME | RH-3002-col | |
| EXPNO | 11 | |
| PROCNO | 1 | |

F2 - Acquisition Parameters

| | | |
|---|---|---|
| Date_ | 20231010 | |
| Time | 1.02 | h |
| INSTRUM | spect | |
| PROBHD | Z116098_0222 ( | |
| PULPROG | zgpg30 | |
| TD | 65536 | |
| SOLVENT | CDCl3 | |
| NS | 1024 | |
| DS | 4 | |
| SWH | 24038.461 | Hz |
| FIDRES | 0.733596 | Hz |
| AQ | 1.3631488 | sec |
| RG | 206.31 | |
| DW | 20.800 | usec |
| DE | 6.50 | usec |
| TE | 298.0 | K |
| D1 | 2.00000000 | sec |
| D11 | 0.03000000 | sec |
| TD0 | 1 | |
| SFO1 | 100.6278593 | MHz |
| NUC1 | 13C | |
| P0 | 3.33 | usec |
| P1 | 10.00 | usec |
| PLW1 | 70.00000000 | W |
| SFO2 | 400.1516006 | MHz |
| NUC2 | 1H | |
| CPDPRG[2 | waltz65 | |
| PCPD2 | 90.00 | usec |
| PLW2 | 13.89000034 | W |
| PLW12 | 0.17148000 | W |
| PLW13 | 0.08625400 | W |

F2 - Processing parameters

| | | |
|---|---|---|
| SI | 32768 | |
| SF | 100.6177975 | MHz |
| WDW | EM | |
| SSB | 0 | |
| LB | 1.00 | Hz |
| GB | 0 | |
| PC | 1.40 | |
