# Supplementary material for: Cooperative Catalytic Coupling of Benzyl Chlorides and Bromides with Electron-Deficient Alkenes
Source: Org Lett. 2024 Jun 19;26(25):5248–52. doi: 10.1021/acs.orglett.4c01413 (PMC11217938; doi:10.1021/acs.orglett.4c01413)
Supplement: Supplementary file 2 — ol4c01413_si_002.zip [file ol4c01413_si_002.zip › FID for publication/3u/Primary_NMR_data_files/19F/10/pdata/1/email_RH-3063-19F_10_1.pdf]

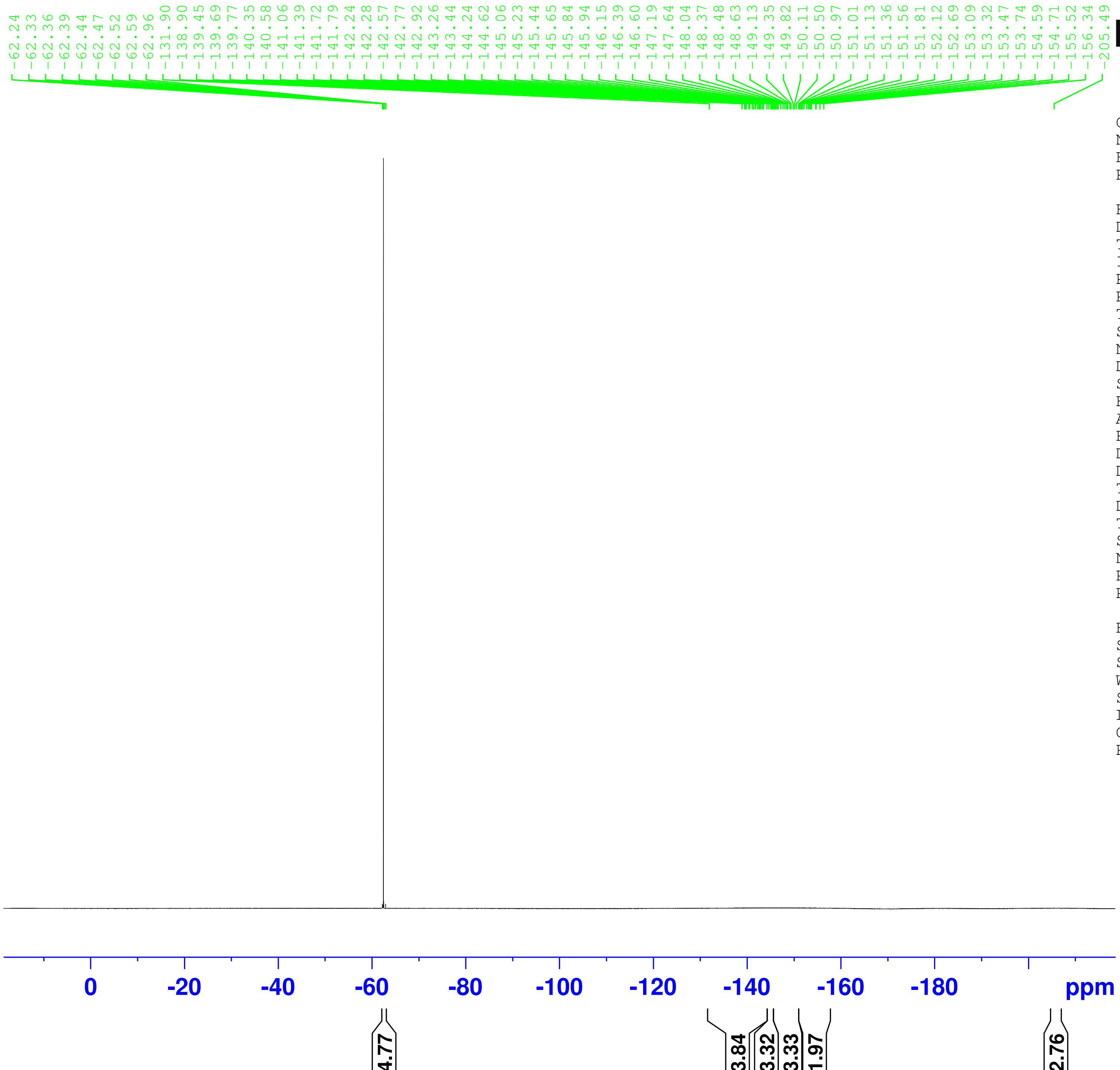

Current Data Parameters

NAME	RH-3063-19F
EXPNO	10
PROCNO	1

F2 - Acquisition Parameters

Date_	20240125
Time	16.57 h
INSTRUM	spect
PROBHD	Z116098_0222 (
PULPROG	zgflqn
TD	131072
SOLVENT	CDCl3
NS	16
DS	4
SWH	89285.711 Hz
FIDRES	1.362392 Hz
AQ	0.7340032 sec
RG	206.31
DW	5.600 usec
DE	6.50 usec
TE	298.0 K
D1	1.00000000 sec
TD0	1
SFO1	376.4795333 MHz
NUC1	19F
P1	14.00 usec
PLW1	22.00000000 W

F2 - Processing parameters

SI	65536
SF	376.5171850 MHz
WDW	EM
SSB	0
LB	0.30 Hz
GB	0
PC	1.00
