# Supplementary material for: Cooperative Catalytic Coupling of Benzyl Chlorides and Bromides with Electron-Deficient Alkenes
Source: Org Lett. 2024 Jun 19;26(25):5248–52. doi: 10.1021/acs.orglett.4c01413 (PMC11217938; doi:10.1021/acs.orglett.4c01413)
Supplement: Supplementary file 2 — ol4c01413_si_002.zip [file ol4c01413_si_002.zip › FID for publication/3w/Primary_NMR_data_files/1H/10/pdata/1/email_RH-2016-prep-3_10_1.pdf]

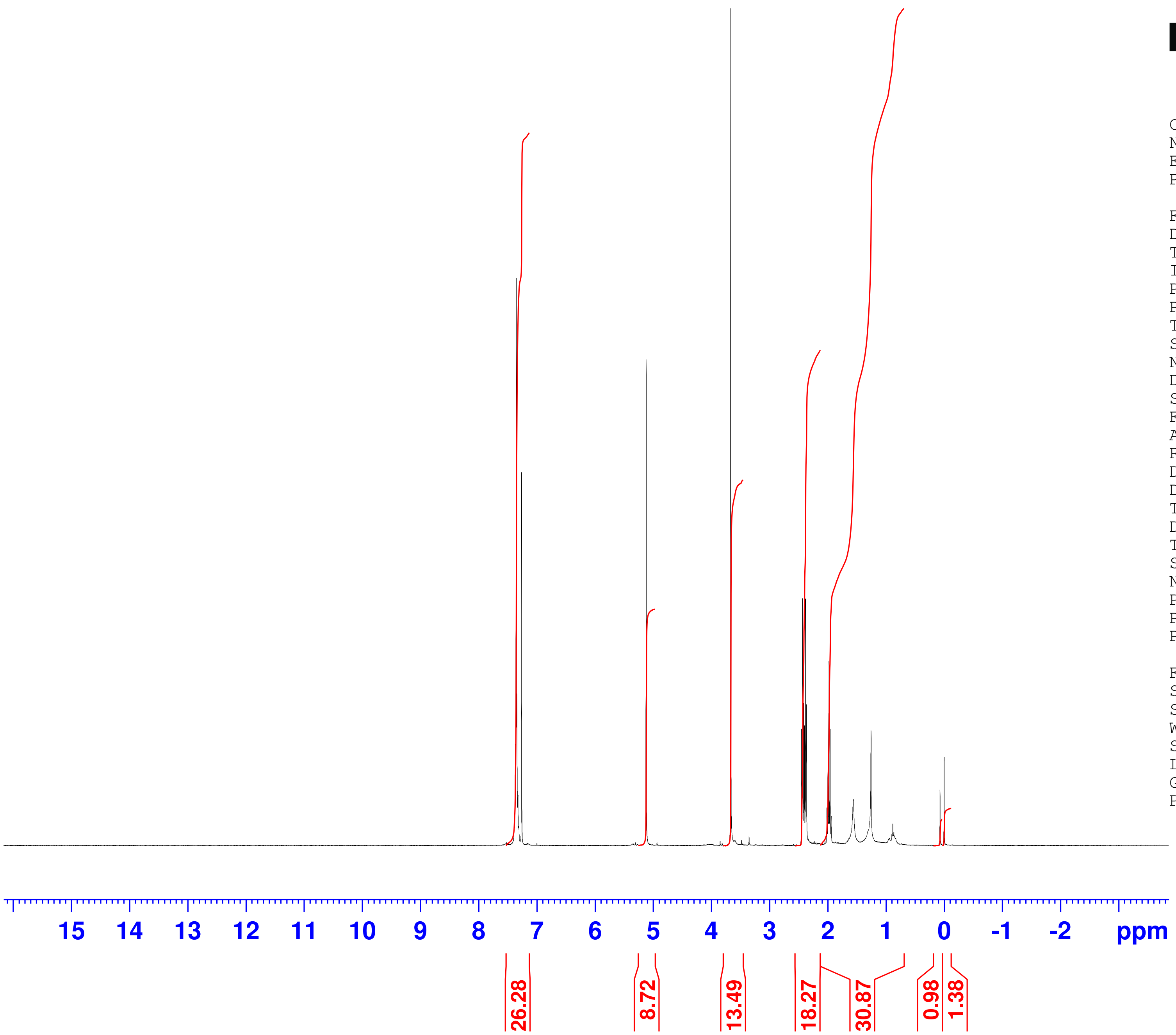

BRUKER

```
Current Data Parameters
NAME      RH-2016-prep-3
EXPNO                 10
PROCNO                 1

F2 - Acquisition Parameters
Date_           20230210
Time               13.32 h
INSTRUM            spect
PROBHD    Z116098_0222 (
PULPROG             zg30
TD                 65536
SOLVENT            CDCl3
NS                    16
DS                     2
SWH             8012.820 Hz
FIDRES          0.244532 Hz
AQ             4.0894465 sec
RG                 81.62
DW                62.400 usec
DE                  6.50 usec
TE                 298.0 K
D1            1.00000000 sec
TD0                    1
SFO1         400.1524709 MHz
NUC1                  1H
P0                  3.33 usec
P1                 10.00 usec
PLW1         13.89000034 W

F2 - Processing parameters
SI                 65536
SF           400.1500096 MHz
WDW                   EM
SSB       0
LB                  0.30 Hz
GB        0
PC                  1.00
```
